# Further exploration of the heterocyclic diversity accessible from the allylation chemistry of indigo

**DOI:** 10.3762/bjoc.11.54

**Published:** 2015-04-15

**Authors:** Alireza Shakoori, John B Bremner, Mohammed K Abdel-Hamid, Anthony C Willis, Rachada Haritakun, Paul A Keller

**Affiliations:** 1 School of Chemistry, University of Wollongong, Wollongong, NSW, 2522, Australia; 2 School of Chemistry, The Australian National University, Canberra, ACT 0200, Australia; 3 National Centre for Genetic Engineering and Biotechnology (BIOTEC), National Science and Technology Development Agency (NSTDA), 113 Phaholyothin Road, Klong1, Klong Luang, Pathumanthani 12120, Thailand

**Keywords:** allylation, cascade reactions, indigo, nitrogen heterocycles, rearrangement

## Abstract

Diversity-directed synthesis based on the cascade allylation chemistry of indigo, with its embedded 2,2’-diindolic core, has resulted in rapid access to new examples of the hydroxy-8a,13-dihydroazepino[1,2-*a*:3,4-*b*']diindol-14(8*H*)-one skeleton in up to 51% yield. Additionally a derivative of the novel bridged heterocycle 7,8-dihydro-6*H*-6,8a-epoxyazepino[1,2-*a*:3,4-*b*']diindol-14(13*H*)-one was produced when the olefin of the allylic substrate was terminally disubstituted. Further optimisation also produced viable one-pot syntheses of derivatives of the spiro(indoline-2,9'-pyrido[1,2-*a*]indol)-3-one (65%) and pyrido[1,2,3-*s*,*t*]indolo[1,2-*a*]azepino[3,4-*b*]indol-17-one (72%) heterocyclic systems. Ring-closing metathesis of the *N*,*O*-diallylic spiro structure and subsequent Claisen rearrangement gave rise to the new (1*R*,8a*S*,17a*S*)-rel-1,2-dihydro-1-vinyl-8*H*,17H,9*H*-benz[2',3']pyrrolizino[1',7*a*':2,3]pyrido[1,2-*a*]indole-8,17-(2*H*,9*H*)-dione heterocyclic system.

## Introduction

One of the foci of current organic synthesis is the exploration of new chemical space, with an emphasis on significant heterocyclic molecular diversity [1–2]. Direct applications of such advances in medicinal chemistry, chemical biology and nanochemistry should provide expanded opportunities for helping to solve major, real-world problems. Additional to the discovery of representatives in new chemical space must be the technology to produce the heterocycles in a controlled, scalable and cost-effective manner. To this end, one approach that we have explored is to take cheap and readily available structurally advanced starting materials and induce cascade reactions to produce relatively complex heterocycles. For example, initial exploratory studies on the base-induced N-alkylation chemistry of readily available indigo (**1**) with functionalised alkyl halides has highlighted the potential for the rapid generation of poly-heterocyclic skeletons when the halides incorporate double or triple bond moieties [3–4]. Thus propargyl bromide with indigo (**1**) provided access to representatives of the pyrazino[1,2-*a*:4,3-*a*']diindole, pyrido[1,2-a:3,4-*b*']diindole and benzo[*b*]indolo[1,2-*h*]naphthyridine heterocycles in one-pot, multi-step cascade sequences [3]. These ring systems are not only of chemical interest but biological testing revealed promising in vitro antiplasmodial activity and anticancer activity in certain cases [3].

While the analogous base-mediated thermal reactions of allylic bromides with **1** at 70 °C were promising and allowed facile, but limited, access to new 1-allyl-5'-allyloxy-3',4'-dihydrospiroindoline-pyrido[1,2-*a*]indolone derivatives and pyridoindolo-azepino[1,2-*a*]indol-11(7*H*)-one derivatives, the reactions were incomplete, yields were modest, and oxidative isatin byproducts were significant [4].

Thus these reactions of indigo (**1**) with allylic halides were further explored, not only in an effort to gain better control over reaction pathways, but to optimise the synthesis of known polycyclic ring systems to practicable yields, and to assess other products produced, including any new heterocyclic systems produced.

Therefore, this manuscript describes procedures for the highly controlled one-pot cascade reactions of indigo with variations of the allylic bromides used and reaction times. The outcomes included the synthesis of unprecedented heterocycles, optimised procedures of some previously reported structures to now produce synthetically useful yields, and the efficient syntheses of additional polycyclic heterocycles. These results lead to substantial new mechanistic insights, an assessment of the potential to further increase structural complexity through post-allylation ring-closing metathesis, plus new biological activity investigations, which are now reported herein.

## Results and Discussion

A range of strategies are available to potentially control reaction-path selectivity in cascade pathways [5–6] but in these indigo allylation reactions it was found that reaction temperature and reaction time were particularly important together with the nature of the allylic bromide. A higher temperature (85–88 °C) than that used previously was found to be optimal with the eventual complete consumption of the indigo starting material. At this temperature, reaction times were then explored and the product outcomes are discussed in the context of the times used, in this study short (5 s), medium (1 h) or long (3 h).

### *N*-Monoallylation – short reaction time

**Scheme 1:**
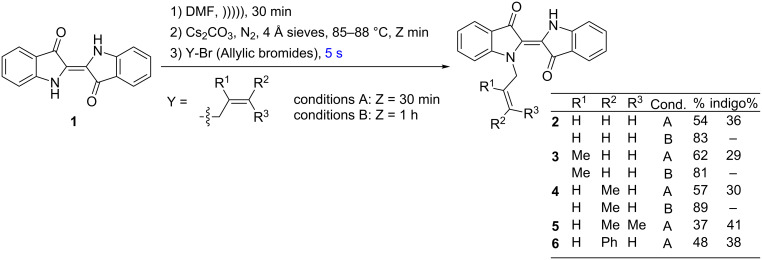
The synthesis of *N*-monoallylated indigo derivatives.

In a typical procedure, a solution of indigo (**1**) in anhydrous DMF was generated through sonication for 30 min at room temperature and then transferred to a septum-equipped flask which contained predried molecular sieves and caesium carbonate under an inert atmosphere. The flask was then plunged into a preheated oil bath (strictly 85–88 °C) and stirred for 30 min, followed by the addition of various allylic bromides by injection through a septum under a static nitrogen blanket and the reaction mixture then heated at this temperature for 5 seconds (Scheme 1). This yielded the *N*-monoallylated indigos **2**–**6** as the dominant products in yields of 37–62% as papery blue solids. The structure about the 2,2’-double bond of these compounds was presumed to be *transoid* based on the retention of the intense blue colour which arises from the presence of at least one H-bond between the NH and carbonyl, as well as the absence of a nOe interaction between the N-methylene and the NH present; this was confirmed by an X-ray structure for **2**. These compounds also had a significantly higher solubility in organic solvents relative to the parent indigo starting material. Although indigo starting material also remained at the end of the reaction, it could be readily separated on the basis of solvent solubility.

These monoallylated compounds are of particular importance in that the improved solubility allows for a greater range of reaction solvents for subsequent reactions. Therefore, the procedure for their synthesis was optimised by allowing a greater time for the initial generation of the anion (1 h vs 30 min) – this resulted in an increase in yield to 83%, 81% and 89% for the monoallylated indigos **2**, **3** and **4**, respectively.

### Synthesis of azepinodiindolones – medium reaction time

**Scheme 2:**
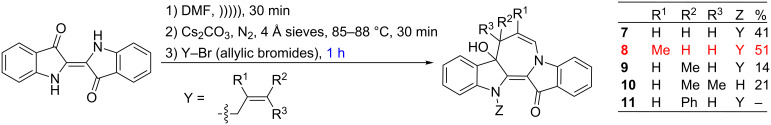
The synthesis of 8a-hydroxyazepinodiindolones.

Under identical reaction conditions to those reported above, with the exception of a one–hour reaction time after addition of the allyl bromides, the major products isolated were new derivatives of the fused azepinodiindolo system [5], the 8a-hydroxy azepinodiindolones **7**–**10** (Scheme 2). While minor yields of these red compounds (7–10%) were obtained after reaction for 5 s, optimal yields occurred after 1 h. Interestingly cinnamyl bromide did not produce any of this ring system [7], while for 1,1-dimethylallyl bromide further *N*-allylation was not evident.

**Figure 1:**
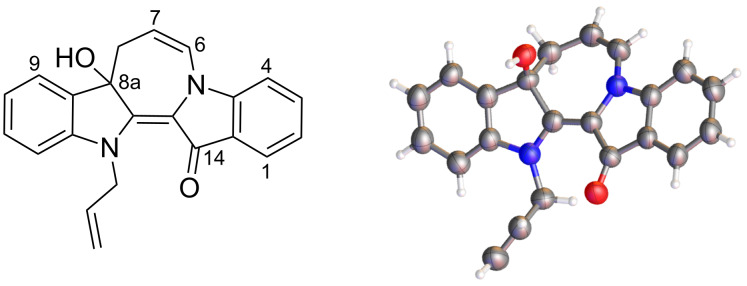
The structure and X-ray crystal structure (ball and stick representation) of azepinodiindolone **7**. The corresponding ORTEP data for **7**, **8** and **9** is reported in Supporting Information File 1.

Analysis of the ^1^H NMR spectrum of **7** revealed a peak at 4.73 ppm assigned to the alcohol – this peak disappeared upon treatment of the sample with D_2_O. The quaternary benzylic C8a (Figure 1) was assigned to the peak at 81.3 ppm in the ^13^C NMR spectrum, downfield due to the effects of the attached alcohol substituent and its benzylic/allylic nature. Analysis of the HMBC spectra revealed strong correlations between H9 and C8a, H8 and C8a, and a weaker correlation between H6 and C4a. The structures of **7** (Figure 1), **8** and **9** were confirmed by X-ray crystallography analysis.

Azepinodiindolones **7** and **8** were obtained in moderate yields. The addition of substituents in the terminal position of the allyl substrate resulted in a lowering of yield of the analogous products **9** and **10**, while no such product was evident from the reaction with the larger cinnamyl bromide. Minor byproducts isolated were the corresponding *N*-monoallylated indigos (7–15%).

These heterocycles arise from the cyclisation of one allyl unit onto a carbonyl, leading to the tertiary alcohol. They are deep red in colour, probably due to the central double bond remaining intact while one of the indigo carbonyl units has been transformed. Theoretically, diastereomers are possible due to the presence of the stereogenic C8a carbon and an atropisomeric chiral element from restricted rotation in the 7-membered ring [8]. However, analysis of the ^13^C NMR spectra of these products indicated only a single set of peaks in each case and no evidence for a diastereomeric mixture. Presumably, the atropisomeric rotation barrier is sufficiently low at room temperature to allow for interconversion.

**Figure 2:**
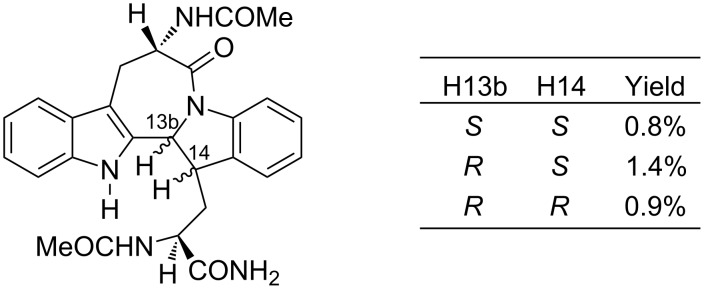
Dihydroazepino[1,2-*a*:3,4-*b*']diindolones from *N*_b_-acyl-L-tryptophanamides [5].

While derivatives of the azepino[1,2-*a*:3,4-*b*']diindolone skeleton have been reported previously, albeit in a different oxidation state and with notably different substitution patterns [5] (Figure 2), they were only obtained as very minor diastereomeric products (ca 1% yields) from acid-catalysed cyclisation studies on *N*_b_-acyl-L-trypotophanamides.

### Three–hour reactions of indigo with allyl bromides

**Scheme 3:**
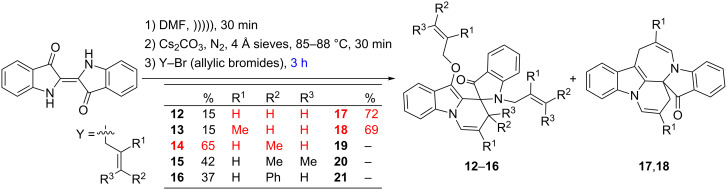
The optimal synthesis of spiro heterocycles **12**–**16**, and spiro/polyfused ring heterocycles **17** and **18** (R^2^, R^3^ omitted for clarity in **17**, **18**).

Analysis of the product outcomes of the corresponding 3 hour reactions of indigo with different allyl bromides (Scheme 3) confirmed the synthesis of the known 8*H*,16*H*-pyrido[1,2,3-*s*,*t*]indolo[1,2-*a*]azepino[3,4-*b*]indol-17-one heterocycles **17** and **18**, which arise from the addition of two allyl units with one cyclising to form a 6-membered ring and the other to form a 7-membered ring [4]. These yields are now excellent for the production of these reasonably complex heterocycles, around 70% for **17** and **18**. Notably, the olefinic terminal position remains unsubstituted to achieve these outcomes. The addition of substituents to the terminal positions of the allyl reagents (crotyl, 1,1-dimethylallyl, cinnamyl) resulted in a change in the major product outcome to the known spiro heterocycles **14**, **15** and **16**, although with a decreasing absolute yield (65% to 37%) with increasing steric presence; none of the fused azepinoindolones (designated **19**–**21**) analogous to **17** and **18** were obtained. The yield of 37% obtained for the cinnamyl derivative **16** is a significant improvement on that (12%) previously reported [4] and the analogous derivative **15** is reported for the first time. The previously reported low yields of these spiro-based heterocycles were accompanied by significant quantities of indigo starting material. These reactions are also scalable at least up to 1 gram quantities of indigo starting material to produce a 72% yield of **17** – this is a four-fold increase in quantity over the standard reactions quantities.

### Additional outcomes of reaction of indigo with allyl bromides

**Scheme 4:**
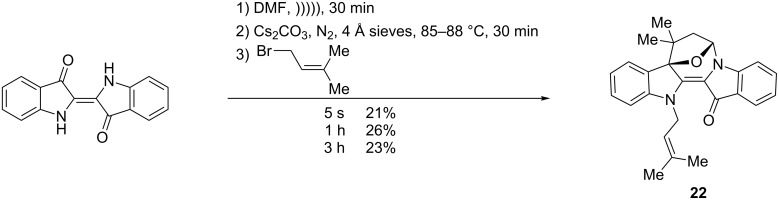
The synthesis of the oxa-bridged azepinodiindolone **22** from indigo and 1-bromo-3-methylbut-2-ene.

**Figure 3:**
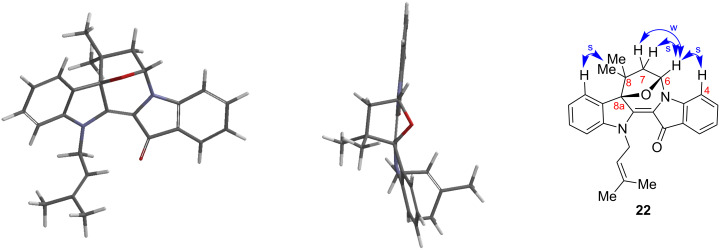
Modelled structure (Spartan 10, v1.1.0, Wavefunction Inc) of bridged indigo-tetrahydrofuran product **22**. Left: top view showing the tetrahydrofuran ring sitting directly over the indigo skeleton (vertical), which itself is twisted from planarity. Middle: front view. Right: NOESY correlations in support of the proposed structure (s = strong; w = weak).

Interestingly, in all cases with the use of 4-bromo-3-methylbut-ene (5 s, 1 h, 3 h), an additional novel heterocyclic derivative was isolated in 21, 26 and 23% yields, respectively (Scheme 4). This red compound showed the addition of two allylic systems, with one cyclised, with the intense colour suggesting that the central double bond was intact and with extended conjugation present. The ^13^C NMR spectrum contained one peak in the region at 177.5 ppm, corresponding to the presence of one carbonyl functionality. The IR spectrum was notable for the absence of a broad absorption band in the 3300 cm^−1^ region, implying the absence of an alcohol group, despite the red colour suggesting that one of the allylic substitutions had cyclised, most likely onto a carbonyl. Key to the structural elucidation were NOESY experiments which showed correlations between the aromatic H4 proton and the H6 bridgehead proton (Figure 3) – the same H6 proton correlated strongly with one H7 and weakly with the second H7 proton suggesting a –CH_2_–CH– arrangement in a conformationally restricted ring, with the weak correlation assigned to the *transoid* arrangement of protons and the strong correlation to the *cisoid* configuration. In addition, significant 3-bond correlations in the HMBC spectrum were observed between H6 and the quaternary C8 and H7 and the benzylic C8a. The downfield shift of the peak at 92.7 ppm, assigned to C8a, is consistent with the benzylic carbon being also attached to an oxygen atom. This new heterocyclic product was thus assigned as the oxa-bridged azepinodiindolone **22**. The geometry optimised structure for **22** (Figure 3) showed the two indolic units to be in a slightly bent orientation to each other, with the 7-membered ring in the same curved line. The 5-membered tetrahydrofuryl ring is disposed orthogonally to the curved backbone and is in a puckered conformation, typical of 5-membered aliphatic rings. The heterocycle is stable at room temperature and to air and moisture. This is the first example of the synthesis of this bridged heterocyclic skeleton.

The formation of **22** is an excellent example of the ‘*gem*-dimethyl effect’ [9] whereby the presence of that moiety enhances cyclisation [10]. This explains the formation of **22** and the lack analogous cyclised products when the *gem*-dimethyl group was absent, as evidenced by the absence of distinctive red-coloured products by TLC analysis. Interesting in this particular reaction is the induction of the *gem*-dimethyl effect in producing a bridged product from a cyclic starting material. Further evidence in support of the bicyclic heterocycle **22** comes from analysis of computational models of the proposed starting materials to cyclisation (i.e., alcohol **10**) and the bridged products, e.g., **22** (Table 1). The Δ*H*_f_ of the *gem*-dimethyl substrate **10** (262.7 kJ/mol) is remarkably similar to the product bicycle **22** (263.0 kJ/mol) whereas the Δ*H*_f_ for the corresponding theoretical bridged products (295.3 kJ/mol and 351.2 kJ/mol) arising from cyclisation of monomethyl (**9**; 289.2 kJ/mol) and 8-methylene (**7**; 338.3 kJ/mol) starting materials respectively, are of higher energy. Further, the distance between the proposed O-nucleophile and C6-imine electrophile in the proposed intermediates to cyclisation (see structure **27** in Scheme 7) is least for the *gem*-dimethyl compound **10** (3.042 Å) and larger for the monomethyl compound **9** (3.056 Å) and **7** (3.180 Å).

**Table 1:** Calculated values of Δ*H*_f_ of the red diindolone substrate **10** and the bridged tetrahydrofuran product **22**, plus the distance between nucleophile (O) and electrophile (C) in the proposed key reaction intermediate, structure **27**, together with comparison data for analogous structures from **9** and **7**.

C8 substitution pattern	Δ*H*_f_ substrate (diindolones) kJ/mol	Δ*H*_f_ cyclic aminals (bridged tetrahydrofurans) kJ/mol	distance between (C8a)O and C6 (Å)

2 × Me (**10**)	262.715	263.010 (**22**)	3.042 (**27**)
1 × Me, 1 × H (**9**)	289.247	295.266	3.056
2 × H (**7**)	338.331	351.245	3.180

**Scheme 5:**
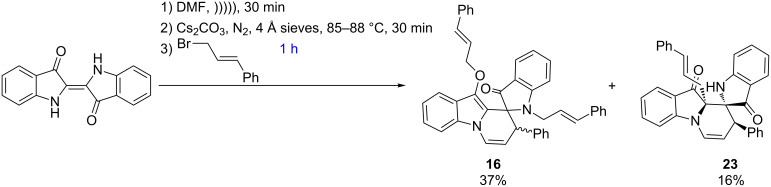
The reaction of indigo with cinnamyl bromide yielding two spiro-based derivatives.

**Figure 4:**
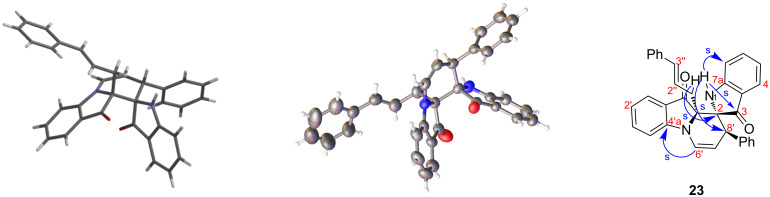
Structural analysis of compound **23**. Left: modelled structure showing the *cisoid* configuration of the NH and the cinnamyl substituents. Middle: X-ray crystal structure (ball and stick representation) showing the *transoid* NH/C8'-phenyl substituent disposition. Right: key HMBC correlations in support of the proposed structure (s = strong; w = weak). ORTEP data for **23** is given in Supporting Information File 1.

With cinnamyl bromide, an additional variation in the product outcome was observed in 16% yield, identified as **23** (Scheme 5) and represents a new variation on the spiro heterocycle of the type exemplified by **16**. This yellow compound corresponded to the addition of two cinnamyl units, with one cyclised to form the parent spiro system. Analysis of the ^13^C NMR spectrum showed the presence of peaks at 197.5 and 197.8 ppm, assigned to two carbonyl groups. This eliminated the substituent pattern as defined by spiro compounds **12**–**16**. The *C*-allylated product **23** was evidenced by analysis of the HMBC spectrum which showed a strong 3-bond correlation between the C2 spiro carbon and the cinnamyl methylene (H1''). Appearance of a multiplet at 2.98–3.10 ppm assigned to the C–CH_2_ revealed the 2.4 ppm shift compared to the chemical shift of deshielded O–CH_2_ protons in the spiro product. Another signal appearing at 7.87 ppm, assigned to an NH, and its nOe correlation with H7 from the aromatic ring confirmed the presence of the non-alkylated isoindoline moiety. The observation of one set of peaks in the ^13^C NMR spectrum indicated the presence of a single enantiomeric pair of isomers, and analysis of the X-ray crystal structure (Figure 4) showed that the relative stereochemistry was either all-*R* or all-*S*. Based on these results the cinnamyl substituent is placed in a *cisoid* orientation to the isooxindolic N–H and the C8' substituent possesses a *transoid* configuration. This configuration allows for the bulky cinnamyl substituent to swing away and be on top of the concave structure.

In summary, a bulky terminal substitution pattern in the allylic bromide reactant is thus an important factor in determining the eventual main product outcomes after *N*-monoallylation.

### Mechanistic discussions

The short reaction times suggest the initial formation of monoallylated products followed by a second *N*-allylation in most cases and cyclisation, although the exact order of these two steps is not apparent. Evidence for the formation of the *N,N*'-diallylated products was observed, e.g., by mass spectral analysis of reaction mixtures, but such products were never isolated. After 5 s reaction time, the red diindolone heterocycles **7**–**10** were already produced, an indication of the ease of the cascade processes. After 1 h reaction time, there was no evidence for the presence of the monoallylindigo products, and the spiro compounds were present in minor quantities. Instead, a greater proportion of the red diindolones was produced. After 3 h reaction time the spiro compounds **12–16** and azepinodiindolones **17**–**18** predominate with a corresponding loss of the monoallylindigos and diindolones. This includes the more sterically demanding cinnamyl bromide and 1-bromo-2-butene which also gave rise to spiro heterocycles (**16** and **15** respectively), the latter reported here for the first time. The products arising from oxidative cleavage, the *N-*allylisatins, are diminished under these optimised conditions, and appear to arise only after longer reaction times. The proposed mechanism for the formation of spiro derivatives **12**–**16** was previously reported [4].

**Scheme 6:**
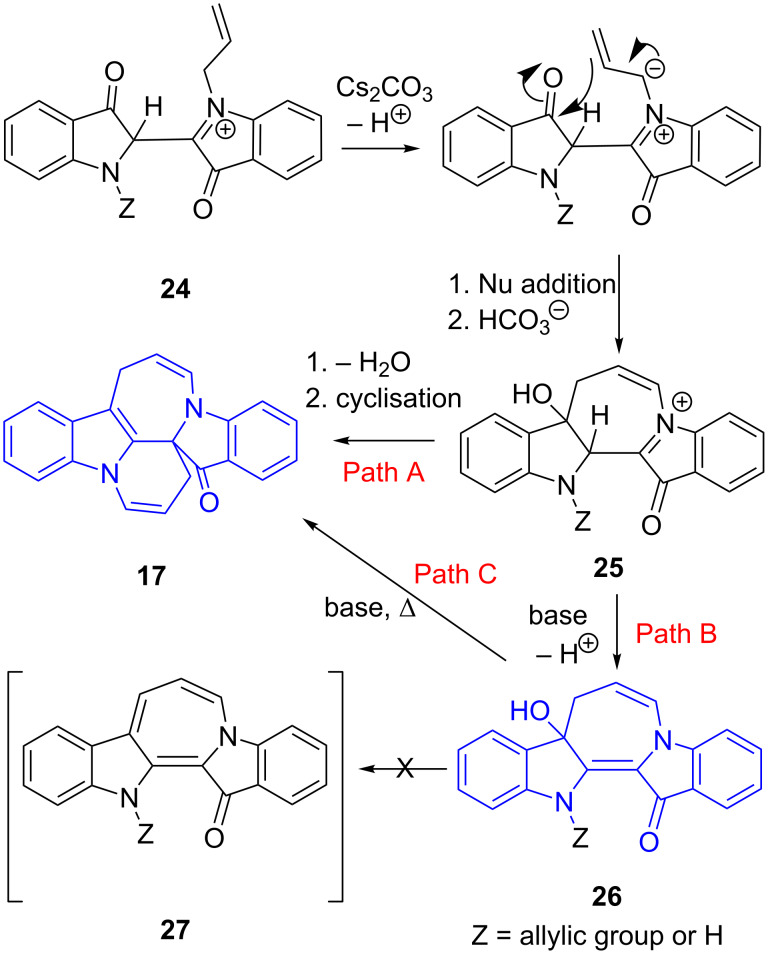
Proposed mechanism for the synthesis of the red diindolone heterocycles and the azepinodiindolones.

Scheme 6 illustrates a tentative proposed mechanism for the synthesis of the azepinodiindolones and the red diindolone heterocycles through an intermediatory diallylindigo **24** (Scheme 6). Deprotonation could then produce a stabilised ylide, thus providing a formal negative charge for subsequent cyclisation onto the carbonyl forming the 7-membered ring. In Path A, dehydration and cyclisation onto the activated iminium cation **25** could form **17**. Path B describes the base-induced deprotonation to form the neutral product **26**.

While dehydration of **26** could conceivably also occur (Scheme 6), no evidence for the expected azepine product **27** was seen. An attempt to separately dehydrate **26**, by reaction with P_2_O_5_ resulted in an inseparable mixture. However, the same compound under basic conditions (Cs_2_CO_3_) in DMF at 85–87 °C (20 min) produced **17** in 89% yield, thereby providing evidence that compounds **7**–**11** (**26** when Z = allyl) and analogues could also be intermediates in the synthesis of the azepinodiindolones as proposed in Scheme 6 (Path C).

Additionally, in support of the pathways proposed, the reaction of *N*-allylindigo **2** under the typical cascade reaction conditions for these compounds gave **17** in 59% yield and the spirocycle **12** in 8% yield.

**Scheme 7:**
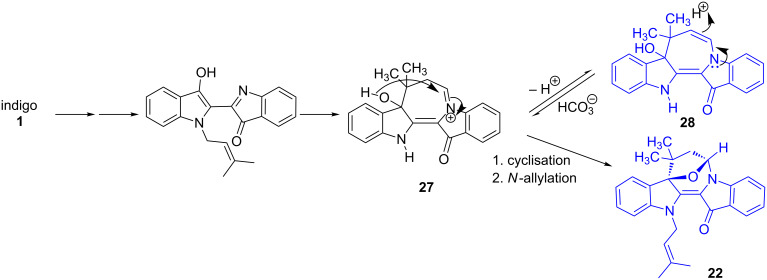
Proposed mechanism for the formation of **22**. The order of protonation and allylation is undetermined.

In the case of the dimethyl allyl analogue **22**, reversible protonation (HCO_3_^−^) of the enamine moiety in **27** (Scheme 7) and subsequent intramolecular cyclisation would realise the bridged heterocycle **22**, with this latter reaction promoted by the *gem*-dimethyl effect.

**Scheme 8:**
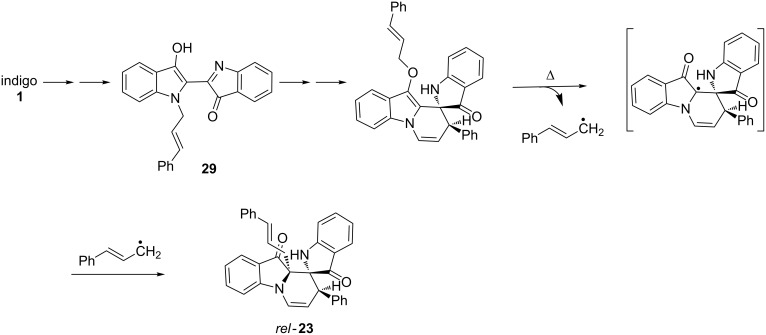
Proposed mechanism for the formation of **23** from intermediate **29**.

In the case of the spiro products, an exception is the spirocycle **23** derived from cinnamyl bromide reaction with the intermediate **29** (see Scheme 8). At the *O*-cinnamyl stage, the formation of two highly stabilised radicals could provide a driving force for a 1,3-shift leading to the C-allylated structure via a thermally induced homolytic cleavage of the cinnamyl unit (Scheme 8). While direct anionic C-allylation of *N*-cinnamylindigo may also lead to **22**, steric considerations and the non-observance of this product with other allylic bromides points to a later intervention of a 1,3-rearrangement process.

The *N*-allylisatins observed [4] are likely to be formed by oxidative cleavage processes as previously reported [4,11].

**Scheme 9:**
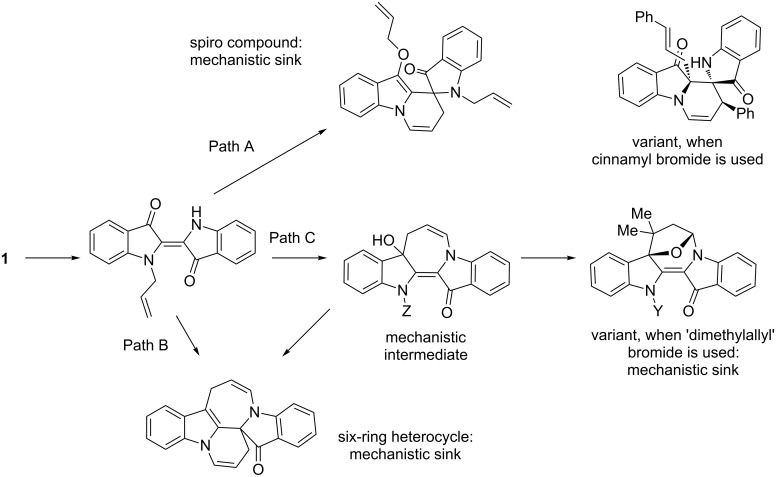
Proposed key branch points and mechanistic sinks in the base-catalysed cascade allylation reactions of indigo (**1**). Only parent heterocyclic systems are drawn except when specific substitution patterns produce particular outcomes.

Control of the branch points in the overall base-catalysed cascade allylation reactions with indigo is clearly important in achieving practical synthetic outcomes (Scheme 9). The first of these are the mono-*N*-allyindigos **2**–**6** which can branch to the synthesis of the spiro heterocycles **12**–**16** (Path A – which is a mechanistic sink). Alternatively, mono-*N*-allyindigos can progress to the synthesis of the azepinodiindolones **17** and **18** (Path B – also a mechanistic sink). However, the red diindolone heterocycles **7**–**10**, accessed from the mono-*N*-allylindigos, could be mechanistic intermediates and be transformed further into the six-ring azepinodiindolones **17** and **18** or cyclised to form the bridged compound **22** (an additional termination point – Path C).

There remains the possibility for variations outside these complex sequences, for example the radical-induced 1,3-shift of a cinnamyl moiety, likely to occur at a point buried within the mechanistic mix illustrated in Scheme 9. Clearly, further mechanistic studies will be required to continue to explain the outcomes of these cascade reactions, and therefore inform ways to increase regioselective outcomes.

### Post-allylation reactions

**Scheme 10:**
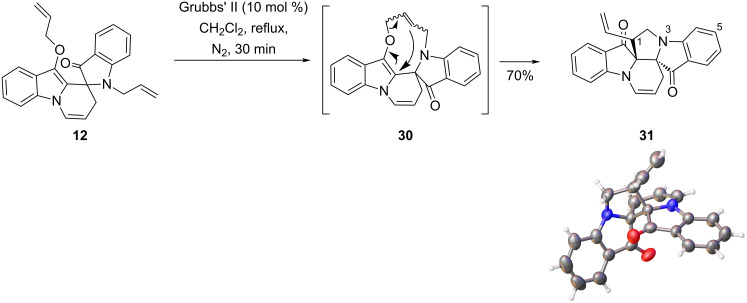
Reaction of the spiro heterocycle **12** with Grubbs' II catalyst, and X-ray crystal structure of the novel product **31** (ball and stick representation). ORTEP data for **31** is given in Supporting Information File 1.

The products obtained from the indigo allylation reactions provide potential further opportunities to access other heterocyclic structural space. For example, the spiro heterocycle **12**, with its pendant allyl substituents, affords an ideal substrate for a ring-closing metathesis reaction. In the event, treatment of **12** with Grubbs' II catalyst at reflux in CH_2_Cl_2_ produced not the expected 9-membered ring, but rather the novel fused heterocycle **31** in 70% isolated yield (Scheme 10). The structure, including relative stereochemistry, was confirmed by X-ray crystallographic analysis. This compound was formed presumably after initial 9-membered ring production to give **30** in a typical ring-closing metathesis reaction, followed by an intramolecular Claisen rearrangement. Attempts to induce a similar Claisen rearrangement starting from the original spiro compound **12** by heating a DMF solution from 40 °C to 110 °C failed, with only decomposition being observed at the higher temperatures. This suggests that the Claisen rearrangement is being catalysed by the Ru present from the Grubbs' reagent. This is not unexpected as there are reports of ruthenium-based species catalysing Claisen rearrangements [7] including a similar RCM–Claisen sequence in 2,2'-bis(allyloxy)-1,1'-binaphthyls and *O*,*O'-*(but-2-en-1,4-diyl) binaphthols [12–13]. Additionally, examples of C2 to C3 Claisen rearrangement of 2-allyloxyindoles by related Pd catalysis have been reported [14]. The ^13^C NMR spectrum of **31** showed only one set of peaks for the molecule indicating that only one pair of enantiomers was present, despite the presence of three stereogenic atoms. This is likely to arise from the spiro starting material being racemic, and the subsequent Claisen rearrangement being stereospecific.

A peak at *m*/*z* 354 [M^+^] in the EIMS corresponds to the molecular ion of the ring-closing metathesis product after elimination of an ethylene unit. Analysis of the ^1^H NMR revealed the presence of a one-proton multiplet at 3.55–3.58 ppm corresponding to H1, while gCOSY was consistent with the presence of a vinyl substituent group. HSQC spectral evidence supported three CH_2_ groups, while the ^13^C NMR spectrum confirmed the presence of two free carbonyls with signals at 197.9 and 201.0 ppm. From the HMBC spectrum, a strong correlation between C8a and H2 and also between H2 and C17a through three bonds was observed, confirming the formation of the pyrrolizidine ring system. The proposed structure of **31** was also confirmed by X-ray crystallography analysis.

### Biological activities

In preliminary screenings for biological activity, selected compounds were assessed for in vitro antiplasmodial (*Plasmodium falciparum*; drug resistant K1 strain) activity [15], cell-based anticancer activity (cell lines: NCI-H187 small cell lung cancer, KB oral cavity cancer, and MCF-7 breast cancer) [16], and in vitro antitubercular activity (*Mycobacterium tuberculosis*) [17] (Table 2).

**Table 2:** In vitro antiplasmodial, anticancer, and antitubercular activity of compounds **12**–**14**, **17**, **18**, **32–35**, **8**, **9**, and **31**.^a^

compound	IC_50_ µg/mL (µM) or MIC µg/mL (antitubercular)				
	NCI	KB	MCF-7	antitubercular	antiplasmodial

**12**	9.45 (24.7)	–	–	50.0 (131)	2.65 (6.25)
**13**	nt^b^	–	–	nt	nt
**14**	10.8 (25.5)	11.3 (26.7)	–	nt	–
**17**	3.35 (10.3)	9.71 (30.0)	–	12.5 (38.6)	3.06 (9.44)
**18**	nt	nt	–	nt	nt
**32**	5.45 (29.1)	–	–	nt	–
**33**	3.23 (16.1)	22.4 (96.4)	–		–
**34**	4.83 (24.0)	–	–	50.0 (248.5)	–
**35**	–	–	–	nt	–
**8**	13.1 (35.3)	15.0 (40.6)	5.79 (15.6)	–	1.94 (5.20)
**9**	–	24.9 (62.3)	34.7 (93.7)	–	2.97 (8.31)
**31**	–	–	–	–	–
Ellipticine	1.47	0.737			
Doxorubicin	0.077	0.504	7.97		
Tamoxifen			5.83		
Rifampicin				0.025	
Streptomycin				0.625	
Mefloquine					0.0115
Artenimol					0.00074

^a^–, not active up to 50 µg/mL; ^b^nt, not tested. NCI H187 = NCI small cell lung carcinoma, KB = KB oral cavity cancer, MCF-7 = MCF-7 breast cancer; *N*-allylisatin (**32**), *N*-2-methylpropenylisatin (**33**), *N*-crotylisatin, (**34**), *N*-3-methyl-2-butenylisatin (**35**) [4].

Promising antiplasmodial activity was seen with the spirocyclic compound **12** (IC_50_ 2.65 µg/mL, 6.25 µM) and the related indoloazepinoindol-17-one **17** in which the allylic substituents are effectively merged (minus the ether oxygen) and embedded in the 7-membered ring (IC_50_ 3.06 µg/mL, 9.44 µM). While the introduction of a methyl substituent in the allylic moiety (spiro heterocycle **14**) was detrimental to this activity, other possibilities exist for substituent variation at other sites, e.g., in the aromatic rings (starting with substituted indigo derivatives) and at the carbonyl group, for future SAR studies. In addition, with compounds of the non-spirocyclic type **8** and **9** good antiplasmodial activity was also seen, with **8** being the most potent (IC_50_ 1.94 µg/mL, 5.20 µM) of the compounds tested here.

In order to further assess selective cytotoxicity, the toxicity of **9** towards Vero cells [18] was attempted but its autofluorescence precluded a result being obtained. These spiro heterocycles, indoloazepinoindol-17-one and azepinodiindolo compounds constitute new antiplasmodial structural leads with potentially new modes of action.

Modest cytotoxicity against all three cancer cell lines tested [18] was observed with the azepinodiindolo compound **8** (IC_50_ 5.79–15.0 µg/mL, 15.6–40.6 µM). Similarly the indoloazepinoindol-17-one derivative **17** showed some activity against the small cell lung and oral cavity cancer cell lines (IC_50_ 3.35 and 9.71 µg/mL, 10.3 and 30.0 µM, respectively), but not against the more refractory MCF-7 breast cancer cell line [16]. Additionally, **17** exhibited some antitubercular activity (MIC 12.5 µg/mL, 38.6 µM), providing a basis for further structurally novel lead development of anti-TB compounds. The need for such compounds is a pressing one with the development of major mycobacterial resistance [19].

Patchy cytotoxic activity was seen with the N-substituted isatins **32**, **33**, and **34**. Compounds of this general type, but incorporating *N*-arylmethyl as well as 5,7-dibromo substituents, have given rise to potent anticancer compounds with activity probably being mediated, at least in some cases, through microtubule destabilisation and inhibition of tubulin polymerisation [20–22].

## Conclusion

The search for diversity in new heterocyclic chemical space is increasingly important in areas such as medicinal chemistry and nanotechnology, where novel heterocyclic starting points are urgently required. The newly established cascade allylation chemistry of indigo provides a fertile ground for the discovery of such heterocycles not readily available by other means, and key to this is the ability to produce novel heterocyclic structures in one-pot in reasonable yield. We report here the optimisation of the synthesis of two heterocycles, the spiro compound **14** (65%) and the fused 7-membered ring product **17** (72%) – both these heterocycles are synthesised in one pot from a cheap and readily available starting material and represent an exceptionally efficient synthesis of novel polycyclic compounds. Further, we report for the first time the synthesis of derivatives of these heterocycles, including those using the sterically hindered allyl reagents with terminal methyl substituents.

The allylation reaction also provides access to new hydroxylated heterocyclic derivatives of the azepinodiindolo type. These intensely red compounds can be synthesised in one pot in yields of up to 51% and are presumably also intermediates in the synthesis of the indoloazepinoindol-17-ones. One of the more interesting outcomes is the first synthesis of the bridged compound **22**, with a heterocyclic skeleton not likely to be readily accessible by other means. As with all these reactions, the synthesis of **22** is repeatable, and given the reliability of outcome and complexity of this structure, a 26% yield is a reasonable achievement.

Enhancement of the synthetic scope of the tandem RCM–Claisen chemistry has been established with the production of the new heterocyclic system **31** from the spiro compound **12**. Further application of this tandem methodology offers significant potential in heterocyclic synthesis.

Promising in vitro antiplasmodial activity was indicated with a number of the spiro, indoloazepinoindol-17-one and azepinodiindolo heterocycles, while the in vitro antitubercular activity of one indoloazepinoindol-17-one compound, **17**, was also of interest.

We have reported here significant new chemistry in the cascade reactions of indigo. This recently discovered area has the potential through other substituted alkylating agents and through substituted indigos and variations on that skeleton, to greatly contribute to the synthesis of a diverse array of new heterocyclic compounds. Important in this area is a deeper understanding of the mechanisms involved and strategies for the optimisation of regio- and stereospecific product yields in these one-pot reactions and further studies are continuing.

## Supporting Information

File 1 Experimental procedures, UV–vis spectra, copies of the ^1^H and ^13^C NMR spectra for the new compounds and ORTEP plots for the reported compounds including supplementary pictures.

## References

[1] Dančík V, Seiler K P, Young D W, Schreiber S L, Clemons P A (2010). J Am Chem Soc.

[2] Kaiser M, Wetzel S, Kumar K, Waldmann H (2008). Cell Mol Life Sci.

[3] Shakoori A, Bremner J B, Willis A C, Haritakun R, Keller P A (2013). J Org Chem.

[4] Abdel-Hamid M K, Bremner J B, Coates J, Keller P A, Miländer C, Torkamani Y S, Skelton B W, White A H, Willis A C (2009). Tetrahedron Lett.

[5] Holst P B, Anthoni U, Christophersen C, Larsen S, Nielsen P H, Püschl A (1998). Acta Chem Scand.

[6] Mo D-L, Wink D J, Anderson L L (2014). Chem – Eur J.

[7] Le Nôtre J, Touzani R, Lavastre O, Bruneau C, Dixneuf P H (2005). Adv Synth Catal.

[8] Ramig K (2013). Tetrahedron.

[9] Beesley R M, Ingold C K, Thorpe J F (1915). J Chem Soc, Trans.

[10] Jung M E, Piizzi G (2005). Chem Rev.

[11] Gandra N, Frank A T, Le Gendre O, Sawwan N, Aebisher D, Liebman J F, Houk K N, Greer A, Gao R (2006). Tetrahedron.

[12] Piedra E, Francos J, Nebra N, Suárez F J, Díez J, Cadierno V (2011). Chem Commun.

[13] Abraham M, Reischl W, Kirchner K A, Roller A, Veiros L F, Widhalm M (2012). Molecules.

[14] Linton E C, Kozlowski M C (2008). J Am Chem Soc.

[15] Desjardins R E, Canfield C J, Haynes J D, Chulay J D (1979). Antimicrob Agents Chemother.

[16] Mahéo K, Vibet S, Steghens J P, Dartigeas C, Lehman M, Bougnoux P, Goré J (2005). Free Radical Biol Med.

[17] Changsen C, Franzblau S G, Palittapongarnpim P (2003). Antimicrob Agents Chemother.

[18] O'Brien J, Wilson I, Orton T, Pognan F (2000). Eur J Biochem.

[19] Müller B, Borrell S, Rose G, Gagneux S (2013). Trends Genet.

[20] Vine K L, Matesic L, Locke J M, Ranson M, Skropeta D (2009). Anti-Cancer Agents Med Chem.

[21] Vine K L, Locke J M, Ranson M, Pyne S G, Bremner J B (2007). J Med Chem.

[22] Matesic L, Locke J M, Bremner J B, Pyne S G, Skropeta D, Ranson M, Vine K L (2008). Bioorg Med Chem.

