# Supplementary material for: Further exploration of the heterocyclic diversity accessible from the allylation chemistry of indigo
Source: Beilstein J Org Chem. 2015 Apr 15;11:481–92. doi: 10.3762/bjoc.11.54 (PMC4419534; doi:10.3762/bjoc.11.54)
Supplement: File 1 — Experimental procedures, UV–vis spectra, copies of the 1H and 13C NMR spectra for the new compounds and ORTEP plots for the reported compounds including supplementary pictures. [file Beilstein_J_Org_Chem-11-481-s001.pdf]

Supporting Information

for

Further exploration of the heterocyclic diversity accessible from the allylation chemistry of indigo

Alireza Shakoori[1], John B. Bremner[1], Mohammed K. Abdel-Hamid[1], Anthony C. Willis[2], Rachada Haritakun[3] and Paul A. Keller*[1]

Address: [1]School of Chemistry, University of Wollongong, Wollongong, NSW, 2522, Australia, [2]School of Chemistry, The Australian National University, Canberra, ACT 0200, Australia and [3]National Centre for Genetic Engineering and Biotechnology (BIOTEC), National Science and Technology Development Agency (NSTDA), 113 Phaholyothin Road, Klong1, Klong Luang, Pathumanthani 12120 Thailand

Email: Paul A Keller* - keller@uow.edu.au

*Corresponding author

Experimental procedures, UV–vis spectra, copies of the ^{1}H and ^{13}C NMR spectra for the new compounds and ORTEP plots for the reported compounds including supplementary pictures

Table of contents

Example systematic names of compounds reported S2

General information S3

Experimental procedures and analytical data S4

NMR Spectra of the new compounds S23

N-Monoallylated compounds from the 5 s reaction S23

Azepinodiindolones from the 1 h reaction S28

Spiro compound from the 3 h reaction S36

Oxa-bridged azepinodiindolone S40

Ring-closing metathesis product S48

UV–vis spectra of the new compounds S52

Crystallographic studies S58

Crystallographic data for compounds **2**, **7**, **8**, **9**, **10**, **23** and **31** S58

ORTEP plots of the reported compounds along with their Cambridge Crystallographic Data Centre code S59

Supplementary photos S63

References S64

Example systematic names of compounds reported

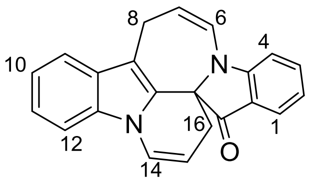

8*H*,16*H*-Pyrido[1,2,3-*s,t*]indolo[1,2-*a*]azepino[3,4-*b*]indol-17-one

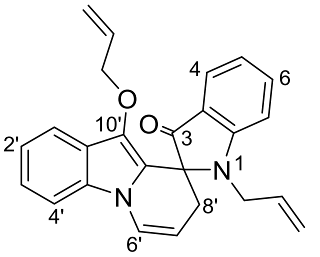

1-Allyl-10'-(allyloxy)-2'*H*-spiro(indoline-2,9'-pyrido[1,2-*a*]indol)-3-one

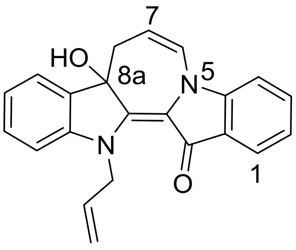

13-Allyl-8a-hydroxy-8a,13-dihydroazepino[1,2-*a*:3,4-*b*′]diindol-14(8*H*)-one

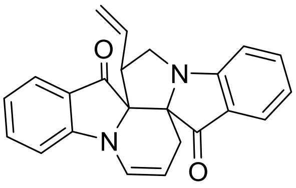

1,2-Dihydro-1-vinyl-8*H*,9*H*,17*H*-benz[2′,3′]pyrrolizino[1′,7′a:2,3]pyrido[1,2-*a*]indole-8,17-dione

General Information

Reagents and solvents were purchased reagent grade and used without further purification unless otherwise stated. All reactions were performed in standard oven-dried glassware under a nitrogen atmosphere unless otherwise stated. Melting points measured on a Büchi Melting Point M-560 apparatus are expressed in degrees Celsius (°C) and are uncorrected. ^{1}H and ^{13}C NMR spectra were all run in $CDCl_3$ solutions (unless otherwise stated) at 25 °C and were recorded at 500 and 125 MHz respectively on a Varian Inova 500 MHz spectrometer, with chemical shifts (δ) reported in parts per million relative to TMS (δ = 0 ppm) or $CDCl_3$ (δ = 77.0 ppm) as internal standards. Coupling constants (*J*) are reported in Hertz (Hz). Multiplicities are reported as singlet (s), broad singlet (bs), doublet of doublets (dd), or multiplet (m). Electron impact (EI) mass spectra (MS) were recorded on a Shimadzu QP-5000 spectrometer and high resolution (HR) on a VG AutoSpec spectrometer. Electrospray (ESI single quadrupole) mass spectra were recorded on a Micromass Platform LCZ spectrometer and high resolution spectra on a Micromass QTOF2 spectrometer. Ion mass to charge (*m*/*z*) values are stated with their relative abundances as a percentage in parentheses. Peaks assigned to the molecular ion are denoted by M^+ or $[M + H]^+$. Infrared (IR) spectra were recorded on KBr diluted samples by the Diffuse Reflection Method on a Shimadzu IR Affinity 1 instrument. UV–vis spectra were recorded on a Cary 100 Bio UV–vis spectrophotometer with solutions of the samples in CH_2Cl_2. Images from crystals were captured using a Leica MZ 16 A stereo microscope. All the images were obtained from X-ray quality single crystals. Optical rotations were measured on a Jasco p-2000 polarimeter in CH_2Cl_2 solution at 25 °C. Thin layer chromatography (TLC) and preparative thin layer chromatography (PTLC) was performed using silica gel F254 aluminum sheets (TLC) or on glass plates (PTLC). Column chromatography was performed under gravity using Merck silica gel 60 (0.063–

0.200 mm). Eluents are in volume to volume (v:v) proportions. Solvent extracts or chromatographic fractions were concentrated by rotary evaporation in vacuo. Indigo (dye content 95%) was used without further purification. Petroleum spirit (Pet. spirit) had a bp range of 40–60 °C. Calculations were performed using Wavefunction Spartan '10 v1.1.0 with geometry optimisations at the semiempirical AM1 level.

X-ray Structure Determination - images were measured on a Nonius Kappa CCD diffractometer (MoKα, graphite monochromator, λ = 0.71073 Å) and data extracted using the DENZO package [1]. Structure solution was by direct methods (SIR92) [2]. The structures were refined using the CRYSTALS program package [3]. Atomic coordinates, bond lengths and angles, and displacement parameters for compounds **2**, **7**, **8**, **9**, **10**, **23** and **31** have been deposited at the Cambridge Crystallographic Data Centre (CCDC nos. 986247 – 986253, respectively). These data can be obtained free-of-charge via www.ccdc.cam.ac.uk/data_request/cif, by emailing data_request@ccdc.cam.ac.uk, or by contacting The Cambridge Crystallographic Data Centre, 12 Union Road, Cambridge CB2 1EZ, UK; fax: +44 1223 336033.

Synthetic Note: All potentially chiral compounds in this work were isolated or used as racemates.

Reaction of indigo with allyl bromide

Method A: 5 s reaction

(*E*)-1-Allyl-[2,2'-biindolinylidene]-3,3'-dione (2) and 13-allyl-8a-hydroxy-8,13a-dihydroazepino[1,2-*a*:3,4-*b'*]diindol-14(8*H*)-one (7)

A suspension of indigo (262 mg 1.00 mmol) in anhydrous DMF (40 mL) was sonicated for 30 min and the resulting suspension was transferred to a septum-equipped round bottom flask carrying pre-dried Cs_2CO_3 (1.303 g, 4.00 mmol) under N_2 flow. The flask was plunged into a preheated oil bath at 85–88 °C and stirred for 30 min. The N_2 flow was stopped and allyl bromide (605 mg, 5.00 mmol) was added rapidly in one portion by syringe, and after 5 s, the reaction mixture was poured into an ice bath. The blue-black precipitate was filtered and dissolved in hot CH_2Cl_2 and cooled at 5 °C overnight. The mixture was then filtered to remove the unreacted indigo (36%). The filtrate was concentrated under reduced pressure then recrystallised from Pet. spirit/EtOAc (90:10) to furnish **2** (163 mg, 54%) as a dark navy crystalline solid; R_f = 0.68 (CH_2Cl_2/Pet. spirit; 7:3), m.p: 170-171 ºC. X-ray quality crystals were obtained by slow recrystallisation from $CHCl_3$ in a small tube while it was placed in another sample tube containing Pet. spirit (10 mL). UV–vis (CH_2Cl_2) λ_{max}/nm (ε, $M^{-1}cm^{-1}$) 289 (22571), 614 (11924). IR (neat) $υ_{max}$ 3290 (m), 1635 (s), 1608 (s), 1462 (s), 1384 (m), 1064 (m), 1027 (s), 921 (m), 748 (m) cm^{-1}. ^{1}H NMR δ = 5.09-5.16 (m, 3H, H2'', H3''a, H3''b), 5.90-5.94 (s, 2H, H1''), 6.92 (t, *J* = 7.5 Hz, 1H, H5'), 6.97 (d, *J* = 8.3 Hz, 1H, H7), 7.00 (t, *J* = 7.5 Hz, 1H, H5), 7.07 (d, *J* = 8.3 Hz, 1H, H7'), 7.44 (t, *J* = 7.9 Hz, 1H, H6'), 7.50 (t, *J* = 7.9 Hz, 1H, H6), 7.65 (d, *J* = 7.5 Hz, 1H, H4), 7.73 (d, *J* = 7.5 Hz, 1H, H4'), 10.71 (s, 1H, NH). ^{13}C NMR δ = 49.7 (C1''), 111.0 (C7), 111.8 (C5'), 116.8 (C3''), 120.2 (C3'a), 120.6 (C7'), 120.7 (C5), 120.9 (C3a), 122.8 (C2), 124.0 (C4'), 124.7 (C4), 125.6 (C2'), 133.1 (C2''), 135.7 (C6), 136.0 (C6'), 151.4 (C7a), 152.7 (C7'a), 187.1 (C3'), 189.7 (C3). MS (EI): *m/z* = 302 (66%, M^+), 273 (27), 233 (24), 181 (100). HRMS (ESI): calcd for $C_{19}H_{14}N_2O_2$ $[M+H]^+$ 303.1134; found 303.1143.

The mother liquor from the recrystallization above was then dried (Na_2SO_4), concentrated and subjected to PTLC and developed with CH_2Cl_2/EtOAc; 9.5:0.5.

The selected band was collected and soaked in EtOAc and filtered. The filtrate was concentrated to afford **7** (30.8 mg, 9%) as a red solid; mp. 163-165 ºC. R_f = 0.20 (CH_2Cl_2/EtOAc; 9.5:0.5). UV-Vis (CH_2Cl_2) λ_{max}/nm (ε, $M^{-1}cm^{-1}$) 306 (14496), 363 (16077), 370 (10096), 498 (11840). IR (neat) υ_{max} 3275 (b), 1608 (m), 1535 (s), 1465 (s), 1419 (m), 1319 (m), 1180 (m), 1111 (m), 748 (s) cm^{-1}. ^1H NMR δ = 2.46 (d, J = 17.1 Hz, 1H, H8a), 2.97 (dd, J = 12.2, 17.3 Hz, 1H, H8b), 4.57 (d, J = 15.3 Hz, 1H, 1'a), 4.73 (s, 1H, O<u>H</u>), 4.94-5.03 (m, 3H, H1'b, H7, H3'a), 5.14 (d, J = 17.3 Hz, 1H, H3'b), 5.80-5.85 (m, 1H, H2'), 6.85 (d, J = 9.8 Hz, 1H, H6), 6.90 (t, J = 7.5 Hz, 1H, H2), 6.98 (d, J = 8.0 Hz, 1H, H4), 7.00 (d, 1H, J = 8.2 Hz, H12), 7.11 (t, J = 7.5 Hz, 1H, H10), 7.32 (t, J = 7.5 Hz, 1H, H3), 7.49 (d, J = 7.2 Hz, 1H, H9), 7.61 (d, J = 7.6 Hz, 1H, H11). ^{13}C NMR δ = 38.9 (C8), 52.6 (C1'), 81.3 (C8a), 101.9 (C7), 109.4 (C4), 111.1 (C12), 115.7 (C13b), 117.7 (C3'), 120.2 (C6), 122.6 (C14a), 123.2 (C12), 123.6 (C10), 123.7 (C9), 124.2 (C1), 129.7 (C2'), 133.0 (C11), 134.5 (C3), 136.0 (C8b), 145.1 (C13a), 147.6 (C12a), 156.0 (C4a), 178.5 (C14). MS (EI): m/z = 342 (91%, M^+), 324 (100). HRMS (ESI): calcd. for $C_{22}H_{18}N_2O_2$ $[M+H]^+$ 343.1447; found 343.1440.

Reaction of indigo with allyl bromide

Method B: 1 h reaction

13-Allyl-8a-hydroxy-8,13a-dihydroazepino[1,2-*a*:3,4-*b*']diindol-14(8*H*)-one (7)

1-allyl-10'-(allyloxy)-8'*H*-spiro[indoline-2,9'-pyrido[1,2-*a*]indol]-3-one (12)

A mixture of indigo (262 mg, 1.00 mmol) in anhydrous DMF (40.0 mL) was sonicated for 30 min and the resulting suspension was dripped into a flask containing 4 Å molecular sieves and pre-dried anhydrous Cs_2CO_3 (1.303 g, 4.00 mmol). The flask was plunged into a pre-heated oil bath at 85–88 °C and the mixture stirred for 30 min under a N_2 flow. The flow was then cut and allyl bromide (600 mg, 5.00 mmol) was syringed in and the mixture was stirred and heated at

85–88 °C for 1 h under a static inert atmosphere (N_2). The crude mixture was then filtered hot (to remove the molecular sieves) into an ice bath. The aqueous mixture was transferred to a conical flask and partitioned in CH_2Cl_2 and washed with brine (2 × 30 mL) and water (5 × 50 mL). The combined organic layers were concentrated under reduced pressure. The residue from the ice bath (thick oily red-orange drops) was dissolved in CH_2Cl_2. The solution was dried over Na_2SO_4 and combined with the collected organic phase from extraction of the aqueous mixture from the ice bath. The filtrate was then concentrated under reduced pressure and adsorbed onto silica (1:1 CH_2Cl_2 : Pet. spirit). The silica was then washed and filtered through a sinter with CH_2Cl_2/Pet. spirit (9:1) until the filtrate became colourless (filtrate A). The silica was then soaked in EtOAc and filtered. The filtrate was concentrated and the red powder was recrystallized from CH_2Cl_2 giving **7** (140 mg, 41%) as red crystalline flakes.

Filtrate A was concentrated under reduced pressure and subjected to silica gel column chromatography. Elution with 7:3 CH_2Cl_2/Pet. spirit resulted in isolation of compound **2** (27.2 mg, 9%) and compound **12** (11.5 mg, 3%).

Reaction of indigo with allyl bromide

Method C: 3 h reaction

8*H*,16*H*-Pyrido[1,2,3-*s*,*t*]-indolo[1,2-*a*]azepino[3,4*b*]indol-17-one (17)

1-allyl-10'-allyloxy-2'*H*-spiro(indoline-2,1'-pyrido[1,2-*a*]indol)-3-one (12)

A suspension of indigo (262 mg, 1.00 mmol) in DMF was sonicated for 30 min at room temperature and transferred to a septum equipped round bottom flask containing pre-dried and grinded Cs_2CO_3 (1.303 g, 4.00 mmol) and molecular sieves under an inert atmosphere. The flask was plunged to a pre-heated oil bath at 85–88 °C and stirred for 30 min. The inert atmosphere flow was then cut and

allyl bromide (600 mg, 5.00 mmol) was added, and the mixture was stirred and heated for 1 h under a static inert atmosphere (N_2). Another portion of allyl bromide (2.00 mmol) was added to the mixture and it was stirred for a further 2 h. The colour of solution turned brown-yellow and TLC analysis indicated the complete consumption of indigo. Molecular sieves were filtered from the hot reaction mixture and the filtrate poured into an ice bath and then partitioned in water and CH_2Cl_2 and washed with brine (2 × 30 mL) and water (5 × 50 mL) and the combined organic phases were dried (Na_2SO_4) and concentrated under reduced pressure. The residue was subjected to flash silica gel column chromatography and elution with Pet. spirit/CH_2Cl_2 (1:3) yielded **17** (221 mg, 72%) as a yellow-brown powder; mp 143-144 °C, *R*f = 0.61 (CH_2Cl_2/Pet. spirit; 7:3). X-ray quality crystals were grown through slow crystallisation from Pet. spirit/CH_2Cl_2 (5:3). Results from spectral analysis of the purified compound were compatible with the reported literature values [4]. Further elution of the SiO_2 flash column using CH_2Cl_2/Pet. spirit (3:1) yielded a fraction that was further subjected to gravity column chromatography (Pet. spirit/ethyl acetate, 3:1) followed by PTLC (Pet. spirit/ethyl acetate, 3:2) to yield two products; one was 1-allyl-10'-allyloxy-2'*H*-spiro(indoline-2,1'-pyrido[1,2-*a*]indol)-3-one **12** (52.3 mg, 15%) as an orange yellow solid; mp. 121-122 ºC, R_f = 0.51 (CH_2Cl_2/Pet. spirit; 7:3).

Reaction of indigo with 3-bromo-2-methylpropene

5 s reaction

(*E*)-1-(2-Methylallyl)-[2,2'-biindolinylidene]-3,3'-dione (3)

8a-hydroxy-7-methyl-13-(2-methylallyl)-8,13a-dihydroazepino[1,2-*a*:3,4-*b*']diindol-14(8*H*)-one (8)

Using Method A, indigo (262 mg, 1.00 mmol) and 3-bromo-2-methylpropene (670 mg, 5.00 mmol) was reacted at 85–88 °C. The reaction mixture was poured onto

an ice bath and the dark navy or black precipitate was collected and dissolved in hot CH_2Cl_2 and cooled at 5 °C overnight. The mixture was then filtered to remove the unreacted indigo (29%). The filtrate was concentrated under reduced pressure then dissolved in hot MeOH/H_2O (1:3) then cooled to afford **3** (196 mg, 62%) as a dark navy powder mp. 150-152 °C, R_f = 0.72 (CH_2Cl_2/Pet. spirit; 7:3). UV−vis (CH_2Cl_2) λ_{max}/nm (ε, $M^{-1}cm^{-1}$) 291 (21876), 628 (11669). IR υ_{max} 3271 (m), 1608 (s), 1462 (s), 1388 (m), 1296 (s), 1172 (m), 1072 (s) 1037 (s), 918 (w), 748 (m), 698 (m) cm^{-1}. ^{1}H NMR δ = 1.76 (s, 3H, H1'''), 4.62 (s, 1H, H3''a), 4.83 (d, *J* = 6.9 Hz, 1H, H3''b), 5.11 (s, 2H, H1''), 6.93 (t, *J* = 7.3 Hz, 1H, H5'), 6.97-7.04 (m, 3H, H7, H7', H5), 7.45 (t, *J* = 7.7 Hz, 1H, H6'), 7.51 (t, *J* = 7.7 Hz, 1H, H6), 7.65 (d, *J* = 7.7 Hz, 1H, H4), 7.58 (d, *J* = 7.3 Hz, 1H, H4'), 10.60 (s, 1H, NH). ^{13}C NMR ($(CD_3)_2SO$) δ = 20.5 (C1'''), 52.6 (C1'), 112.1 (C3''), 112.8 (C7), 114.1 (C5'), 120.0 (C3'a), 120.9 (C3a), 121.3 (C7'), 121.8 (C5), 123.4 (C2), 124.3 (C4), 124.8 (C4'), 125.2 (C2'), 136.8 (C6), 136.9 (C6'), 141.3 (C2''), 152.8 (C7a), 153.7 (C7'a), 187.4 (C3'), 189.0 (C3). MS (EI): *m/z* = 316 (81%), 299 (22), 195 (100). HRMS (ESI): calcd for $C_{20}H_{17}N_2O_2$ $[M+H]^+$ 317.1290; found 371.1298.

The mother liquor was dried (Na_2SO_4), concentrated and then subjected to PTLC and developed with CH_2Cl_2/EtOAc; 9.5:0.5. The selected band was collected and soaked in EtOAc and filtered. The filtrate was concentrated to afford **8** (25.9 mg, 7%) as a red powder; mp. 172-174 ºC. R_f = 0.19 ($CH_2Cl_/$EtOAc; 9.5:0.5). UV−vis (CH_2Cl_2) λ_{max}/nm (ε, $M^{-1}cm^{-1}$) 306 (14007), 377 (10809), 511 (10483). IR υ_{max} 3143 (b), 1651 (s), 1608 (s), 1543 (s), 1465 (s), 1377 (m), 1315 (s), 1192 (m), 1118 (s), 1087 (m), 894 (w), 740 (s) cm^{-1}. ^{1}H NMR δ = 1.58 (s, 3H, H1''), 1.95 (s, 3H, H1'''), 2.58 (d, *J* = 17.1 Hz, 1H, H8a), 2.96 (d, *J* = 15.0 Hz, 1H, H8b), 4.24 (d, *J* = 15.0 Hz, 1H, H3'a), 4.79 (d, *J* = 36.9, 2H, H1'a,b), 5.13 (bs, 1H, OH), 5.42 (d, *J* = 15.0 Hz, 1H, H3'b), 6.73 (s, 1H, H6), 6.80 (t, *J* = 13.5 Hz, 1H, H2), 6.90 (d, *J* = 8.1

Hz, 1H, H12), 7.10-7.20 (m, 2H, H4, H10), 7.30-7.40 (m, H3, 2H, H11), 7.50 (d, *J* = 7.8 Hz, 1H, H1), 7.58 (d, *J* = 7.5 Hz, 1H, H9). ^{13}C NMR δ = 19.9 (C1''), 24.8 (C1'''), 43.9 (C8), 54.1 (C3'), 80.6 (C8a), 109.8 (C4), 110.9 (C12), 111.4 (C7), 112.8 (C1'), 116.6 (C13b), 120.1 (C2), 120.9 (C6), 123.0 (C14a), 123.3 (C9,C10), 123.7 (C1), 129.6 (C11), 133.1 (C3), 135.6 (C8b), 139.5 (C2'), 144.8 (C12a), 148.3 (C4a), 154.9 (C13a), 178.4 (C14). MS (EI): *m/z* = 370 (82%, M^+), 353 (30), 315 (71), 299 (32), 195 (100). HRMS (ESI): calcd for $C_{24}H_{23}N_2O_2$ $[M+H]^+$ 371.1760; found 371.1760.

Reaction of indigo with 3-bromo-2-methylpropene

1 h reaction

8*a*-Hydroxy-7-methyl-13-(2-methylallyl)-8,13*a*-dihydroazepino[1,2-*a*:3,4-*b*']diindol-14(8*H*)-one (8)

Using Method B, indigo (262 mg, 1.00 mmol) and 3-bromo-2-methylpropene (670 mg, 5.00 mmol) was reacted at 85 ºC for 1 h. Upon workup, the filtrate was concentrated under reduced pressure and subjected to a 20 × 1.5 cm silica gel column chromatography. Elution with CH_2Cl_2 (300 mL) flushed the by-products through (compound **3**, 22.1 mg, 7% and compound **13**, 29.7 mg, 7%), before elution with CH_2Cl_2/EtOAc (95:5, 300 mL) yielded a red powder which was recrystallized from Pet. spirit/EtOAc (9:1) to yield **8** (204 mg, 51%) as shiny ruby crystals.

Reaction of indigo with 3-bromo-2-methylpropene

3 h reaction

7,15-Dimethyl-8*H*,16*H*-pyrido[1,2,3-*s,t*]-indolo[1,2-*a*]azepino[3,4-*b*]indol-17-one (18)

3'-Methyl-1-[3-(2-methyl)prop-1-enyl]-10'-[3-(2-methyl)prop-1-enyl]oxy-2'*H*-spiro(indoline-2,1'-pyrido[1,2-*a*]indol)-3-one (13)

Using Method C, indigo (262 mg, 1.00 mmol) was reacted with 3-bromo-2-methylpropene (680 mg, 5.00 mmol). Upon workup, the residue was dissolved in CH_2Cl_2 (10 mL) and Pet. spirit was added gradually while the dark brown mixture turned cloudy. The mixture was heated until it become clear. Crystallisation on cooling overnight deposited the luminescent yellow grains of **18** (183 mg, 52%) mp. 123-124 ºC, R_f = 0.58 (CH_2Cl_2/Pet. spirit; 7:3). Spectral characteristics are compatible with the literature [4].

The mother liquor from recrystallisation was concentrated and subjected to flash column chromatography. Elution with CH_2Cl_2/Pet. spirit (70:30) yielded 7,15-dimethyl-8*H*,16*H*-pyrido[1,2,3-*s,t*]-indolo[1,2-*a*]azepino[3,4-*b*]indol-17-one (**18**, 68.0 mg, 17%). Further elution afforded **13** (63.0 mg, 15%) as a yellow powder; mp. 118-120 ºC, R_f = 0.46 (CH_2Cl_2/Pet. spirit; 7:3). Spectral characteristics were compatible with the literature data [4].

Reaction of indigo with 1-bromo-2-butene

5 s reaction

(*E*)-1-((*E*)-But-2-en-1-yl)-[2,2'-biindolinylidene]-3,3'-dione (4)

(*E*)-13-(but-2-en-1-yl)-8a-hydroxy-8-methyl-8,13a-dihydroazepino[1,2-*a*:3,4-*b*']diindol-14(8*H*)one (9)

Using Method A, indigo (262 mg, 1.00 mmol) and (*E*)-1-bromobut-2-ene (670 mg, 5 mmol) were reacted at 85–88 °C for 5 s. The reaction mixture was poured into an ice bath and the dark navy or black precipitate was collected and dissolved in hot CH_2Cl_2 and cooled at 5 °C overnight. The mixture was then filtered to remove the unreacted indigo (30%). The filtrate was concentrated under reduced pressure

then dissolved in hot MeOH/H_2O (1:3) and cooled to precipitate **4** (180 mg, 57%) as a dark navy powder, mp. 161-163 ºC. R_f = 0.70 (CH_2Cl_2 : Pet. spirit; 7:3). UV–vis (CH_2Cl_2) λmax/nm (ε, $M^{-1}cm^{-1}$) 291 (28583), 632 (16946). IR $υ_{max}$ 3244 (b), 1631(s), 1608 (s), 1465 (s), 1446 (m), 1330 (s), 1180 (s), 1111 (s), 964 (m), 748 (s) cm^{-1}. ^{1}H NMR δ = 1.62 (d, *J* = 6.1 Hz, 3H, C4'-CH_3), 5.06 (d, *J* = 5.0 Hz, 2H, H1''), 5.53-5.58 (m, 1H, H2'') 5.62-5.65 (m, 1H, H3''), 6.97 (t, *J* = 7.5, Hz, 1H, H5'), 7.01-7.05 (m, 2H, H7, H5), 7.13 (d, *J* = 8.2 Hz, 1H, H7'), 7.48 (t, *J* = 7.5 Hz, 1H, H6'), 7.54 (t, *J* = 7.7 Hz, 1H, H6), 7.70 (d, *J* = 7.7 Hz, 1H, H4), 7.75 (d, *J* = 7.3 Hz, 1H, H4'), 10.57 (s, 1H, NH). ^{13}C NMR (($CD_3)_2SO$) δ = 18.3 (C4''), 49.2 (C1''), 113.0 (C7), 114.1 (C5'), 120.1 (C3'a), 120.6 (C7'), 121.7 (C3a), 121.9 (C5), 123.4 (C2), 124.3 (C4), 124.8 (C4'), 125.2 (C2'), 126.8 (C2''), 129.3 (C3''), 136.8 (C6), 136.9 (C6'), 152.8 (C7a), 153.7 (C7'a), 187.3 (C3'), 189.6 (C3). MS (EI): *m/z* = 316 (70%, M^+), 301 (52), 261 (24), 233 (72), 195 (100), HRMS (ESI): calcd. for $C_{20}H_{17}N_2O_2$ $[M+H]^+$ 317.1290; found 317.1301.

The mother liquor was dried (Na_2SO_4), concentrated and then subjected to a PTLC plate and developed with CH_2Cl_2/EtOAc; 9.5:0.5. The selected band was collected and soaked in EtOAc and filtered. The filtrate was concentrated to afford **9** as a red powder (37.0 mg, 10%); mp. 148-150 ºC, R_f = 0.15 (CH_2Cl_2/EtOAc; 9.5:0.5). UV–vis (CH_2Cl_2) $λ_{max}$/nm (ε, $M^{-1}cm^{-1}$) 306 (13821), 367 (11872), 494 (14637). IR $υ_{max}$ 3278 (m), 1643 (s), 1608 (s), 1465 (s), 1388 (m), 1296 (m), 1168 (m), 1056 (m), 1018 (s), 918 (w), 752 (m) cm^{-1}. ^{1}H NMR δ = 0.67 (d, *J* = 7.1 Hz, 3H, H1''-CH_3), 1.61 (d, *J* = 6.1 Hz, 3H, H4'), 3.07 (p, *J* = 7.1 Hz, 1H, H8), 3.85 (s, 1H, OH), 4.54 (dd, *J* = 7.7, 5.0 Hz, 1H, H1'a), 5.02 (d, *J* = 7.7 Hz, 1H, H1'b), 5.06-5.09 (m, 1H, H7), 5.54-5.59 (m, 1H, H2'), 5.67-5.74 (m, 1H, H3'), 6.87 (d, *J* = 10.2 Hz, 1H, H6), 6.93 (t, *J* = 7.6 Hz, 1H, H2), 7.00 (d, *J* = 7.6 Hz, 1H, H12), 7.10 (t, *J* = 7.1 Hz, 1H, H4) 7.14 (d, *J* = 8.1 Hz, 1H, H10), 7.30-7.40 (t, *J* = 7.7 Hz, 1H, H3),

7.38 (m, 2H, H1, H11), 7.62 (d, J = 7.5 Hz, 1H, H9). ^{13}C NMR δ = 17.6 (C1''), 18.7 (C4'), 43.9 (C8), 51.7 (C1'), 84.3 (C8a), 109.4 (C4), 110.0 (C7), 110.1 (C12), 117.4 (C13b), 120.1 (C2), 122.1 (C6) 122.2 (C14a), 122.6 (C9), 123.0 (C10), 123.7 (C1), 123.8 (C13a), 126.3 (C2'), 129.8 (C11), 130.2 (C3'), 133.0 (C3), 133.3 (C8b), 146.1 (C12a), 152.3 (C4a), 178.7 (C14). MS (EI): *m/z* = 370 (48%, M$^+$), 327 (100), 315 (47), 299 (78), 285 (75), 272 (68). HRMS (ESI): calcd for $C_{24}H_{23}N_2O_2$ [M+H]$^+$ 371.1760; found 371.1747.

Reaction of indigo with 1-bromo-2-butene

1 h reaction

(*E*)-13-(But-2-en-1-yl)-8*a*-hydroxy-8-methyl-8,13*a*-dihydroazepino[1,2-*a*:3,4-*b'*]diindol-14(8*H*)-one (9)

Using Method B, indigo (262 mg, 1.00 mmol) and (*E*)-1-bromobut-2-ene (670 mg, 5 mmol) were reacted at 85 °C. Upon workup, the filtrate was concentrated affording a red powder, which was recrystallized from CH_2Cl_2 to give **9** as red needle crystals (51.8 mg, 14%).

Two other products were isolated from the treatment of the filtrate from washing of the silica (see method **B**), as compound **4** (31.6 mg, 10%) and compound **14** (135.7 mg, 32%).

Reaction of indigo with 1-bromo-2-butene

3 h reaction

2'-Methyl-1-[1-(but-2-enyl)]-10'-[1-(but-2-enyl)]oxy-2'*H*-spiro(indoline-2,1'-pyrido[1,2-*a*]indol)-3-one (14)

Using Method C, indigo (262 mg, 1.00 mmol) and (*E*)-1-bromobut-2-ene (670 mg, 5 mmol) were reacted at 85–88 °C. The resulting brown orange residue was recrystallised from CH_2Cl_2/Pet. spirit to give 2'-methyl-1-[1-(but-2-enyl)]-10'-[1-(but-

2-enyl)]oxy-2'*H*-spiro(indoline-2,1'-pyrido[1,2-*a*]indol)-3-one (**14**, 169 mg, 40%) as yellow crystals, mp. 147-148 ºC. R_f = 0.51 (CH_2Cl_2/Pet. spirit; 7:3). The X-ray quality crystals were grown through slow crystallization from Pet. spirit : EtOAc (8:2) [4]. The mother liquor was concentrated and subjected to a 30 × 1.5 cm column of silica gel chromatography and resulted in separation of an additional 106 mg of **14** (25%); total yield = 65%. The spectral analysis was compatible with the reported literature values [4].

Reaction of indigo with 1-bromo-3-methyl-2-butene

5 s reaction

***(E)*-1-(3-Methylbut-2-en-1-yl)-[2,2'-biindolinylidene]-3,3'-dione (5)**

8,8-dimethyl-13-(3-methylbut-2-en-1-yl)-7,8-dihydro-6*H*-6,13a-epoxyazepino[1,2-a:3,4-*b*']diindol-14(8*H*)-one (22)

Using Method A, indigo (262 mg, 1.00 mmol) and (*E*)-2-bromo-3-methylbut-2-ene (810 mg, 5 mmol) were reacted at 85 °C. The reaction mix was poured into an ice bath and the dark navy or black precipitate were collected and dissolved in hot CH_2Cl_2 and cooled at 5 °C overnight. The mixture was then filtered to remove the unreacted indigo (41%). The filtrate was concentrated under reduced pressure then dissolved in hot MeOH/H_2O (1:3) and cooled to precipitate **5** (122 mg, 37%) as a dark navy powder, mp. 181-183 ºC, R_f = 0.72 (CH_2Cl_2/Pet. spirit; 7:3). UV–vis (CH_2Cl_2) λ_{max}/nm (ε, $M^{-1}cm^{-1}$) 292 (27415), 639 (16308), 494 (14637). IR $υ_{max}$ 3267 (s), 1627 (s), 1612 (s), 1481 (s), 1462 (s), 1392 (m), 1171 (m), 1130 (m), 1072 (s), 752 (m) cm^{-1}. ^1H NMR δ = 1.68 (s, 3H, H4''), 1.78 (s, 3H, H1'''), 5.12 (bs, 2H, H1''), 5.16 (d, *J* = 4.0 Hz, 1H, H2''), 6.94 (t, *J* = 7.3 Hz, 1H, H5'), 6.99-7.02 (m, 2H, H7, H7') 7.04 (d, *J* = 7.9 Hz, 1H, H5), 7.45 (t, *J* = 6.5 Hz, 1H, H6'), 7.52 (t, *J* = 6.5 Hz, 1H, H6), 7.68 (d, *J* = 7.3 Hz, 1H, H4), 7.72 (d, *J* = 7.3 Hz, 1H, H4') 10.82 (s, 1H, NH). ^{13}C NMR δ = 18.4 (C1'''), 18.9 (C4''), 45.6 (C1''), 112.6 (C7), 113.6

(C5'), 116.4 (C3'a), 119.7 (C7'), 120.3 (C3a), 120.9 (C5), 121.5 (C2), 121.6 (C4), 123.3 (C4'), 123.8 (C2'), 124.3 (C2''), 124.6 (C3''), 136.4 (C6), 136.5 (C6'), 152.4 (C7a), 153.6 (C7'a), 186.6 (C3'), 189.0 (C3). MS (EI): *m/z* = 330 (20%, M^+), 315 (35), 262 (100). HRMS (ESI): calcd for $C_{21}H_{19}N_2O_2$ $[M+H]^+$ 331.1447; found 331.1453. The mother liquor was then dried (Na_2SO_4), concentrated under reduced pressure and subjected to silica gel column chromatography.

Elution with CH_2Cl_2/EtOAc; 9.5:0.5 afforded **22** (83.6 mg, 21%) as a red powder; mp. 74-76 ºC, R_f = 0.66 (CH_2Cl_2/EtOAc (9.5:0.5). UV–vis (CH_2Cl_2) λ_{max}/nm (ε, $M^{-1}cm^{-1}$) 261 (14848), 311 (7945), 364 (9730), 497 (9114), 528 (12003). IR (neat) υ_{max} 3282 (b), 1662 (s), 1616 (s), 1562 (s), 1404 (m), 1107 (s), 744 (s) cm^{-1}. ^1H NMR δ = 1.08 (s, 3H, H1'''b), 1.15 (s, 3H, H1'''a), 1.71 (s, 3H, H1''), 1.85 (s, 3H, H4'), 2.07 (d, *J* = 12.7 Hz, 1H, H7a), 2.36 (dd, *J* = 9.2, 5.8 Hz, 1H, H7b), 4.94 (dd, *J* = 10.5, 5.4 Hz, 1H, H1'a), 5.28 (bt, *J* = 5.4 Hz 1H, H2'), 5.80 (dd, *J* = 11.2, 6.7 Hz, 1H, H1'a), 6.02 (d, *J* = 5.7 Hz, 1H, H6), 6.79-6.82 (m, 2H, H12, H2), 6.87 (d, *J* = 8.3 Hz, 1H, H4), 6.98 (t, *J* = 7.5 Hz, 1H, H10), 7.29-7.39 (m, 3H, H10, H11, H3), 7.73 (d, *J* = 7.7 Hz, 1H, H1). ^{13}C NMR δ = 18.3 (C4'-$\underline{C}H_3$), 23.6 (C1'''b), 25.7 (C1''), 29.2 (C1'''a), 46.4 (C1'), 49.7 (C8), 49.9 (C7), 83.0 (C6), 92.7 (C8a), 108.2 (C4), 109.9 (C12), 111.2 (C13b), 117.4 (C2), 119.9 (C2'), 121.1 (C10), 122.1 (C14a), 124.4 (C1), 125.4 (C8b), 126.3 (C11), 130.4 (C9), 133.3 (C3), 135.3 (C3'), 145.8 (C4a), 147.3 (C12a), 149.3 (C13a), 177.5 (C14). MS (EI): *m/z* = 398 (100% M^+), 342 (63), 329 (50), 274 (78). HRMS (ESI): calcd for $C_{26}H_{27}N_2O_2$ $[M+H]^+$ 399.2073; found 399.2085.

Reaction of indigo with 1-bromo-3-methyl-2-butene

1 h reaction

8a-Hydroxy-8,8-dimethyl-8,13a-dihydroazepino[1,2-*a*:3,4-*b*']diindol-14(8*H*)one (10)

8',8'-dimethyl-1-(3-methylbut-2-en-1-yl)-10'-((3-methylbut-2-en-1-yl)oxy)-8'*H*-spiro[indoline-2,9'-pyrido[1,2-*a*]indol]-3-one (15)

Using Method B, indigo (262 mg, 1.00 mmol) and (*E*)-2-bromo-3-methylbut-2-ene (810 mg, 5 mmol) were reacted at 85 °C. Upon workup, the filtrate was concentrated affording a red powder which was recrystallized from Pet. spirit/EtOAc (9:1) to deposit orange red crystals of **10** (105 mg, 32%); mp 190-192 ºC, R_f = 0.44 (CH_2Cl_2/EtOAc; 9.5:0.5). The X-ray quality crystals were grown through slow crystallization from Pet. spirit/EtOAc (9:1). UV–vis (CH_2Cl_2) λ_{max}/nm (ε, $M^{-1}cm^{-1}$) 288 (16535), 301 (16077), 371 (13040), 490 (15245). IR υ_{max} 3282 (b), 1662 (s), 1616 (s), 1562 (s), 1404 (m), 1107 (s), 744 (s) cm^{-1}. ^1H NMR δ = 0.80 (s, 3H, C1'a), 1.63 (s, 3H, C1'b), 4.82 (d, *J* = 10.1 Hz, 1H, H7), 6.83 (d, *J* = 10.1 Hz, 1H, H8), 6.96 (d, *J* = 7.9 Hz, 1H, H12), 7.01 (t, *J* = 7.2 Hz, 2H, H2, H10), 7.20 (d, *J* = 8.3 Hz, 1H, H4), 7.30 (t, *J* = 7.9 Hz, 1H, H11), 7.40 (t, *J* = 8.3, 1H, H3), 7.60 (d, *J* = 7.5 Hz, 1H, H9), 7.77 (d, *J* = 7.5 Hz, 1H, H1). ^{13}C NMR δ = 26.7 (C1'b), 27.3 (C1'a), 44.3 (C8), 85.8 (C8a), 109.5 (C4), 110.9 (C12), 111.5 (C7), 114.1 (C13b), 120.6 (C6), 120.8 (C2), 121.7 (C10), 122.8 (C14a), 123.7 (C1), 126.6 (C9), 129.6 (C8b), 130.4 (C11), 133.8 (C3), 144.2 (C12a), 146.0 (C4a), 149.5 (C13a), 182.1 (C14). MS (EI): *m/z* = 330 (75%, M^+), 315 (100), 262 (93), HRMS (ESI): calcd for $C_{21}H_{19}N_2O_2$ $[M+H]^+$ 331.1447; found 331.1438.

The mother liquor and filtrate from the elution of the silica with CH_2Cl_2 were combined and subjected to 40 × 1.5 cm silica gel column chromatography. Elution with CH_2Cl_2/Pet. spirit; 9:1, yielded **15** (56 mg, 21%) as a yellow amorphous solid; R_f = 0.96 (CH_2Cl_2 : Pet. spirit; 9:1). IR υ_{max} 1701 (m), 1612 (m), 1465 (m), 1261 (m), 1095 (s), 802 (s), 742 (m) cm^{-1}. ^1H NMR δ = 1.14 (s, 3H, C3"a-CH_3), 1.22 (s,

3H, C4''-CH_3), 1.42 (s, 3H, C3'''a-CH_3), 1.50 (s, 3H, H8'a-(1×CH_3)), 1.52 (s, 3H, H8'b-(1×CH_3)), 1.63 (s, 3H, C4'''-CH_3), 3.87-3.90 (m, 1H, H1''a), 4.06-4.15 (m, 2H, H1''b, H1'''a), 4.30-4.33 (m, 1H, H1'''b), 5.02 (bs, 1H, H2''), 5.08 (t, J = 7.1 Hz, 1H, H2'''), 5.21 (d, J = 7.7 Hz, 1H, H7'), 6.72 (m, 2H, H5, H7), 7.00 (d, J = 7.7 Hz, 1H, H6'), 7.06 (t, J = 7.4 Hz, 1H, H2'), 7.20 (t, J = 8.2 Hz, 1H, H3'), 7.33 (d, J = 8.3 Hz, 1H, H4'), 7.40 (t, J = 7.4 Hz, 1H, H6), 7.51 (d, J = 7.8 Hz, 1H, H1'), 7.58 (d, J = 7.7 Hz, 1H, H4). ^{13}C NMR δ = 18.1 (C8'-$\underline{C}H_3$a), 18.2 (C8'-$\underline{C}H_3$b), 23.8 (C4'''), 24.2 (C3'''a), 25.5 (C4''), 26.0 (C3''a), 39.4 (C8'), 44.2 (C1''), 71.0 (C1'''), 72.4 (C2), 108.6 (C4'), 109.2 (C5), 117.2 (C7), 117.9 (C2'''), 118.9 (C1'), 119.3 (C7'), 119.4 (C10'), 120.3 (C2'), 120.8 (C6'), 120.9 (C3a), 121.7 (C2''), 122.4 (C10'a), 123.2 (C3'), 124.7 (C4), 132.1 (C9'a), 133.2 (C4'a), 137.3 (C3'''), 137.4 (C6), 137.9 (C3''), 161.3 (C7a), 199.1 (C3). MS (EI): *m/z* = 466 (12%, M^+), 397 (79), 329 (100), HRMS (ESI): calcd for $C_{31}H_{35}N_2O_2$ $[M+H]^+$ 467.2699; found 467.2708.

Further elution with CH_2Cl_2/EtOAc (95:5) gave **22** (103 mg, 26%).

Reaction of indigo with 1-bromo-3-methyl-2-butene

3 h reaction

8',8'-Dimethyl-1-(3-methylbut-2-en-1-yl)-10'-((3-methylbut-2-en-1-yl)oxy)-8'*H*-spiro[indoline-2,9'-pyrido[1,2-*a*]indol]-3-one (15)

Using Method C, indigo (262 mg, 1.00 mmol) and (*E*)-2-bromo-3-methylbut-2-ene (810 mg, 5 mmol) were reacted at 85 °C for 3 h. The filtrate was subjected to flash silica gel column chromatography and elution with CH_2Cl_2/Pet. spirit (9:1) yielded **15** (195 mg, 42%) as a yellow amorphous solid. Further elution with CH_2Cl_2/EtOAc (95:5) gave **22** (93.5 mg, 23%).

Reaction of indigo with 3-bromo-1-phenyl-1-propene (cinnamyl bromide)

5 s reaction

(2*E*)-1-(3-Phenylallyl)-[2,2'-biindolinylidene]-3,3'-dione (6)

Using Method A, indigo (262 mg, 1.00 mmol) and cinnamyl bromide (980 mg, 5 mmol) were reacted at 85 °C for 5 s. The reaction mixture was poured into an ice bath and the black precipitate was collected, dissolved in hot CH_2Cl_2 and was cooled at 5 °C overnight. The mixture was then filtered to remove the unreacted indigo (38%). The filtrate was concentrated under reduced pressure and recrystallised from Pet. spirit/CH_2Cl_2 (90:10) to furnish **6** (181 mg, 48%) as a navy powder, mp. 192-194 ºC, R_f = 0.72 (CH_2Cl_2/Pet. spirit; 7:3). UV–vis (CH_2Cl_2) λ_{max}/nm (ε, $M^{-1}cm^{-1}$) 291 (28197), 628 (15596). IR υ_{max} 3259 (m), 1685 (w), 1639 (s), 1608 (s), 1465 (s), 1388 (s), 1296 (m), 1068 (s), 1018 (s), 918 (m), 748 (s) cm^{-1}. ^{1}H NMR δ = 5.28 (t, *J* = 5.3 Hz, 2H, H1''), 6.24-6.30 (m, 1H, H2''), 6.48 (d, *J* = 16.1 Hz, 1H, H3''), 6.91 (t, *J* = 6.9 Hz, 1H, H5), 6.96 (d, *J* = 7.0 Hz, 1H, H7), 7.00 (d, *J* = 8.3 Hz, 1H, H6), 7.15 (t, *J* = 7.2 Hz, 2H, H7', HAr), 7.21-7.29 (m, 4H, HAr), 7.42 (t, *J* = 7.4 Hz, 1H, H5'), 7.50 (t, *J* = 7.5 Hz, 1H, H6'), 7.65 (d, *J* = 7.7 Hz, 1H, H4), 7.73 (d, *J* = 7.7 Hz, 1H, H4') 10.70 (s, 1H, NH). ^{13}C NMR δ = 49.4 (C1''), 111.2 (C7), 111.9 (C5'), 120.1 (C2), 120.7 (C3''), 120.9 (C7'), 121.1 (C2'), 122.8 (C3a), (CAr), 124.1 (C4'), 124.6 (C5), 124.8 (C4), 125.8 (C3'a), 126.45 (2CAr), 127.7 (CAr), 128.5 (2CAr), 132.4 (C2''), 135.9 (C6), 136.1 (C6'), 136.4 (CAr), 151.5 (C7a), 152.8 (C7'a), 193.1 (C3'), 196.4 (C3). MS (EI): *m/z* = 378 (100%, M^+), HRMS (ESI): calcd for $C_{25}H_{19}N_2O_2$ $[M+H]^+$ 379.1447; found 379.1444.

Reaction of indigo with 3-bromo-1-phenyl-1-propene

1 h reaction

(8'*R*)-9a'-Cinnamyl-8'-phenyl-8'*H*-spiro[indoline-2,9'-pyrido[1,2-*a*]indole]-3,10'(9a'*H*)-dione (23)

8'-phenyl-1-(3-phenylallyl)-10'-((3-phenylallyl)oxy)-8'*H*-spiro[indoline-2,9'-pyrido[1,2-*a*]indol]-3-one (16)

Using Method B, indigo (262 mg, 1.00 mmol) and cinnamyl bromide (980 mg, 5.00 mmol) were reacted at 85 °C for 1 h. Upon workup, the residue was subjected to a silica gel column chromatography. Elution with CH_2Cl_2/Pet. spirit (9:1) yielded **16** (165 mg, 37%) as a yellow crystalline solid, with spectral values similar with the reported data [4]. Further elution, yielded another yellow fraction which was concentrated. The residue was dissolved in a minimum volume of CH_2Cl_2 (5–6 mL) and Pet. spirit (5–7 mL) was then added dropwise until the solution turned cloudy. The solution was heated in a water bath and when it became clear, it was cooled to precipitate **23** (79.4 mg, 16%) as a luminous yellow powder, mp. 269-271 ºC, R_f = 0.37 (CH_2Cl_2). X-ray quality crystals (DMSO solvate) were obtained from a DMSO solution by evaporation under stream of N_2. UV–vis (CH_2Cl_2) λ_{max}/nm (ε, $M^{-1}cm^{-1}$) 285 (15395), 392 (3647). IR (neat) υ_{max} 3356 (m), 3232 (m), 1693 (s), 1662 (s), 1612 (s), 1473 (s), 1233 (m), 752 (s) cm^{-1}. ^{1}H NMR ($(CD_3)_2SO$) δ = 2.98-3.10 (m, 2H, H1''), 4.12 (s, 1H, H8'), 4.98 (d, *J* = 7.7 Hz, 1H, H7'), 5.70-5.76 (m, 1H, H2''), 6.31 (t, *J* = 7.2 Hz, 1H, H5), 6.43 (d, *J* = 15.7 Hz, 1H, H3''), 6.73-6.76 (m, 3H, H3', H6, H7), 6.99-7.07 (m, 5H, HAr''), 7.13 (d, *J* = 6.9 Hz, 1H, H4), 7.02-7.23 (m, 5H, HAr'), 7.26 (d, *J* = 7.7 Hz, 1H, H4'), 7.42 (t, *J* = 8.0 Hz, 2H, H6', H1'), 7.52 (t, *J* = 7.4 Hz, 1H, H2'), 7.87 (s, 1H, N<u>H</u>). ^{13}C NMR δ = 37.1 (C1'), 44.8 (C8'), 68.9 (C2), 69.9 (C9'a), 104.8 (C7'), 110.5 (C1'), 111.5 (C5), 117.0 (C3'), 119.6 (C7), 120.6 (C3a), 122.5 (C4'a), 122.7 (C6), 123.3 (C2'), 123.9 (C4'), 124.7 (C6'), 126.3 (3CAr''), 127.3 (CAr''), 127.7 (CAr''), 128.0 (CAr'), 129.0 (3CAr'), 130.1 (CAr'), 134.3 (C3''), 136.9 (C4), 137.2 (CAr'), 137.7 (CAr''), 138.1 (C2'), 157.2 (C10'a), 161.4 (C7a), 197.5 (C3), 197.8 (C10). MS (EI): *m/z* = 494 (22%,

M^+), 377 (100). HRMS (ESI): calcd for $C_{34}H_{27}N_2O_2$ $[M+H]^+$ 495.2073; found 495.2054.

Reaction of indigo with 3-bromo-1-phenyl-1-propene

3 h reaction

8'-Phenyl-1-(3-phenylallyl)-10'-((3-phenylallyl)oxy)-8'*H*-spiro[indoline-2,9'-pyrido[1,2-*a*]indol]-3-one (16)

Using Method C, indigo (262 mg, 1.00 mmol) and 3-bromo-1-phenyl-1-propene (cinnamyl bromide) (980 mg, 5 mmol) were reacted at 85 °C for 3 h. The concentrated filtrate was subjected to a flash silica gel column chromatography and elution with CH_2Cl_2/Pet. spirit (9:1) yielded **16** (226 mg, 37%) as a yellow crystalline solid, with spectral values similar to those reported [4].

Optimised procedure of production of monoallylated indigos 2–4.

A suspension of indigo (262 mg 1.00 mmol) in anhydrous DMF (40 mL) was sonicated for 30 min and the resulting suspension was transferred to a septum equipped round bottom flask carrying pre-dried Cs_2CO_3 (1.303 g, 4.00 mmol) under a N_2 flow. The flask was plunged into a preheated oil bath at 85–88 °C and stirred for 1 h. The N_2 flow was stopped and the allylic bromide (5.00 mmol) was added rapidly in one portion by syringe, and after 5 s, the reaction mixture was poured into an ice bath. The blue-black precipitate was filtered and dissolved in hot CH_2Cl_2 and cooled at 5 °C overnight. The mixture was then filtered to remove the unreacted indigo (10% >). The filtrate was concentrated under reduced pressure then recrystallised from Pet. spirit/EtOAc (90:10) to furnish the mono-allylated indigos **2**–**4** in 83%, 81% and 89% yields, respectively.

Ring-closing metathesis of 1-allyl-10'-allyloxy-2'*H*-spiro(indoline-2,1'-pyrido[1,2-*a*]indol)-3-one (12)

A mixture of the spiro derivative **12** (40 mg, 0.95 mmol) and Grubbs' II catalyst (5 mg, 10% mmol) in CH_2Cl_2 (40 mL) was heated at reflux for 1 h. The reaction mixture was then filtered and the filtrate was subjected to flash silica gel column chromatography and elution with Pet. spirit /CH_2Cl_2 (1:9) yielded 1,2-dihydro-1-vinyl-8*H*,9*H*,17*H*-benz[2',3']pyrrolizino-[1',7'*a*:2,3]pyrido[1,2-*a*]indole-8,17-dione (**31**) as a yellow powder (26 mg, 70%); mp 229-231 °C, R_f = 0.81 (CH_2Cl_2 : Pet. spirit; 9:1). X-ray quality crystals were grown through slow crystallization from Pet. spirit : CH_2Cl_2 (5:3). UV–vis (CH_2Cl_2) λ_{max}/nm (ε, $M^{-1}cm^{-1}$) 286 (8458), 401 (3888). IR (neat) $υ_{max}$ 1685 (s), 1603 (s), 1473 (s), 1318 (m), 1147 (m) cm^{-1}. ^1H NMR δ = 2.53-2.65 (m, 2H, H9a,b), 3.58-3.55 (m, 1H, H1), 3.80 (t, *J* = 9.6 Hz, 1H, H2a), 3.88-3.94 (m, 1H, H2b), 4.93 (dd, *J* = 1.2, 10.0 Hz, 1H, H2'a), 5.10 (bs, ½H, H2'b), 5.15-5.16 (m, ½H, H2'a), 5.17-5.21 (m, 1H, H10), 5.45-5.56 (m, 1H, H1'), 6.79-6.85 (m, 2H, H5, H15), 6.86-6.92 (m, 2H, H4, H13), 7.08 (d, *J* = 8.4 Hz, 1H, H11), 7.36-7.50 (m, 3H, H6, H7, H16), 7.54-7.60 (m, 1H, H14). ^{13}C NMR δ = 29.1 (C9), 49.5 (C2), 54.7 (C1), 71.3 (C8a), 71.8 (C17a), 101.5 (C10), 110.8 (C11), 112.1 (C13), 120.2 (C5), 120.3 (C2'), 120.6 (C15), 122.2 (C16a), 123.1 (C4), 123.7 (C7a), 124.5 (C7), 124.7 (C16), 131.7 (C1'), 137.5 (C6), 137.7 (C14), 158.0 (C12a), 164.4 (C3a), 197.9 (C17), 201.0 (C8). MS (EI): *m/z* = 354 (100%, M^+), HRMS (ESI): calcd for $C_{23}H_{19}N_2O_2$ $[M+H]^+$ 355.1447; found 355.1434.

Reaction of 13-allyl-8a-hydroxy-8,13a-dihydroazepino[1,2-*a*:3,4-*b*']diindol-14(8*H*)-one (7) with Cs_2CO_3

A solution of **7** (34.2 mg, 0.100 mmol) in DMF (8 mL) was transferred to a septum-equipped round bottom flask containing pre-dried Cs_2CO_3 (0.65 mg, 0.200 mmol) under an inert atmosphere. The mixture was heated and stirred for 20 min and

then was poured into an ice bath. The cloudy solution was partitioned in CH_2Cl_2 and the combined organic layers were washed with brine (2 × 30 mL) and water (5 × 50 mL), dried (Na_2SO_4) and concentrated under reduced pressure. The residue was subjected to PTLC and elution with Pet. spirit/CH_2Cl_2 (1:3) yielded 8*H*,16*H*-pyrido[1,2,3-*s,t*]-indolo[1,2-*a*]azepino[3,4-*b*]indol-17-one (**17**, 28.6 mg, 89%) as a yellow-brown powder; mp 143-144 °C [4].

Allylation of (*E*)-1-allyl-[2,2'-biindolinylidene]-3,3'-dione (2)

A solution of **2** (30.2 mg, 0.100 mmol) in DMF (8 mL) was transferred to a septum-equipped round flask containing 4 Å molecular sieves, pre-dried Cs_2CO_3 (0.65 mg, 0.200 mmol) and allyl bromide (53.6 mg, 0.400 mmol) in DMF (2 mL) under an inert atmosphere. The mixture was heated and stirred for 3 h. The reaction mixture was then filtered into an ice bath. The cloudy solution was partitioned in CH_2Cl_2 (ca. 10 mL) and the combined organic layers were washed with brine (2 × 30 mL) and water (5 × 50 mL), dried (Na_2SO_4) and concentrated under reduced pressure. The filtrate was subjected to PTLC and elution with Pet. spirit/CH_2Cl_2 (1:3) yielded 8*H*,16*H*-pyrido[1,2,3-*s,t*]-indolo[1,2-*a*]azepino[3,4-*b*]indol-17-one (**17**, 19.1 mg, 59%) as a yellow-brown powder; mp 143-144 °C. X-ray quality crystals were grown through the slow crystallisation from Pet. spirit : CH_2Cl_2 (5:3). R_f = 0.61. Results from spectral analysis of the purified compound were compatible with the reported literature values [4]. The other distinctive band was collected and soaked in EtOAc to yield 1-allyl-10'-allyloxy-2'*H*-spiro(indoline-2,1'-pyrido[1,2-*a*]indol)-3-one (**12**, 2.30 mg, 8%) as an orange yellow solid; mp. 121-122 ºC. R_f = 0.51. The spectral information was compatible with literature reported values [4].

NMR Spectra of the new compounds

N-Monoallylated compounds from the 5 s reaction

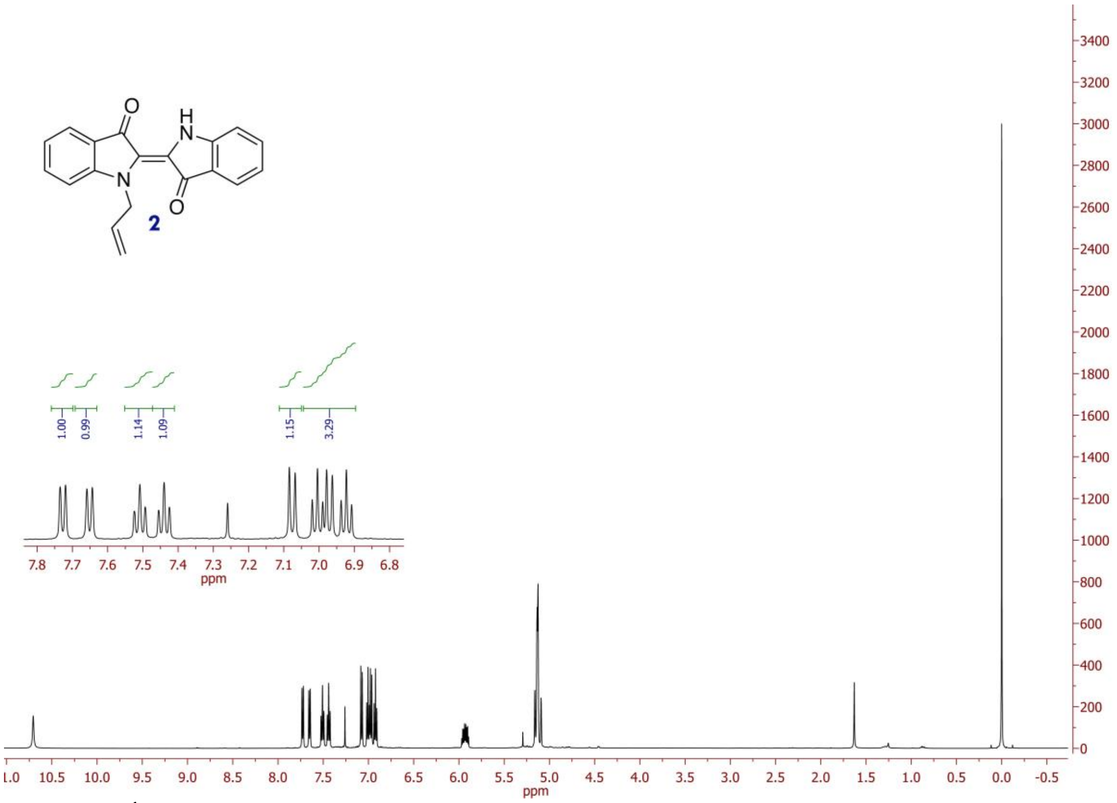

Figure S1: ^{1}H NMR spectrum, recorded in $CDCl_3$ for (*E*)-1-allyl-[2,2'-biindolinylidene]-3,3'-dione **(2)**

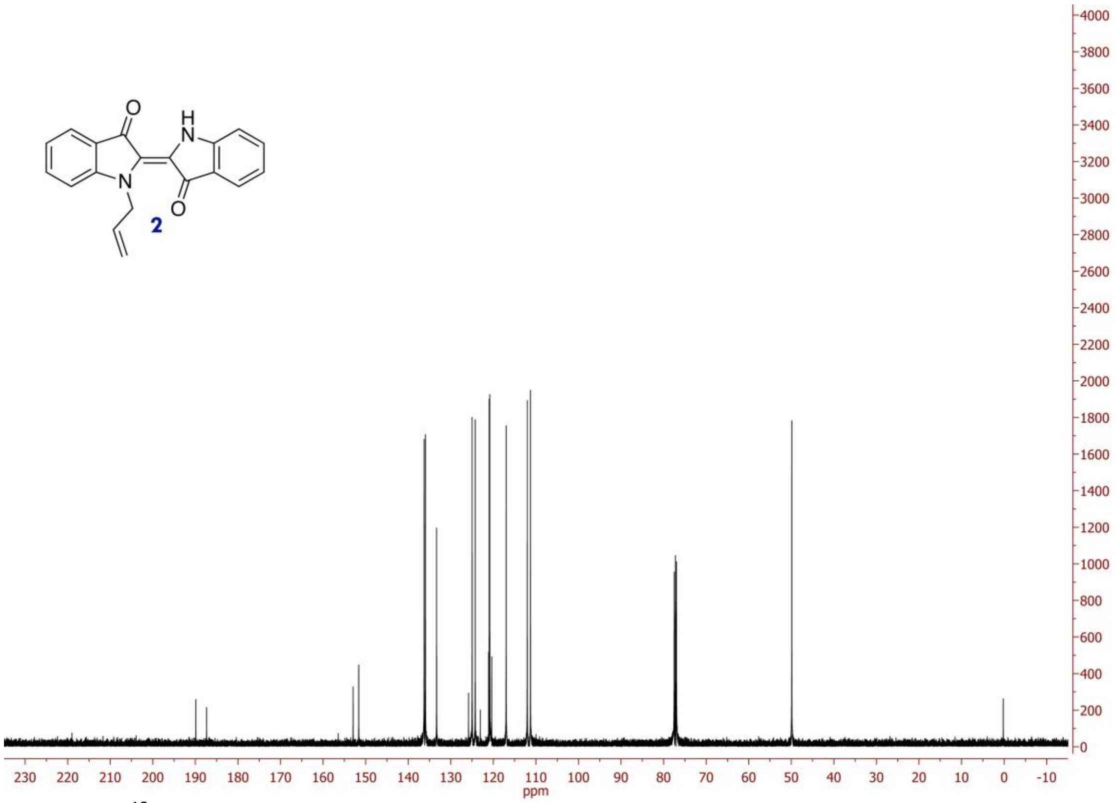

Figure S2: ^{13}C NMR spectrum, recorded in $CDCl_3$ for (*E*)-1-allyl-[2,2'-biindolinylidene]-3,3'-dione **(2)**

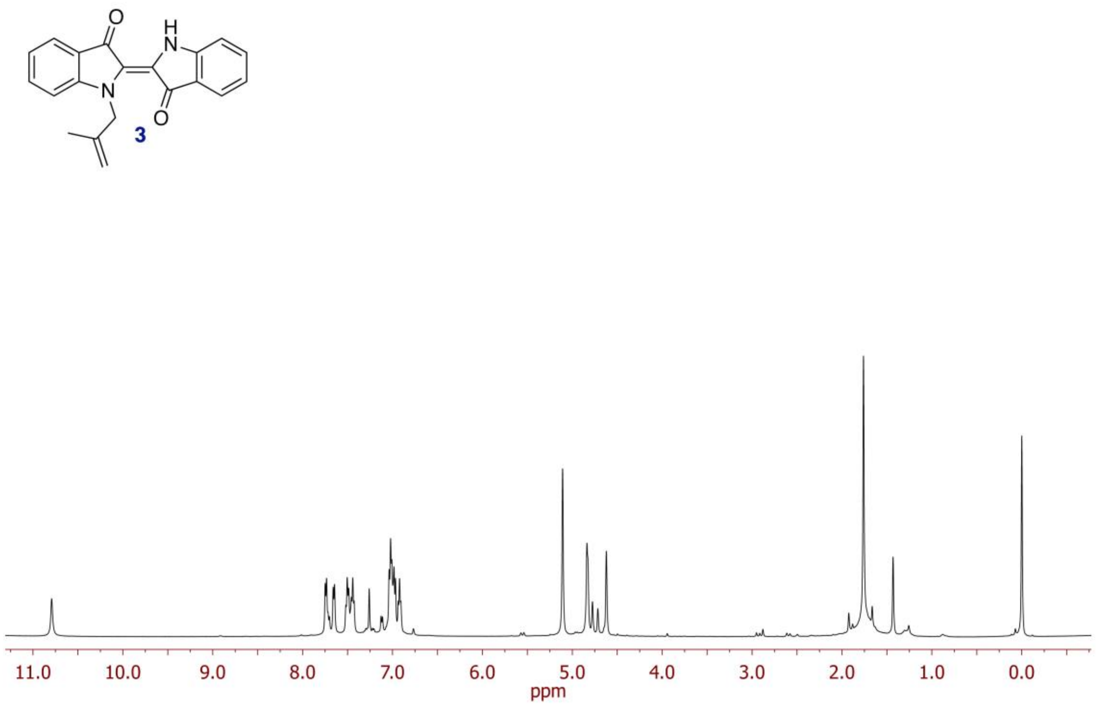

Figure S3: ^{1}H NMR spectrum, recorded in (CD3)2SO for (E)-1-(2-methylallyl)-[2,2'-biindolinylidene]-3,3'-dione (**3**)

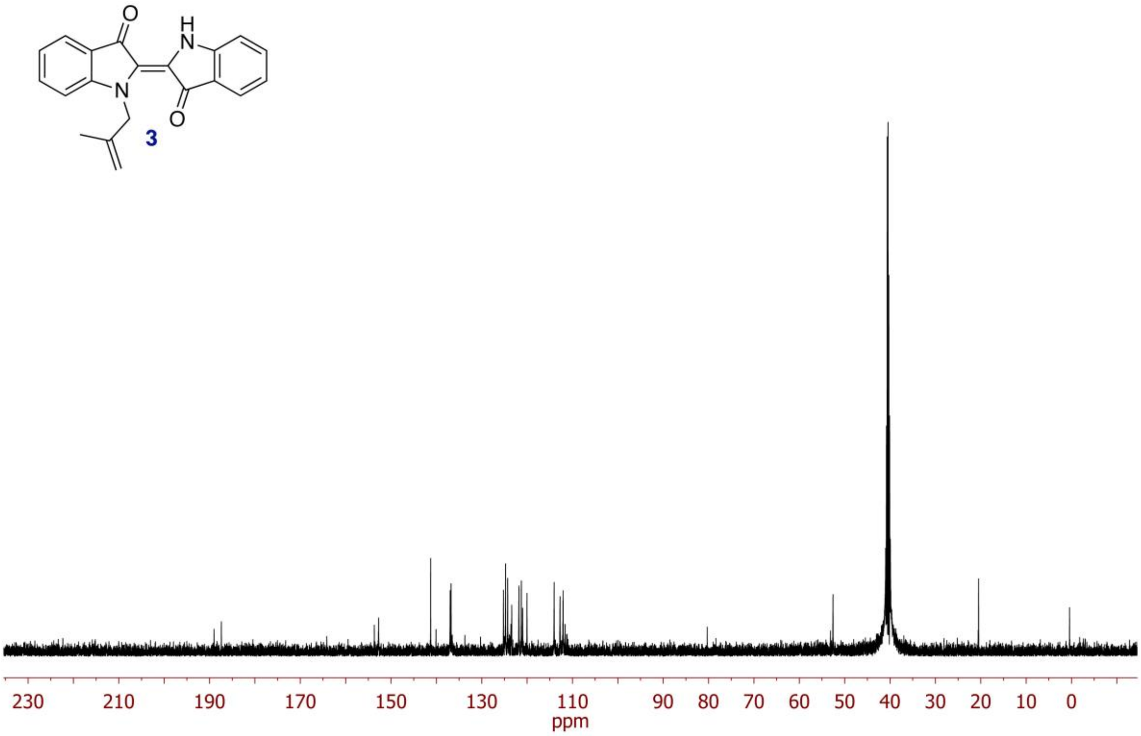

Figure S4: 13C NMR spectrum, recorded in (CD3)2SO for (E)-1-(2-methylallyl)-[2,2'-biindolinylidene]-3,3'-dione (**3**)

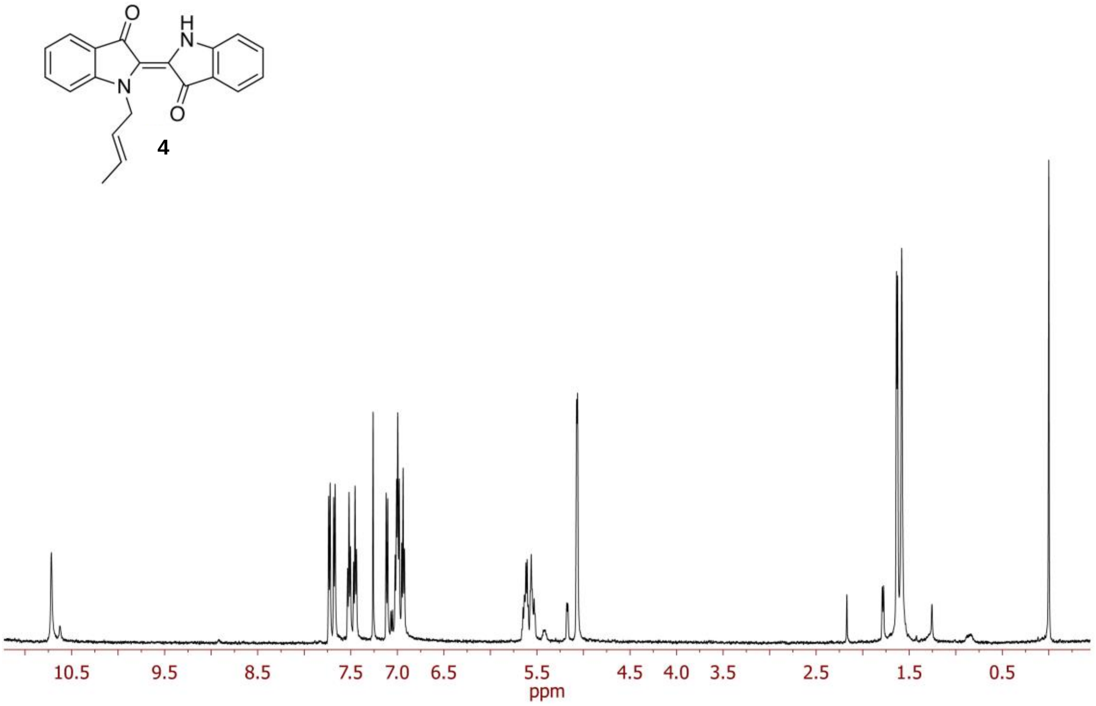

Figure S5: ^{1}H NMR spectrum, recorded in $CDCl_3$ for (*E*)-1-((*E*)-but-2-en-1-yl)-[2,2'-biindolinylidene]-3,3'-dione **(4)**

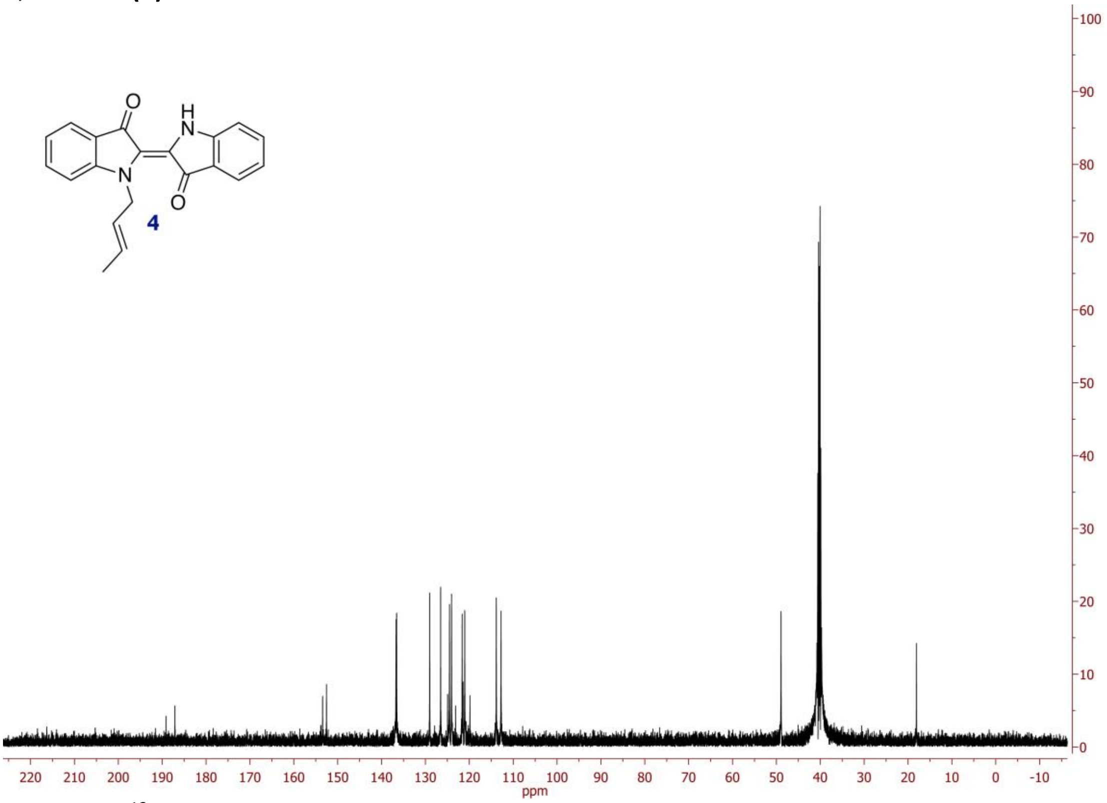

Figure S6: ^{13}C NMR spectrum, recorded in $(CD_3)_2SO$ for (*E*)-1-((*E*)-but-2-en-1-yl)-[2,2'-biindolinylidene]-3,3'-dione **(4)**

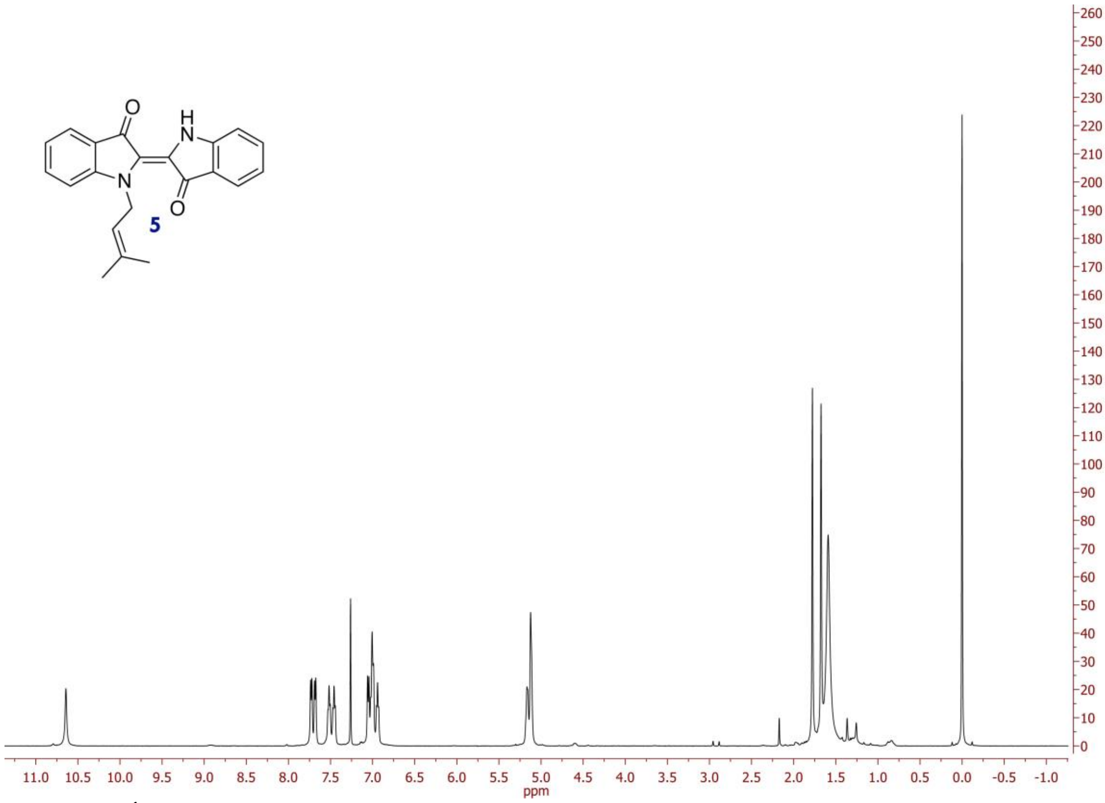

Figure S7: ^{1}H NMR spectrum, recorded in $CDCl_3$ for (*E*)-1-(3-methylbut-2-en-1-yl)-[2,2'-biindolinylidene]-3,3'-dione **(5)**

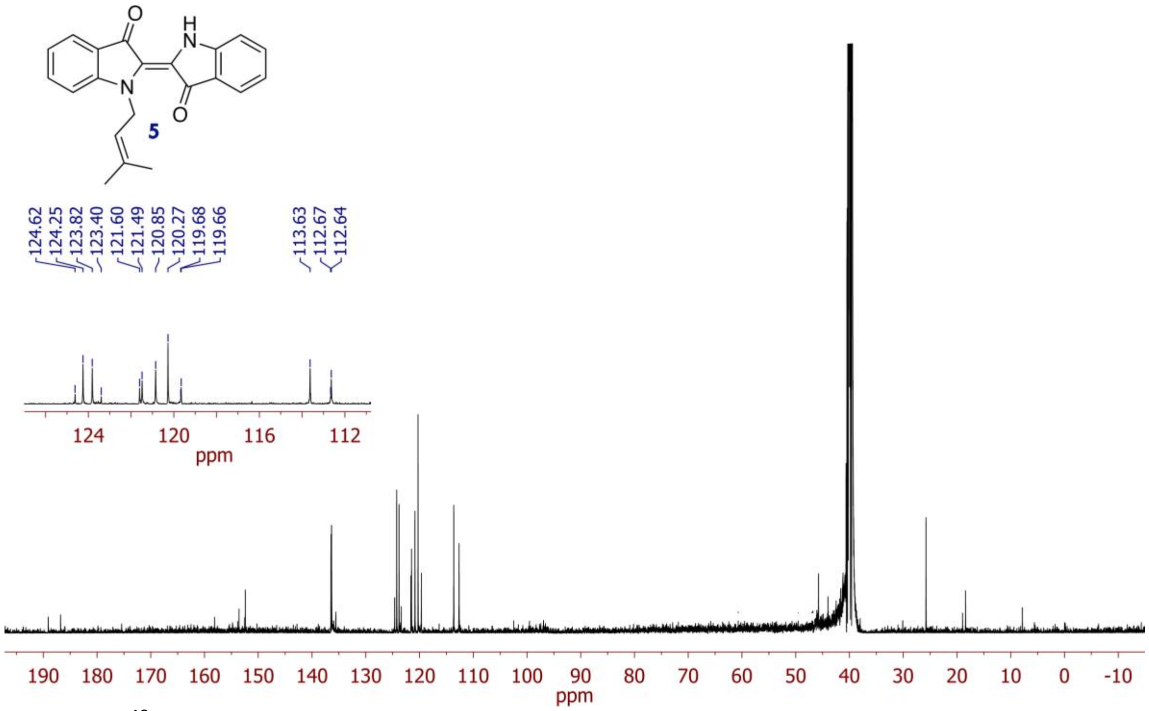

Figure S8: ^{13}C NMR spectrum, recorded in $(CD_3)_2SO$ for (*E*)-1-(3-methylbut-2-en-1-yl)-[2,2'-biindolinylidene]-3,3'-dione **(5)**

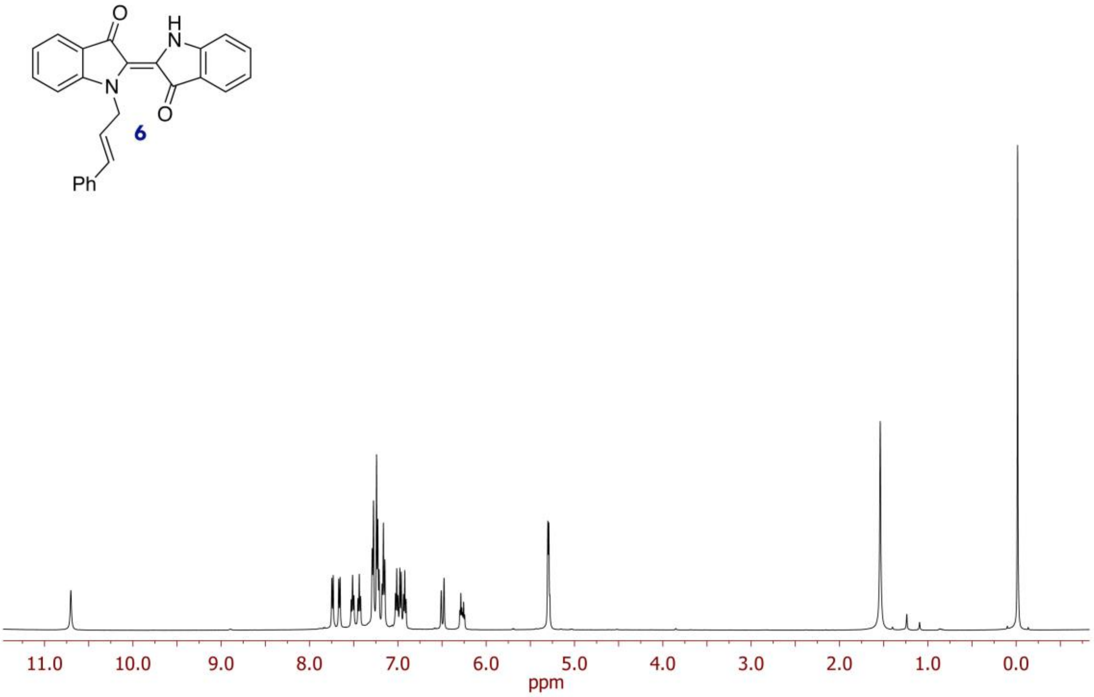

Figure S9: ^{1}H NMR spectrum, recorded in $CDCl_3$ for (2*E*)-1-(3-phenylallyl)-[2,2'-biindolinylidene]-3,3'-dione **(6)**

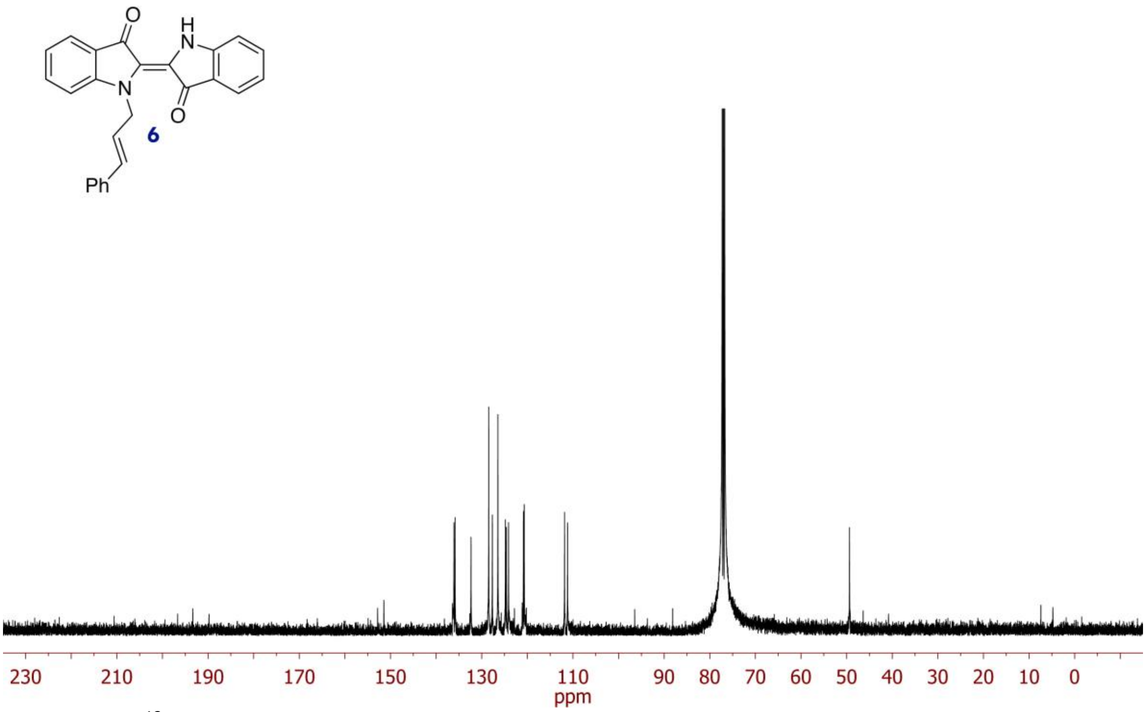

Figure S10: ^{13}C NMR spectrum, recorded in $CDCl_3$ for (2*E*)-1-(3-phenylallyl)-[2,2'-biindolinylidene]-3,3'-dione **(6)**

Azepinodiindolones from the 1 h reaction

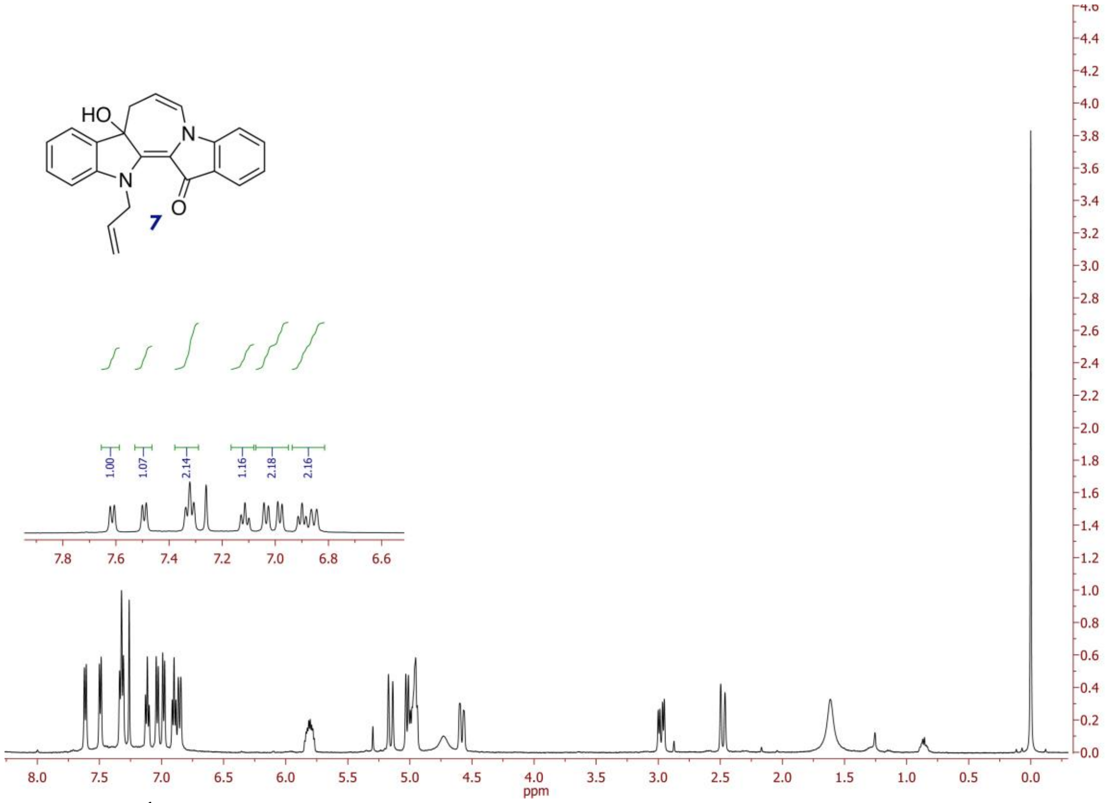

Figure S11: ^{1}H NMR spectrum, recorded in $CDCl_3$ for 13-allyl-8a-hydroxy-8,13a-dihydroazepino[1,2-*a*:3,4-*b*′]diindol-14(8*H*)-one **(7)**

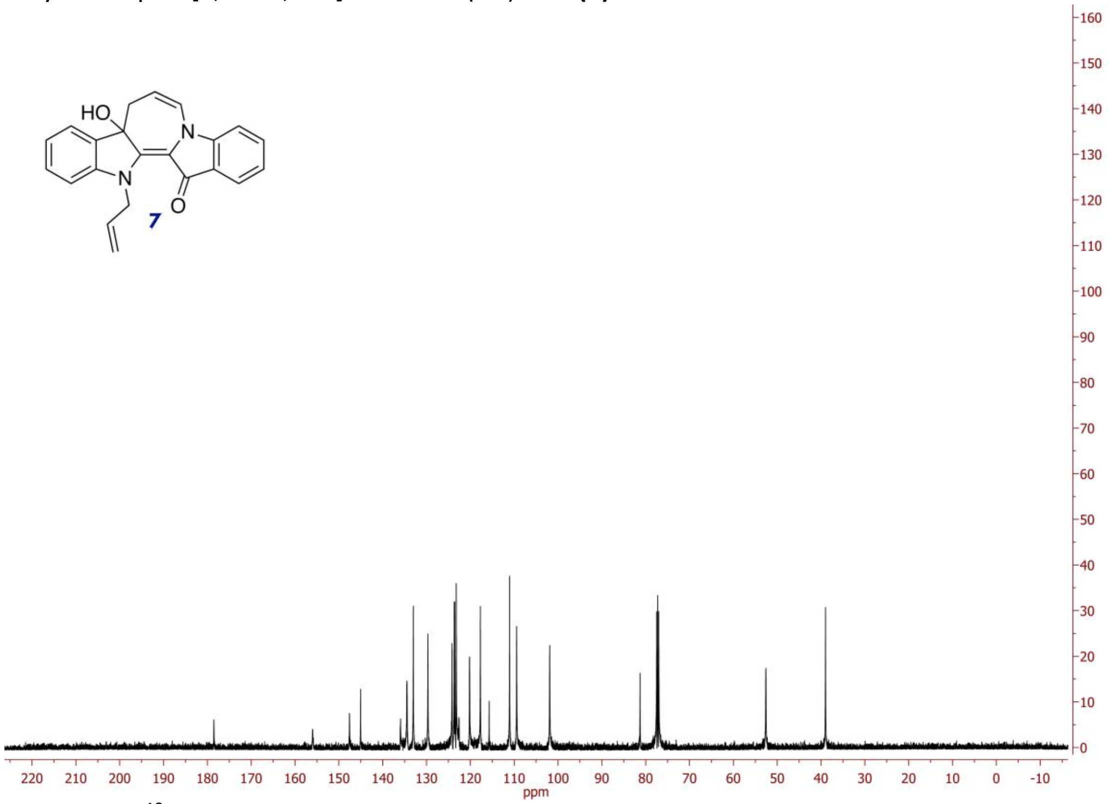

Figure S12: ^{13}C NMR spectrum, recorded in $CDCl_3$ for 13-allyl-8a-hydroxy-8,13a-dihydroazepino[1,2-*a*:3,4-*b*′]diindol-14(8*H*)-one **(7)**

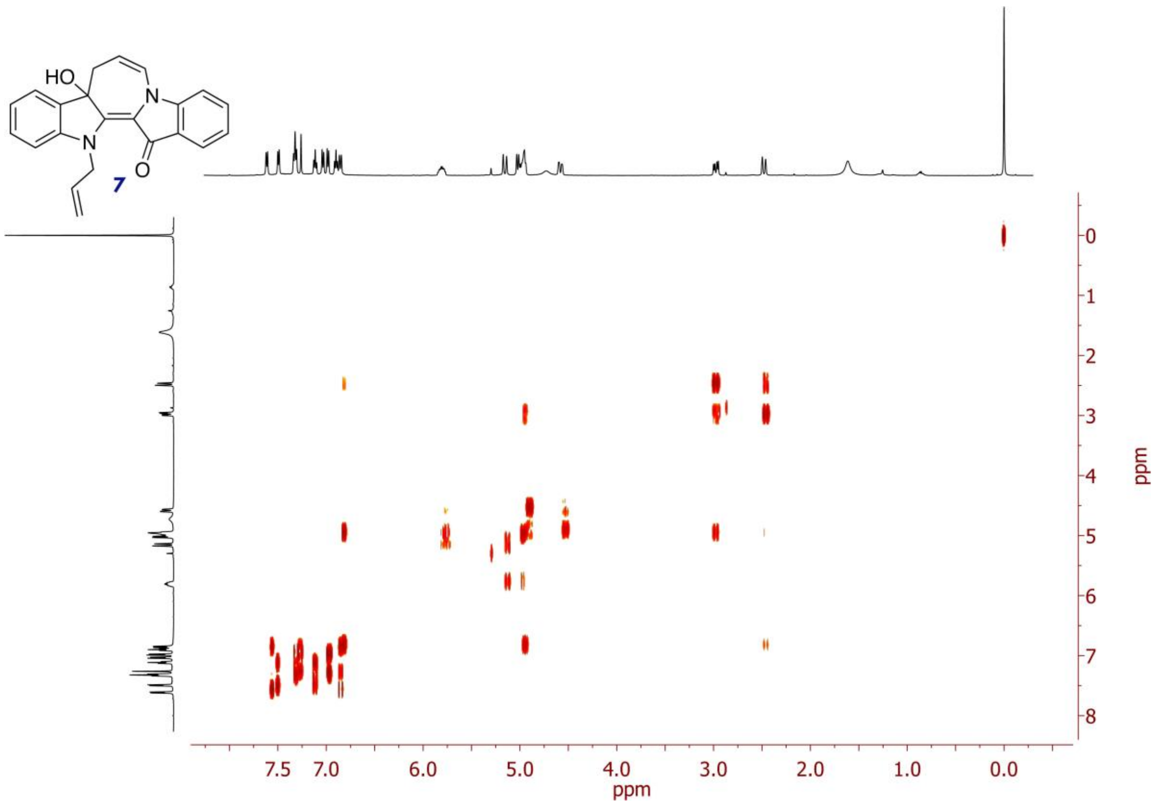

Figure S13: COSY spectrum, recorded in $CDCl_3$ for 13-allyl-8a-hydroxy-8,13a-dihydroazepino[1,2-*a*:3,4-*b'*]diindol-14(8*H*)-one **(7)**

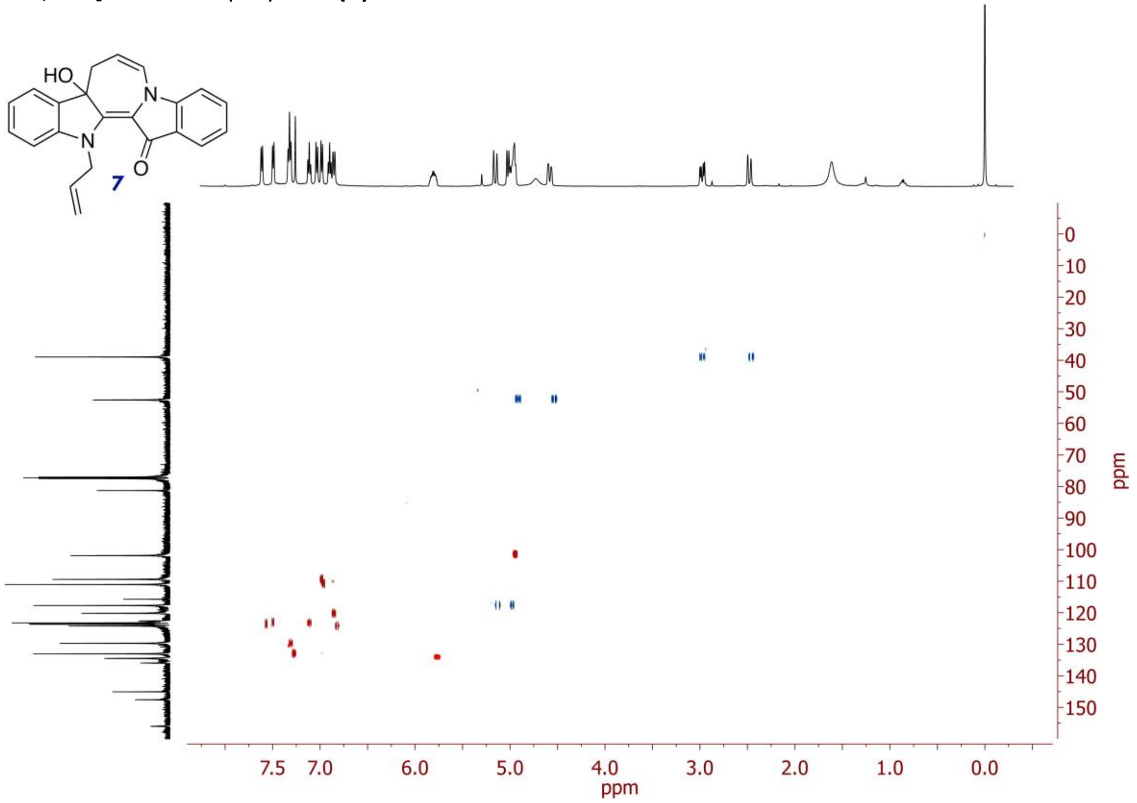

Figure S14: HSQC spectrum, recorded in $CDCl_3$ for 13-allyl-8a-hydroxy-8,13a-dihydroazepino[1,2-*a*:3,4-*b'*]diindol-14(8*H*)-one **(7)**

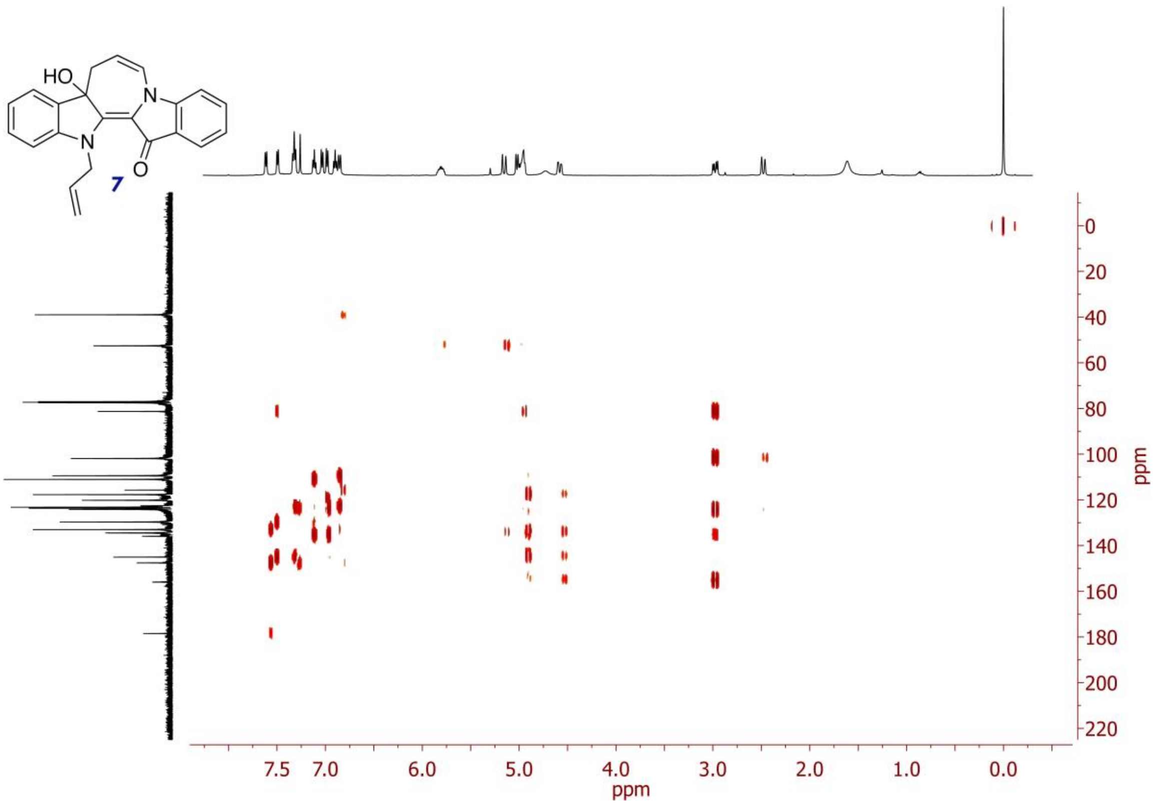

Figure S15: HMBC spectrum, recorded in $CDCl_3$ for 13-allyl-8a-hydroxy-8,13a-dihydroazepino[1,2-*a*:3,4-*b*']diindol-14(8*H*)-one **(7)**

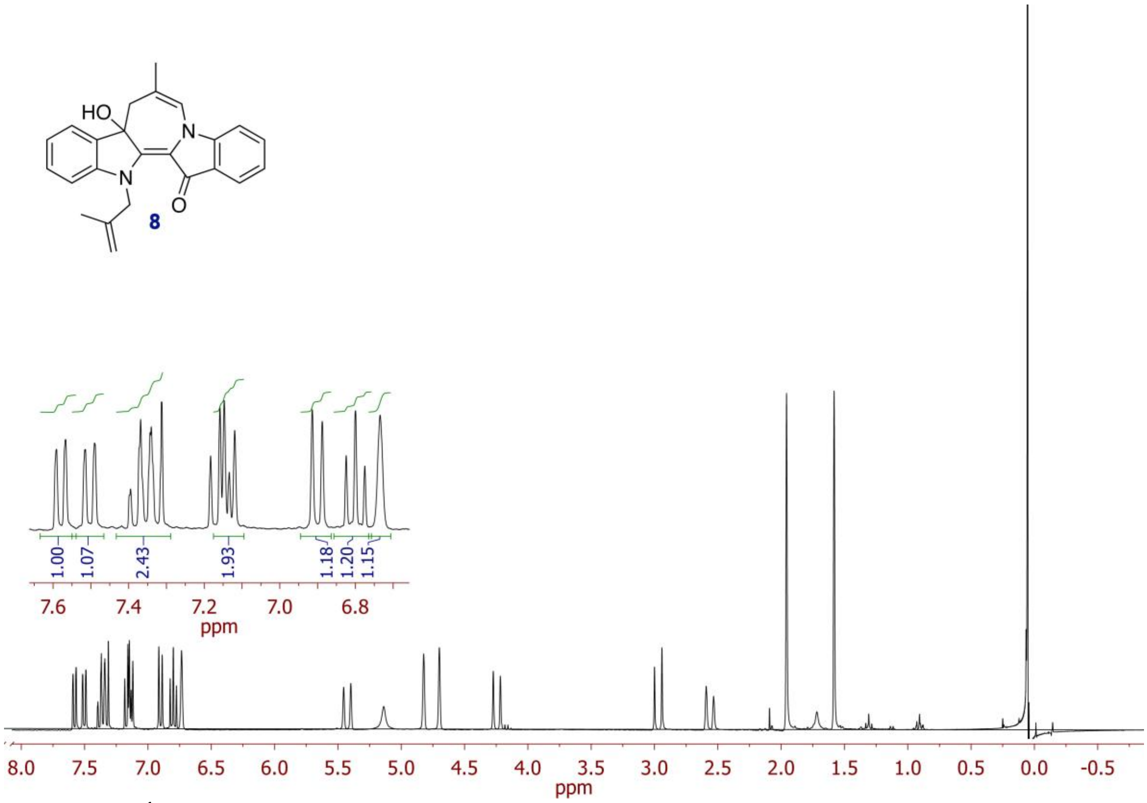

Figure S16: ^{1}H NMR spectrum, recorded in $CDCl_3$ for 8a-hydroxy-7-methyl-13-(2-methylallyl)-8,13a-dihydroazepino[1,2-*a*:3,4-*b'*]diindol-14(8*H*)-one **(8)**

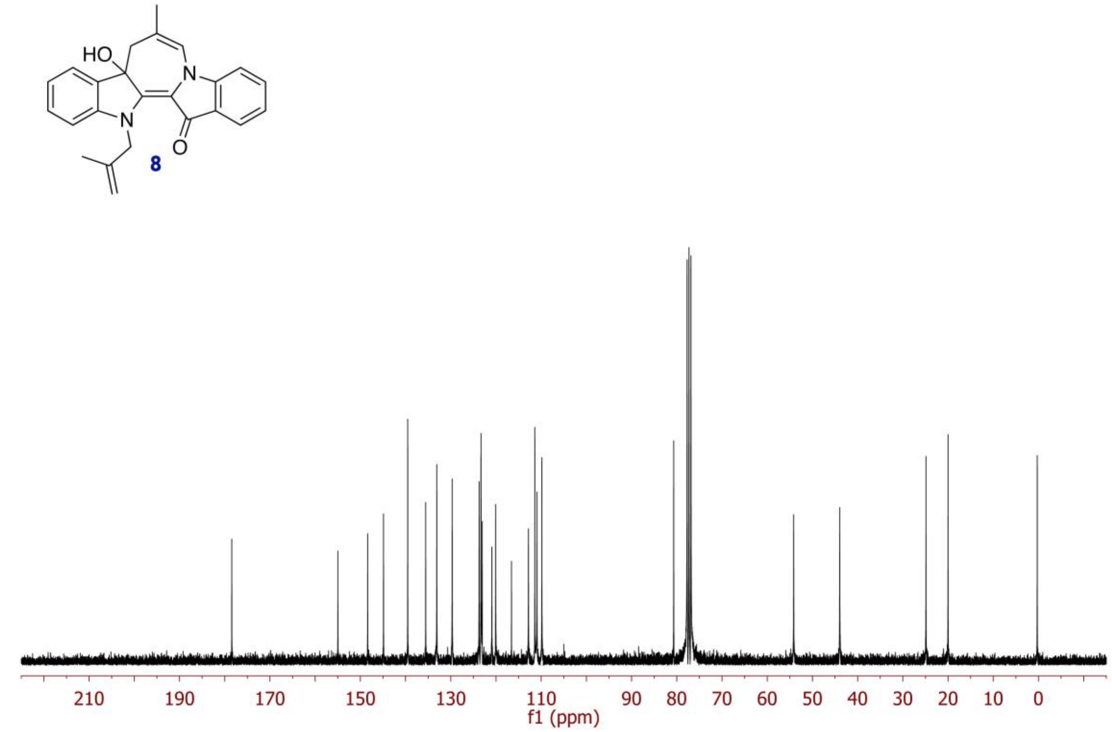

Figure S17: ^{13}C NMR spectrum, recorded in $CDCl_3$ for 8a-hydroxy-7-methyl-13-(2-methylallyl)-8,13a-dihydroazepino[1,2-*a*:3,4-*b'*]diindol-14(8*H*)-one **(8)**

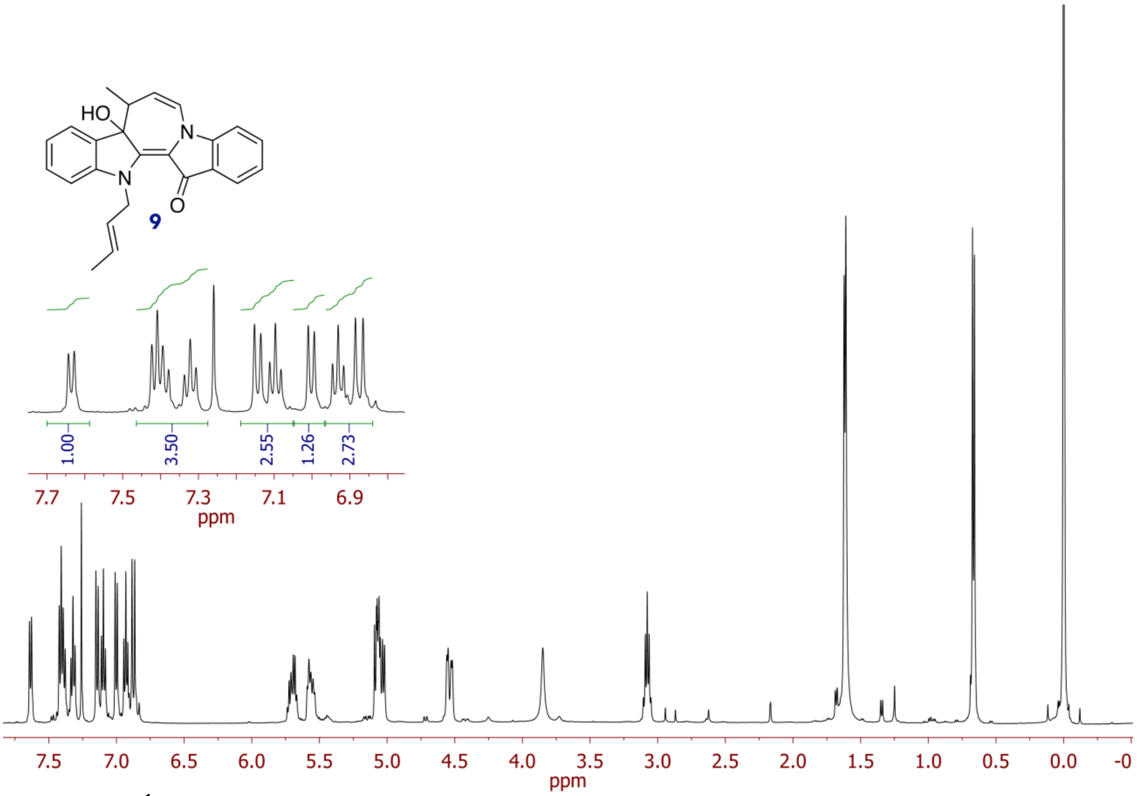

Figure S18: ^{1}H NMR spectrum, recorded in $CDCl_3$ for (*E*)-13-(but-2-en-1-yl)-8a-hydroxy-8-methyl-8,13a-dihydroazepino[1,2-*a*:3,4-*b*']diindol-14(8*H*)-one **(9)**

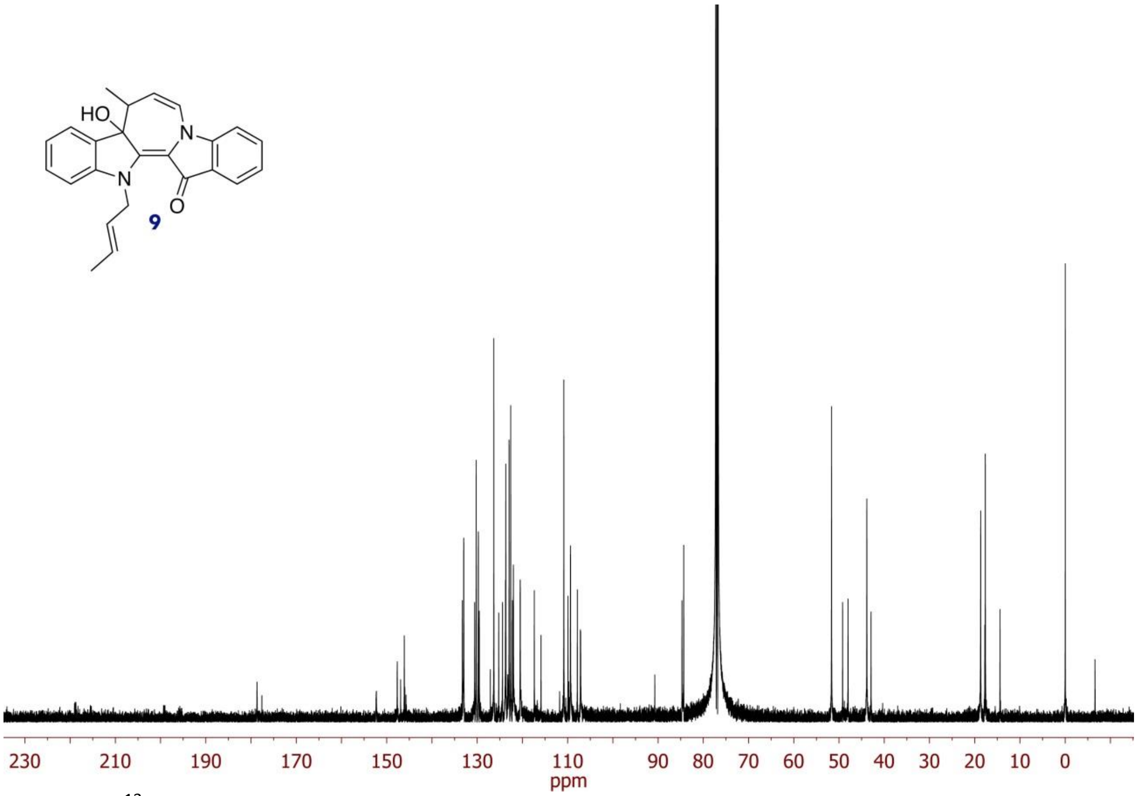

Figure S19: ^{13}C NMR spectrum, recorded in $CDCl_3$ for (*E*)-13-(but-2-en-1-yl)-8a-hydroxy-8-methyl-8,13a-dihydroazepino[1,2-*a*:3,4-*b*']diindol-14(8*H*)-one **(9)**

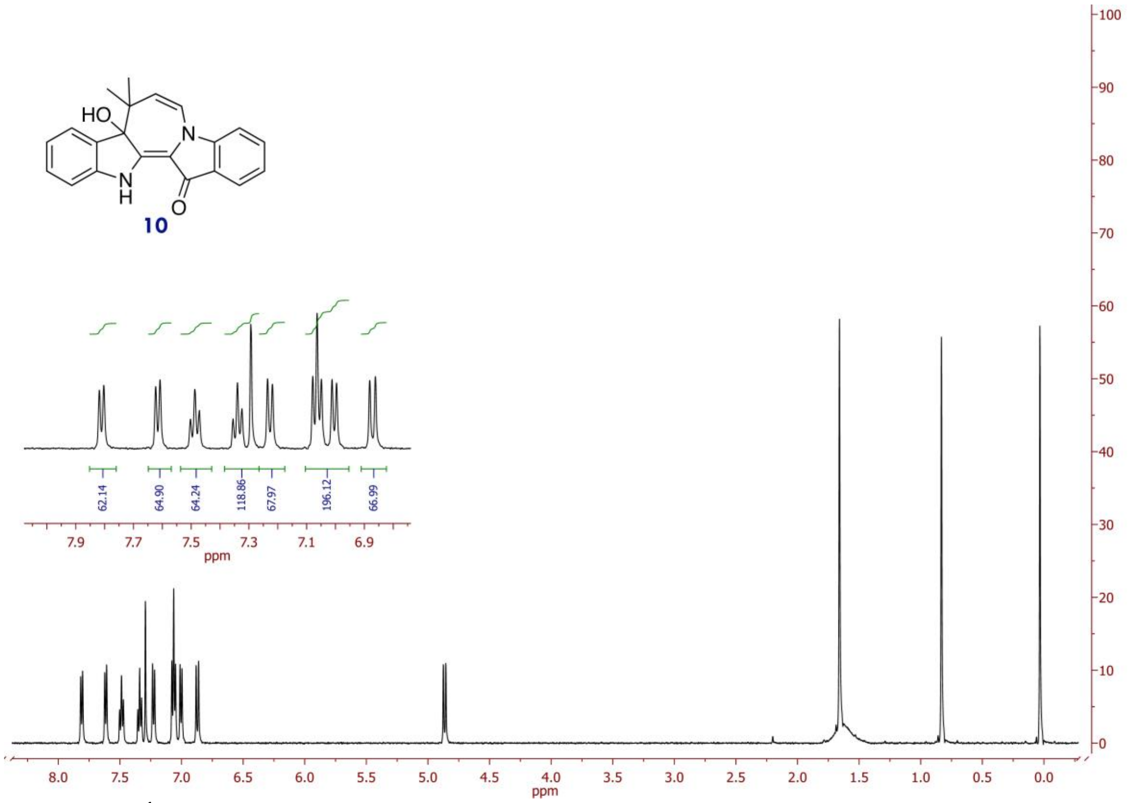

Figure S20: ^{1}H NMR spectrum, recorded in $CDCl_3$ for 8a-hydroxy-8,8-dimethyl-8,13a-dihydroazepino[1,2-*a*:3,4-*b*′]diindol-14(8*H*)-one **(10)**

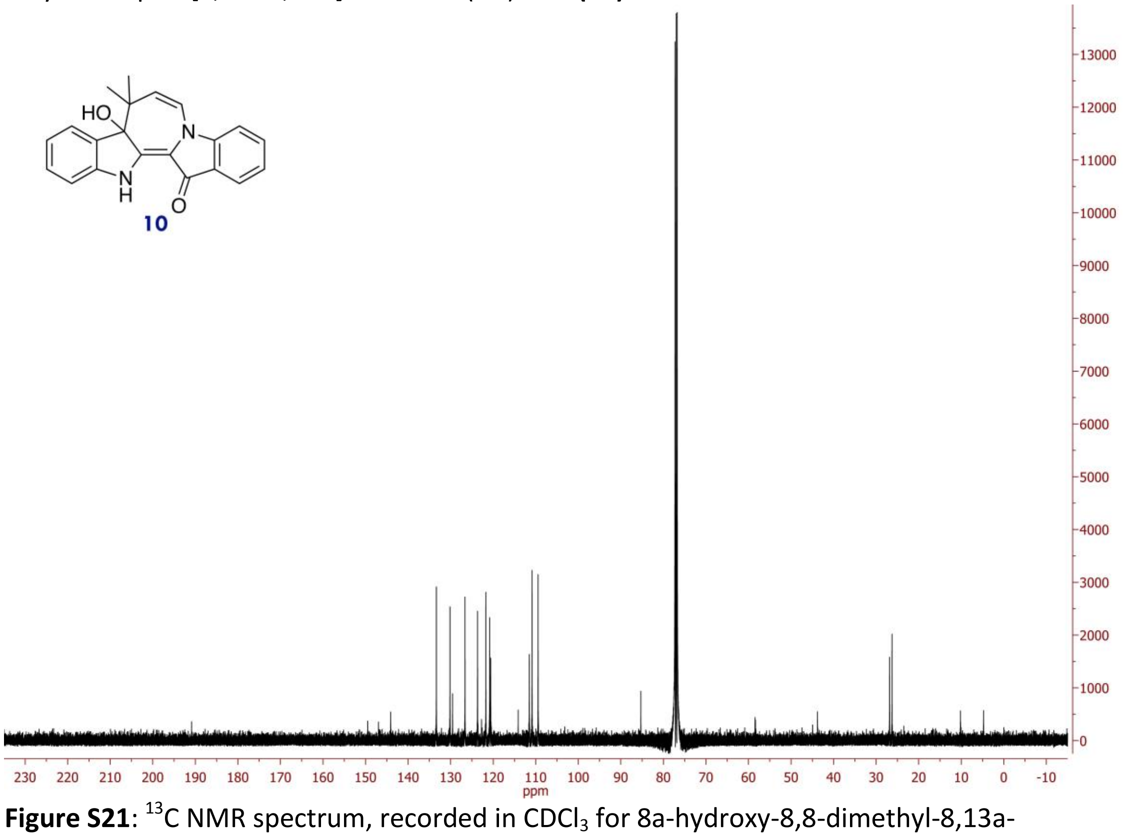

Figure S21: ^{13}C NMR spectrum, recorded in $CDCl_3$ for 8a-hydroxy-8,8-dimethyl-8,13a-dihydroazepino[1,2-*a*:3,4-*b*′]diindol-14(8*H*)-one **(10)**

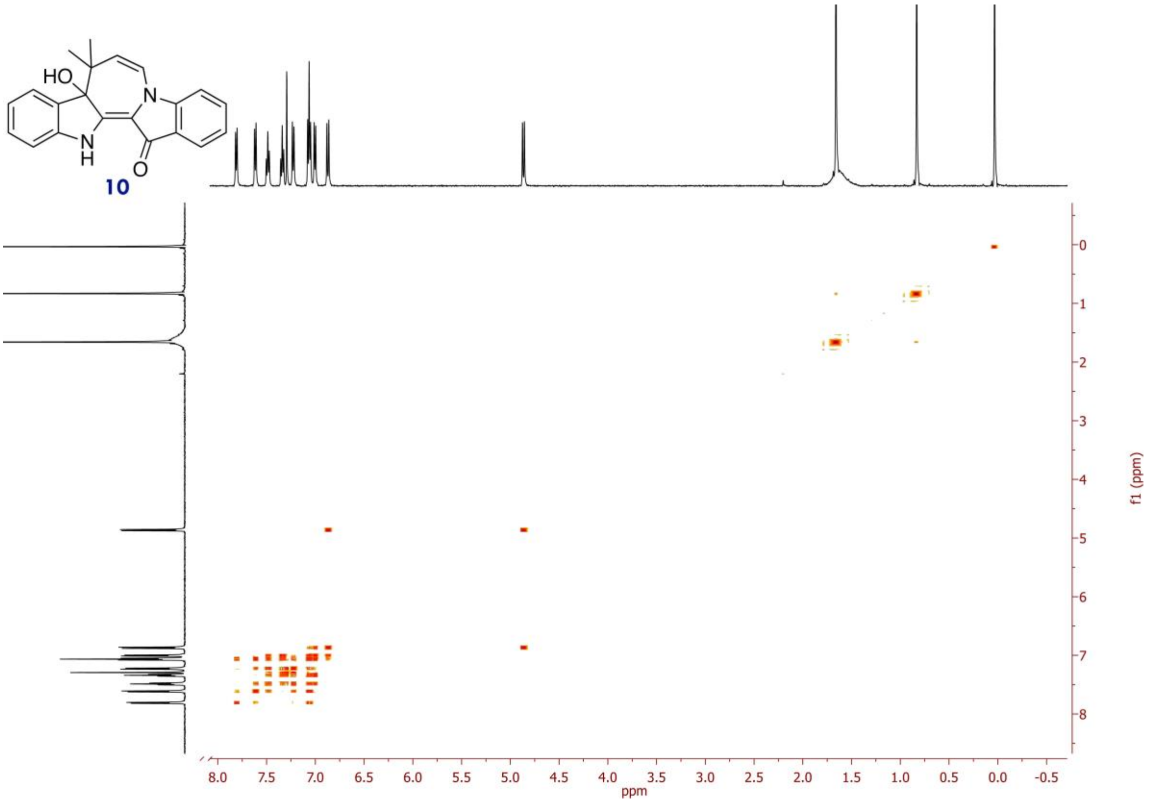

Figure S22: COSY NMR spectrum, recorded in $CDCl_3$ for 8a-hydroxy-8,8-dimethyl-8,13a-dihydroazepino[1,2-*a*:3,4-*b'*]diindol-14(8*H*)-one **(10)**

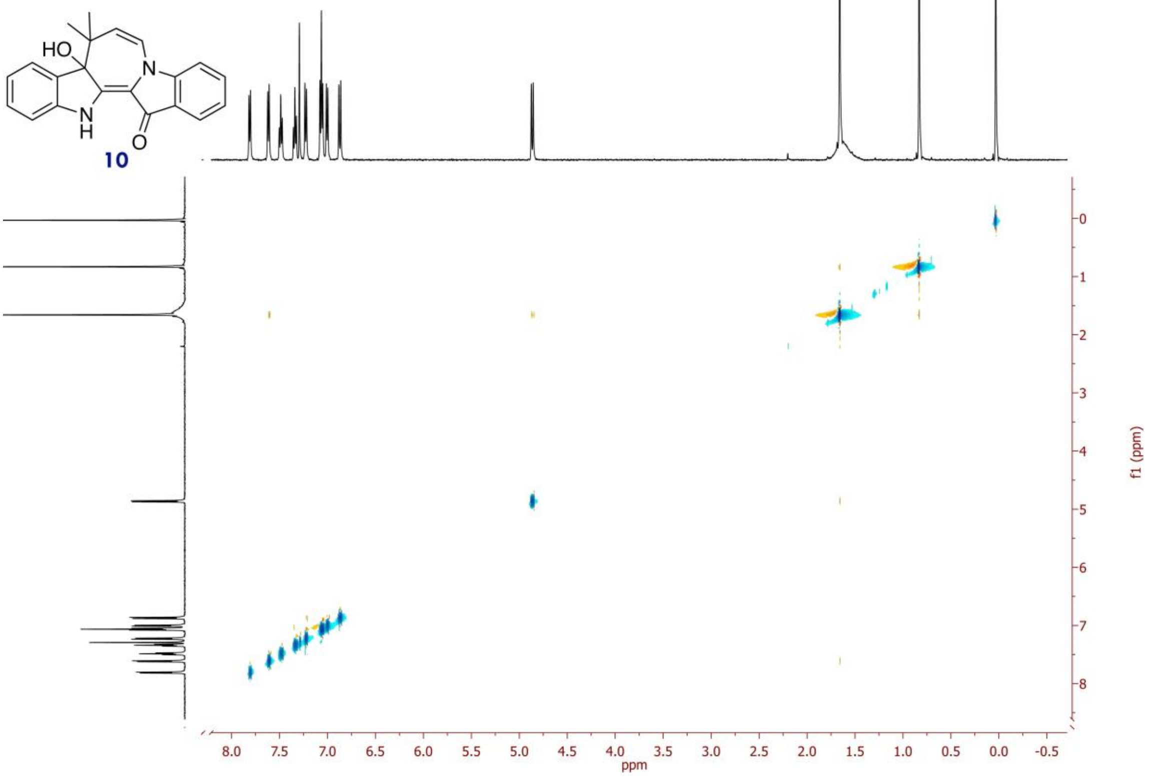

Figure S23: NOESY NMR spectrum, recorded in $CDCl_3$ for 8a-hydroxy-8,8-dimethyl-8,13a-dihydroazepino[1,2-*a*:3,4-*b'*]diindol-14(8*H*)-one **(10)**

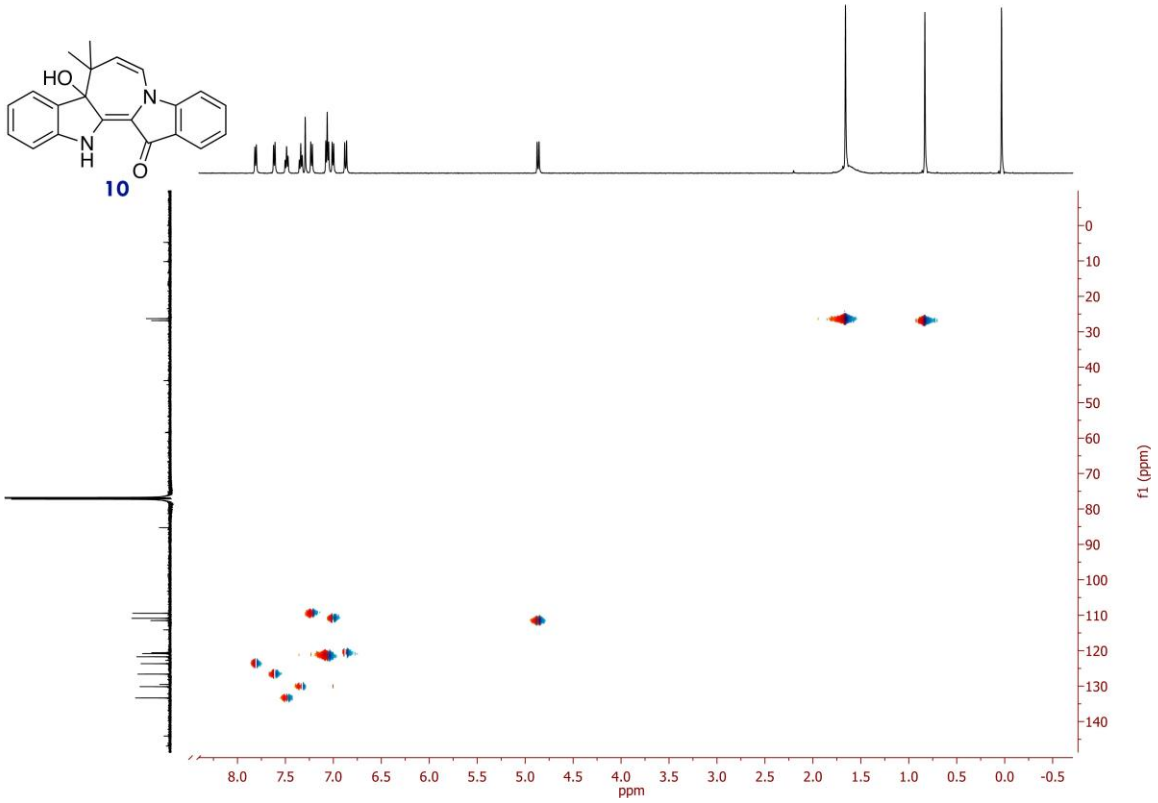

Figure S24: HSQC NMR spectrum, recorded in $CDCl_3$ for 8a-hydroxy-8,8-dimethyl-8,13a-dihydroazepino[1,2-*a:*3,4-*b*′]diindol-14(8*H*)-one **(10)**

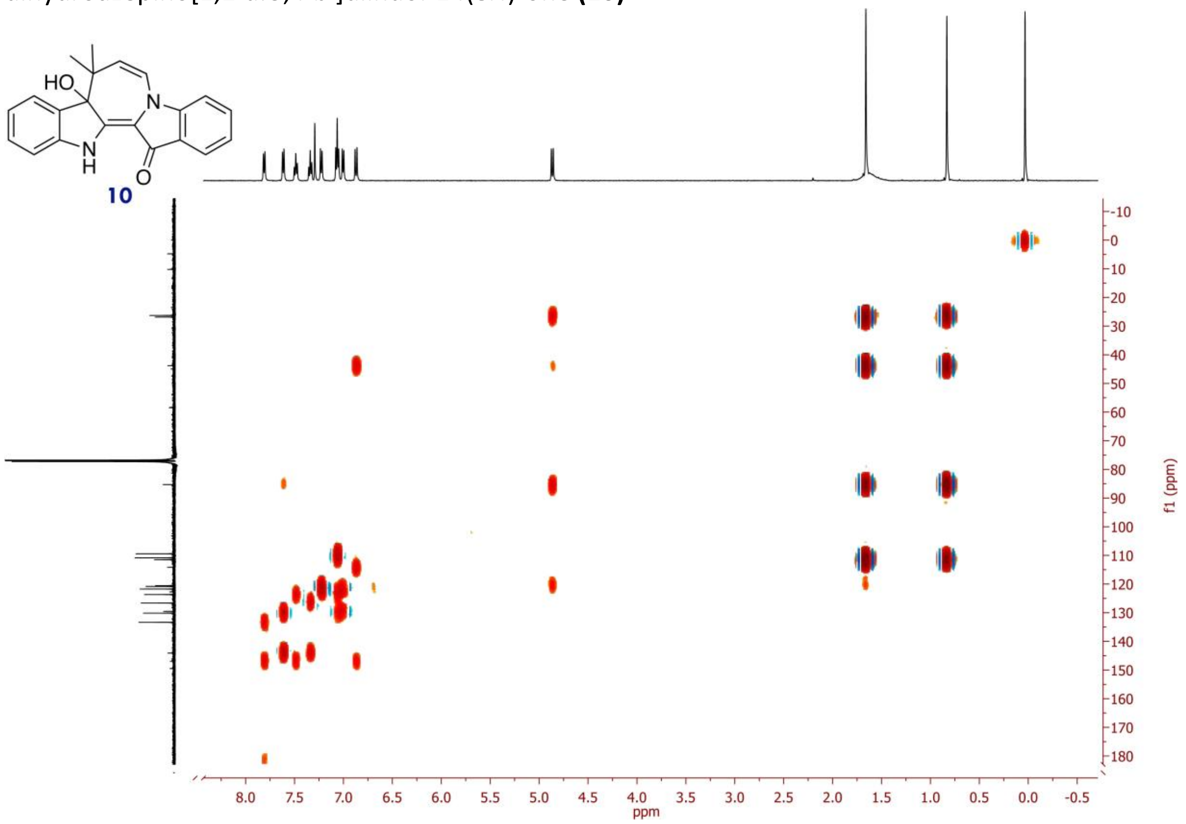

Figure S25: HMBC NMR spectrum, recorded in $CDCl_3$ for 8a-hydroxy-8,8-dimethyl-8,13a-dihydroazepino[1,2-*a*:3,4-*b*′]diindol-14(8*H*)-one **(10)**

Spiro compound from the 3 h reaction

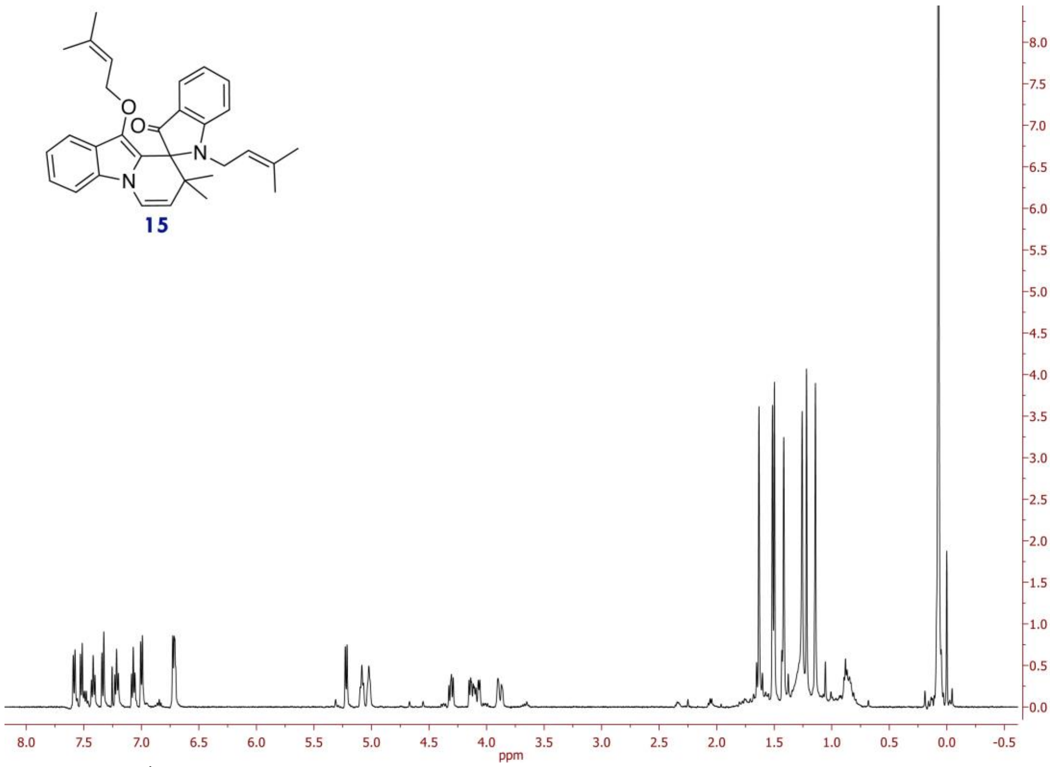

Figure S26: ^{1}H NMR spectrum, recorded in $CDCl_3$ for 8',8'-dimethyl-1-(3-methylbut-2-en-1-yl)-10'-((3-methylbut-2-en-1-yl)oxy)-8'*H*-spiro[indoline-2,9'-pyrido[1,2-*a*]indol]-3-one **(15)**

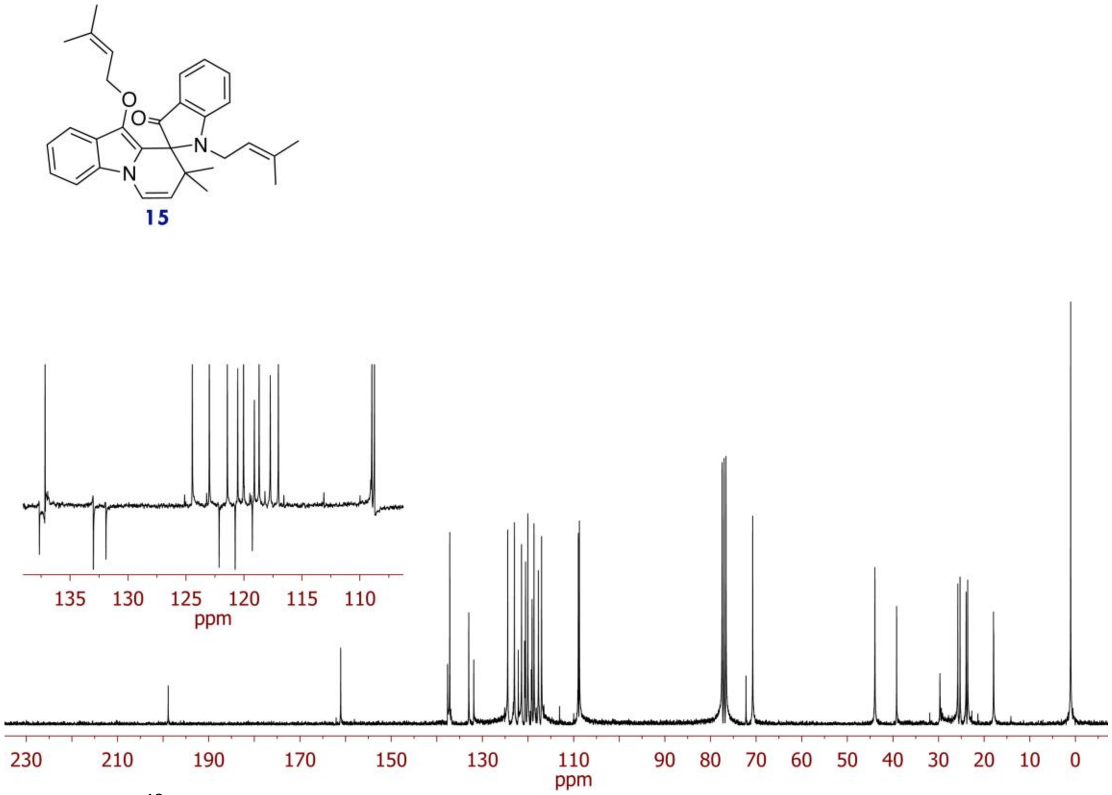

Figure S27: ^{13}C NMR spectrum, recorded in $CDCl_3$ for 8',8'-dimethyl-1-(3-methylbut-2-en-1-yl)-10'-((3-methylbut-2-en-1-yl)oxy)-8'*H*-spiro[indoline-2,9'-pyrido[1,2-*a*]indol]-3-one **(15)**

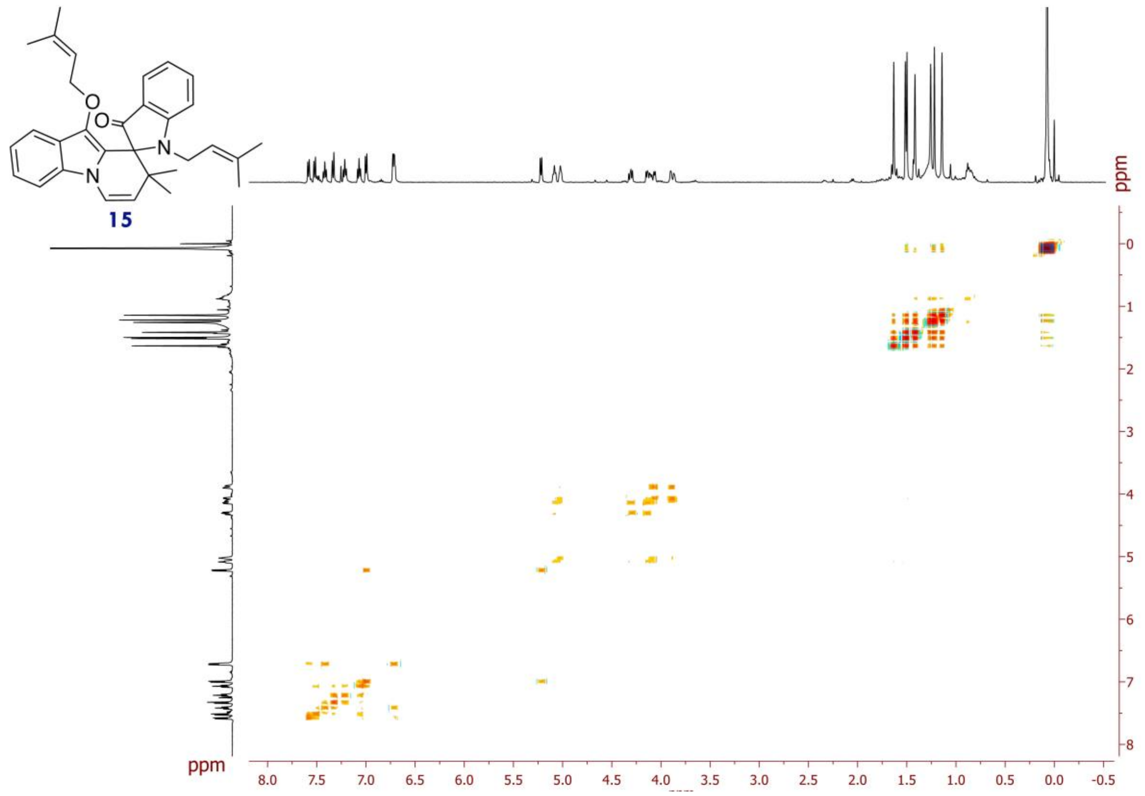

Figure S28: COSY NMR spectrum, recorded in $CDCl_3$ for 8',8'-dimethyl-1-(3-methylbut-2-en-1-yl)-10'-((3-methylbut-2-en-1-yl)oxy)-8'*H*-spiro[indoline-2,9'-pyrido[1,2-*a*]indol]-3-one **(15)**

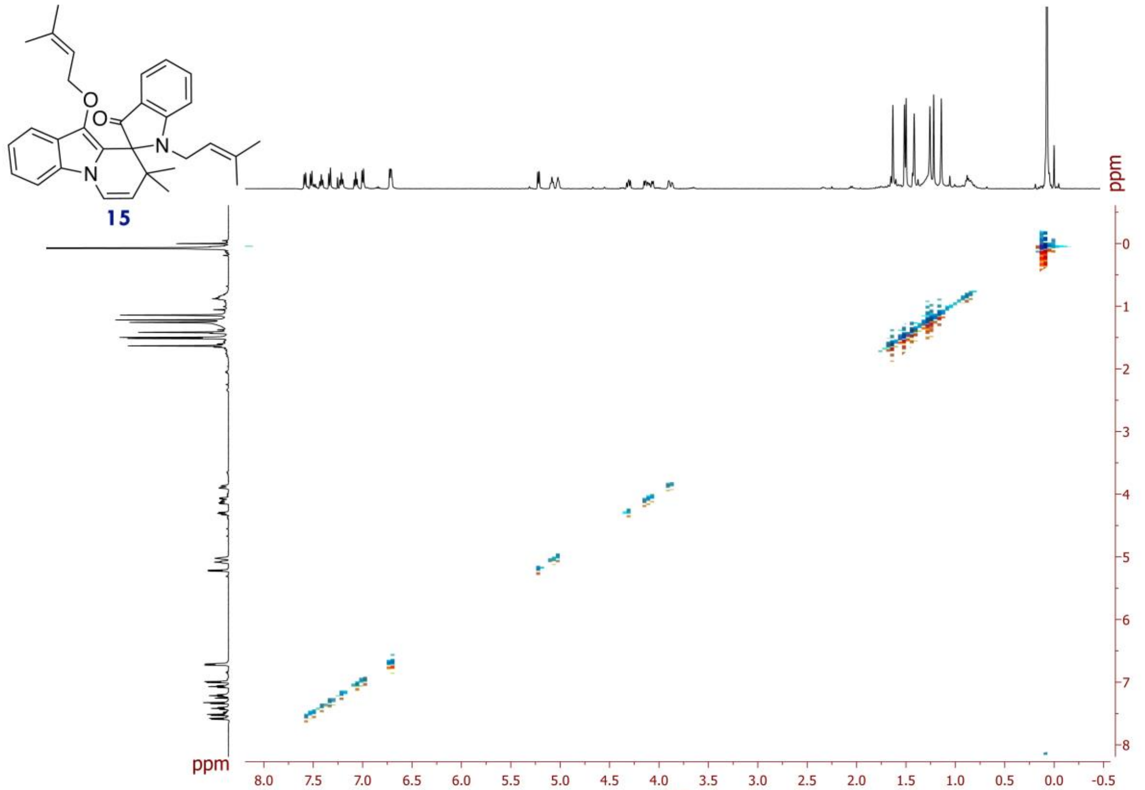

Figure S29: NOSY NMR spectrum, recorded in $CDCl_3$ for 8',8'-dimethyl-1-(3-methylbut-2-en-1-yl)-10'-((3-methylbut-2-en-1-yl)oxy)-8'*H*-spiro[indoline-2,9'-pyrido[1,2-*a*]indol]-3-one **(15)**

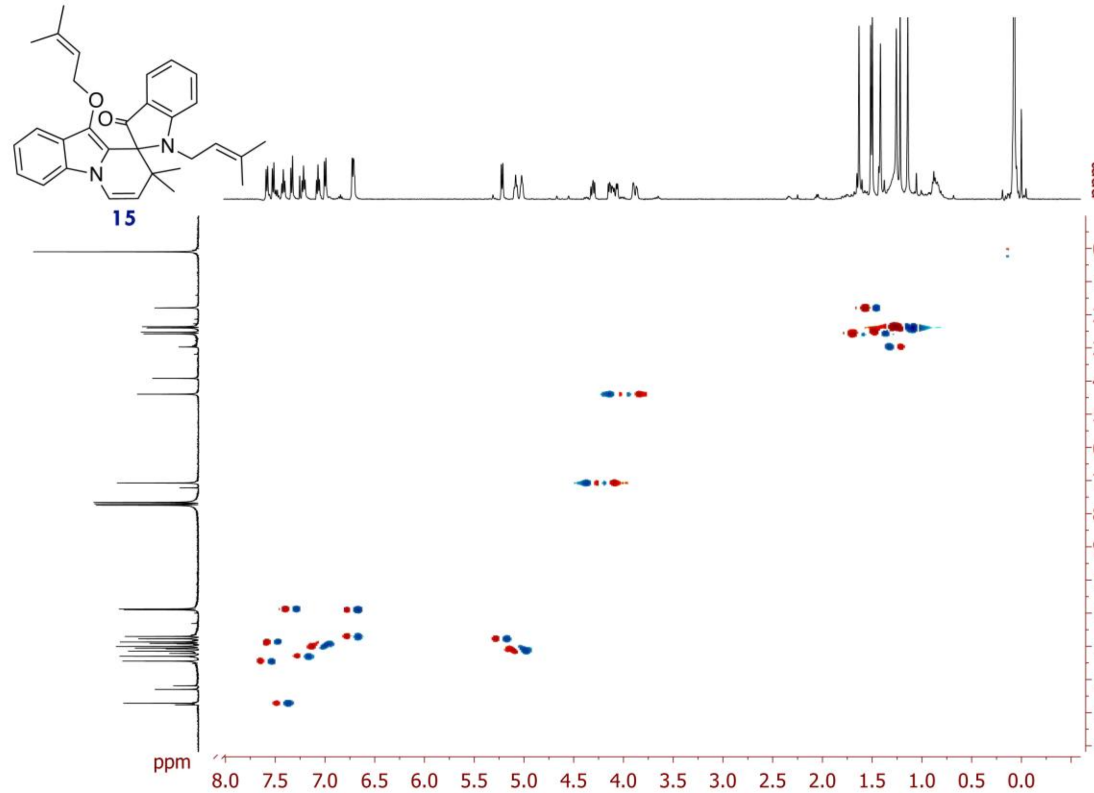

Figure S30: HSQC NMR spectrum, recorded in $CDCl_3$ for 8',8'-dimethyl-1-(3-methylbut-2-en-1-yl)-10'-((3-methylbut-2-en-1-yl)oxy)-8'*H*-spiro[indoline-2,9'-pyrido[1,2-*a*]indol]-3-one **(15)**

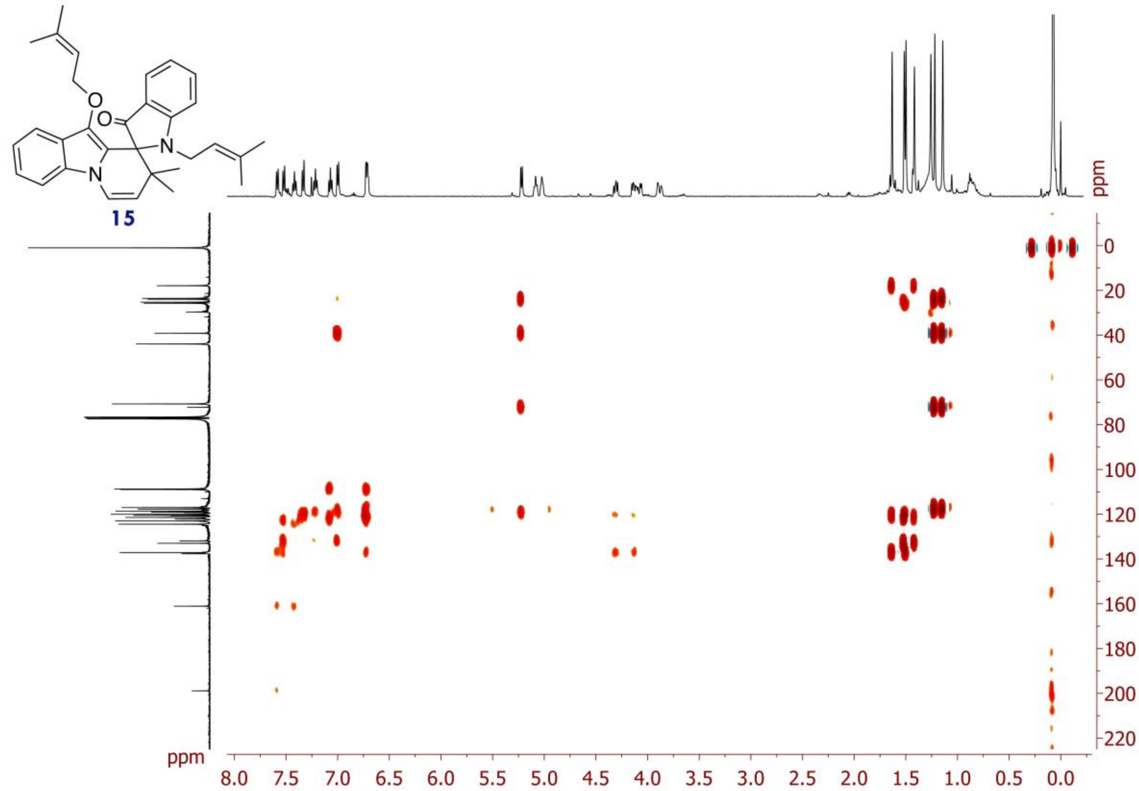

Figure S31: HMBC NMR spectrum, recorded in $CDCl_3$ for 8',8'-dimethyl-1-(3-methylbut-2-en-1-yl)-10'-((3-methylbut-2-en-1-yl)oxy)-8'*H*-spiro[indoline-2,9'-pyrido[1,2-*a*]indol]-3-one **(15)**

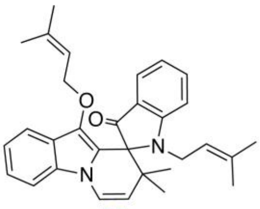

15

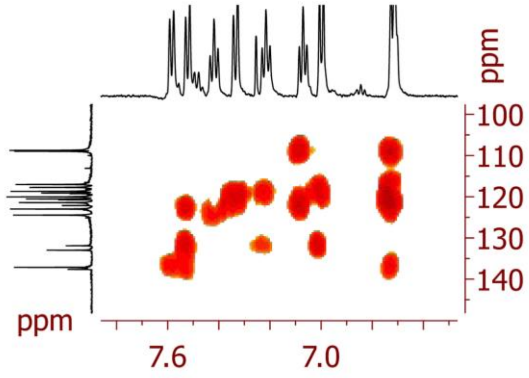

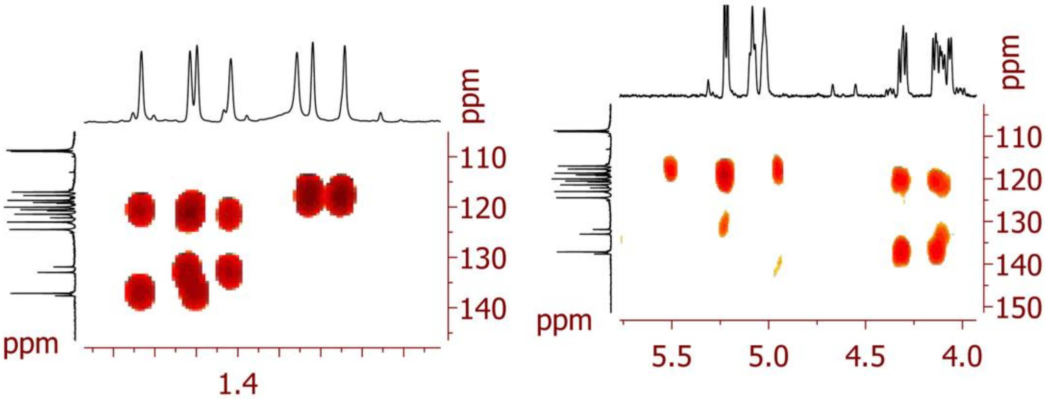

Figure S32: HMBC NMR spectrum expansion, recorded in $CDCl_3$ for 8',8'-dimethyl-1-(3-methylbut-2-en-1-yl)-10'-((3-methylbut-2-en-1-yl)oxy)-8'*H*-spiro[indoline-2,9'-pyrido[1,2-*a*]indol]-3-one **(15)**

Oxa-bridged Azepinodiindolone

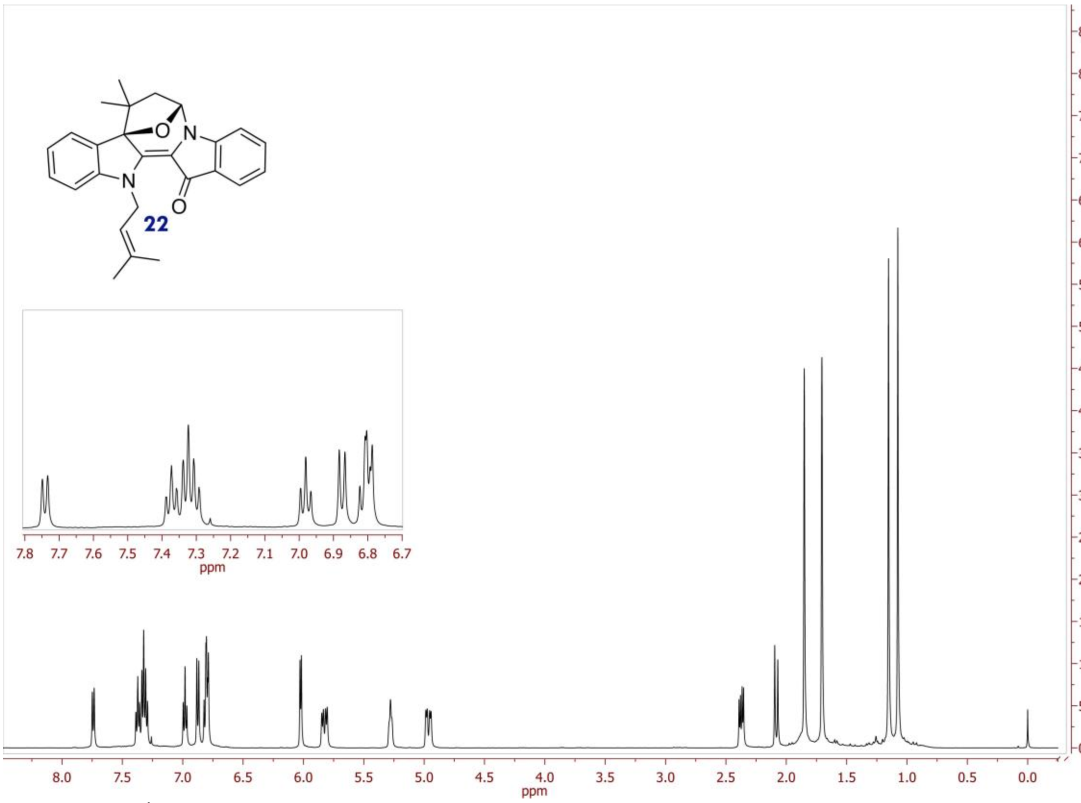

Figure S33: ^{1}H NMR spectrum, recorded in $CDCl_3$ for 8,8-dimethyl-13-(3-methylbut-2-en-1-yl)-7,8-dihydro-6*H*-6,8a-epoxyazepino[1,2-*a*:3,4-*b'*]diindol-14(13*H*)-one **(22)**

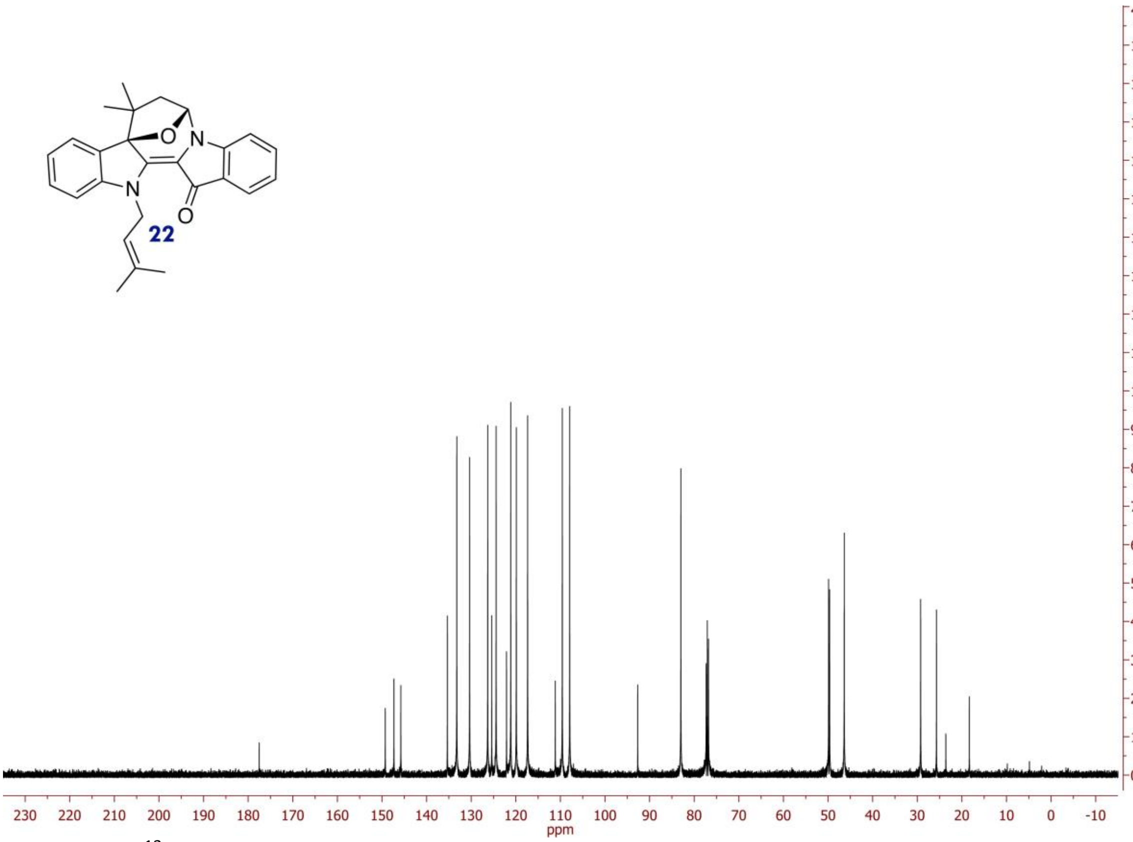

Figure S34: ^{13}C NMR spectrum, recorded in $CDCl_3$ for 8,8-dimethyl-13-(3-methylbut-2-en-1-yl)-7,8-dihydro-6*H*-6,8a-epoxyazepino[1,2-*a*:3,4-*b'*]diindol-14(13*H*)-one **(22)**

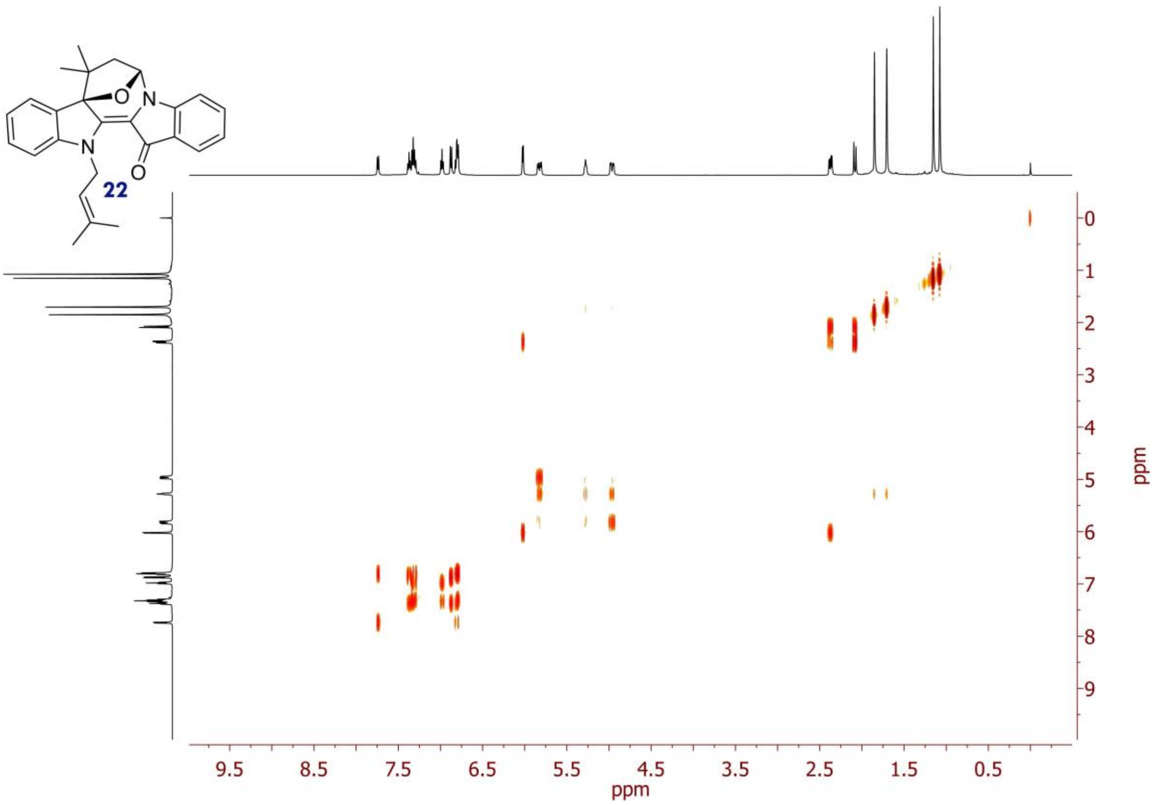

Figure S35: COSY NMR spectrum, recorded in $CDCl_3$ for 8,8-dimethyl-13-(3-methylbut-2-en-1-yl)-7,8-dihydro-6*H*-6,8a-epoxyazepino[1,2-*a*:3,4-*b'*]diindol-14(13*H*)-one **(22)**

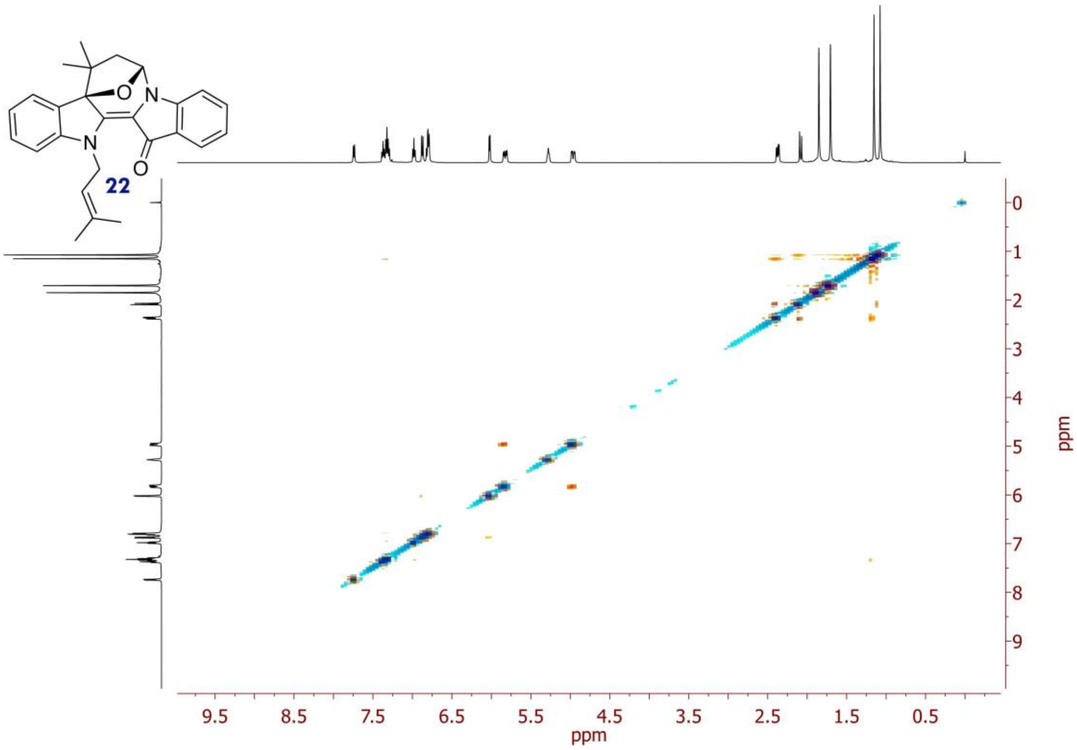

Figure S36: NOESY NMR spectrum, recorded in $CDCl_3$ for 8,8-dimethyl-13-(3-methylbut-2-en-1-yl)-7,8-dihydro-6*H*-6,8a-epoxyazepino[1,2-*a*:3,4-*b'*]diindol-14(13*H*)-one **(22)**

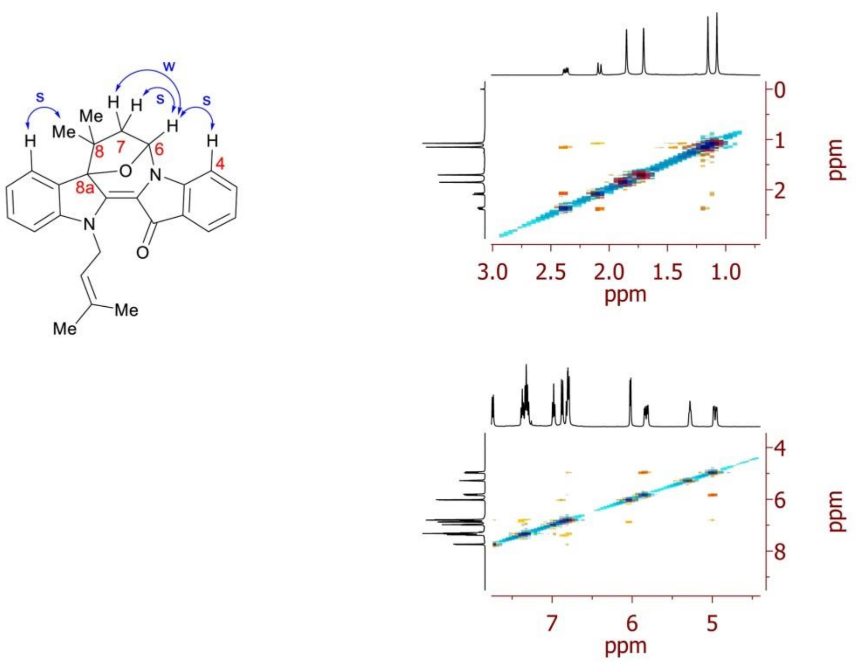

Figure S37: NOESY NMR spectrum expansion and crucial correlations of compound **(22)**

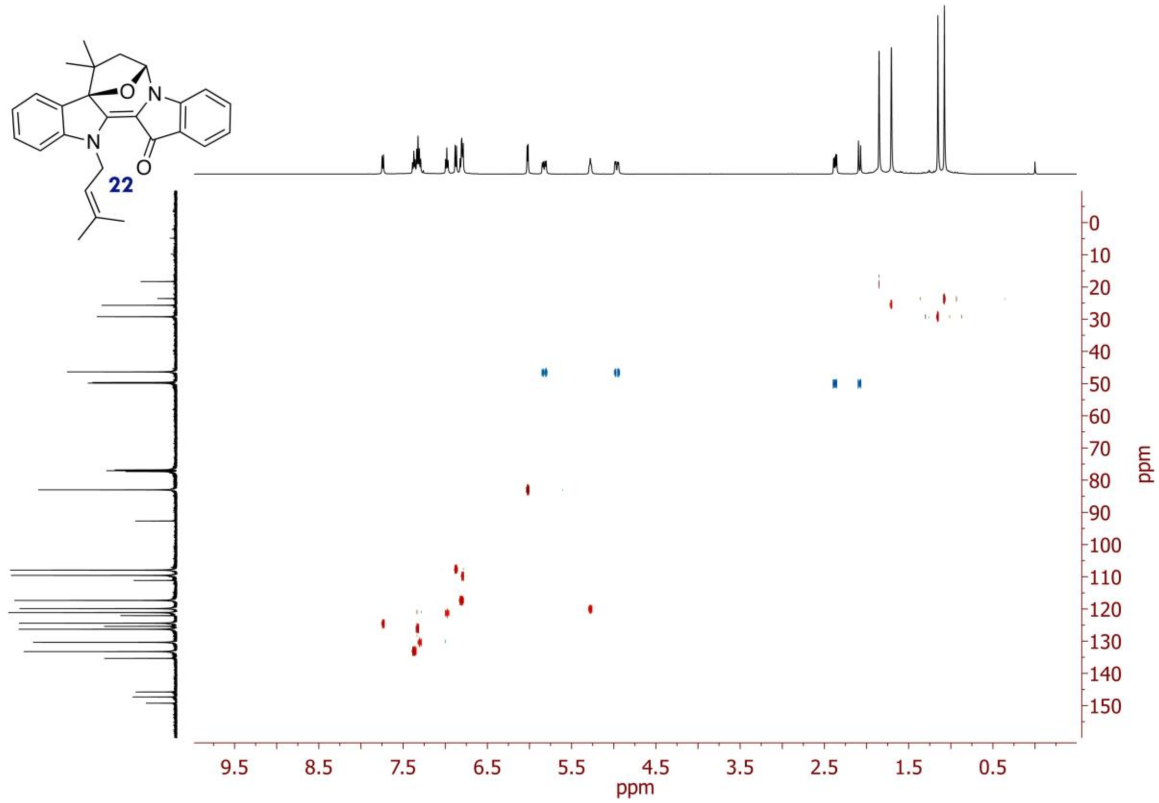

Figure S38: HSQC NMR spectrum, recorded in $CDCl_3$ for 8,8-dimethyl-13-(3-methylbut-2-en-1-yl)-7,8-dihydro-6*H*-6,8a-epoxyazepino[1,2-*a*:3,4-*b'*]diindol-14(13*H*)-one **(22)**

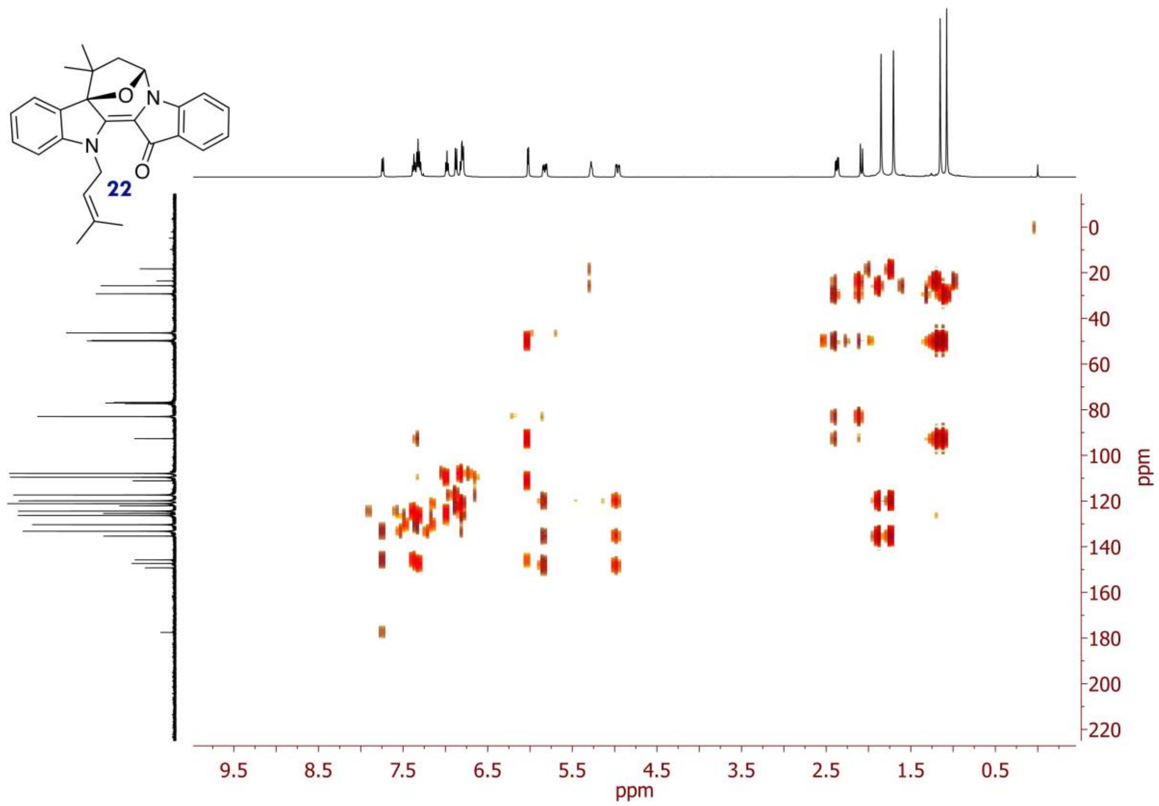

Figure S39: HMBC NMR spectrum, recorded in $CDCl_3$ for 8,8-dimethyl-13-(3-methylbut-2-en-1-yl)-7,8-dihydro-6*H*-6,8a-epoxyazepino[1,2-*a*:3,4-*b'*]diindol-14(13*H*)-one **(22)**

***C*-Alkylated Spiro product**

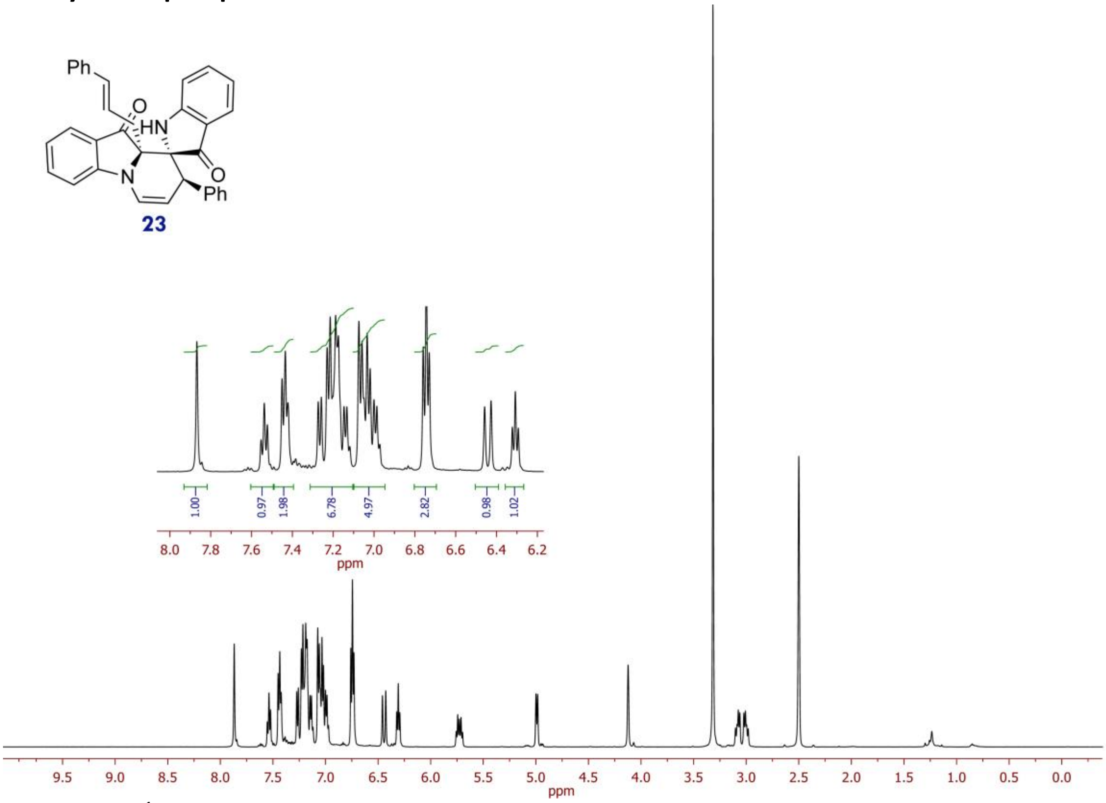

Figure S40: ^{1}H NMR spectrum, recorded in $(CD_3)_2SO$ for (2*R*,8'*R*,9a'*R*)-9a'-cinnamyl-8'-phenyl-8'*H*-spiro[indoline-2,9'-pyrido[1,2-*a*]indole]-3,10'(9a'*H*)-dione **(23)**

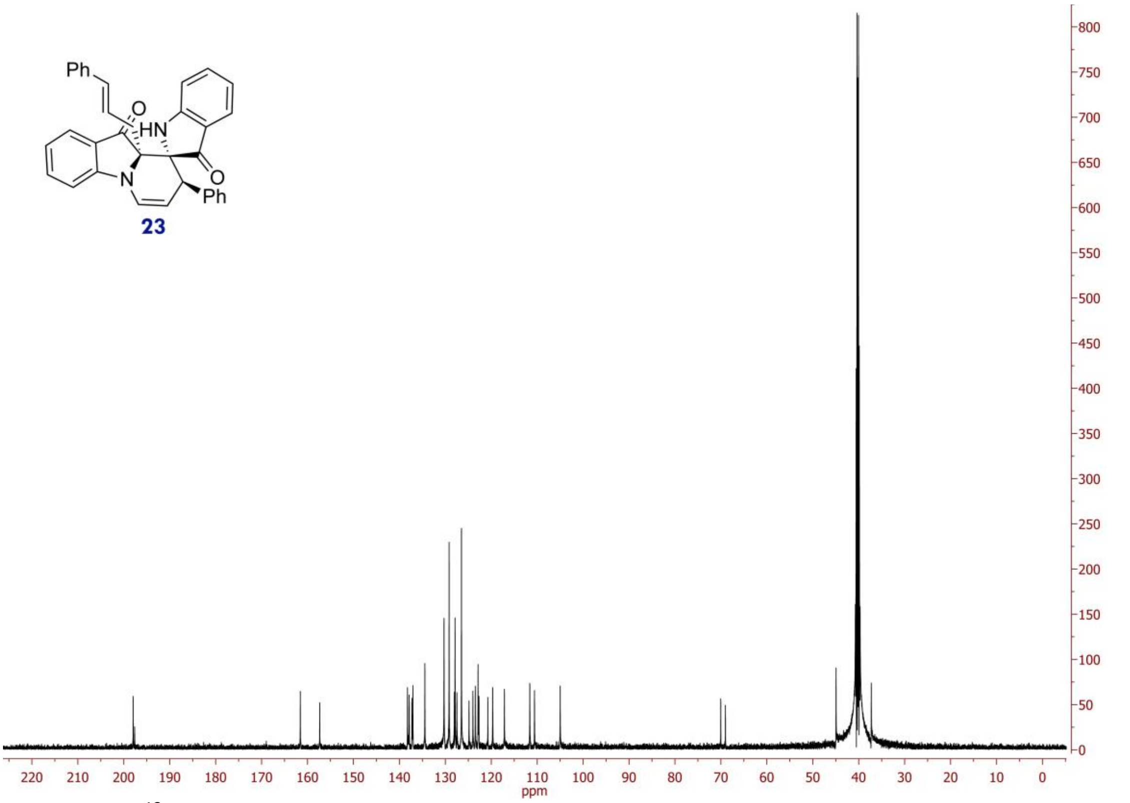

Figure S41: ^{13}C NMR spectrum, recorded in $(CD_3)_2SO$ for (2*R*,8'*R*,9a'*R*)-9a'-cinnamyl-8'-phenyl-8'*H*-spiro[indoline-2,9'-pyrido[1,2-*a*]indole]-3,10'(9a'*H*)-dione **(23)**

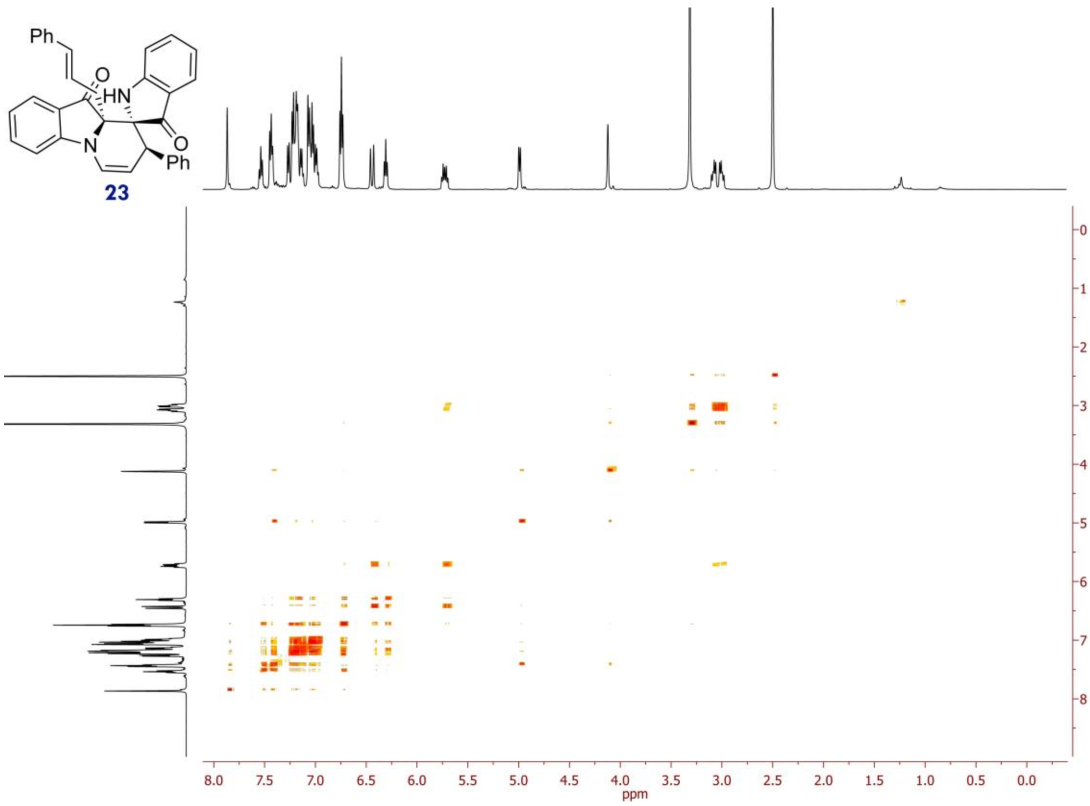

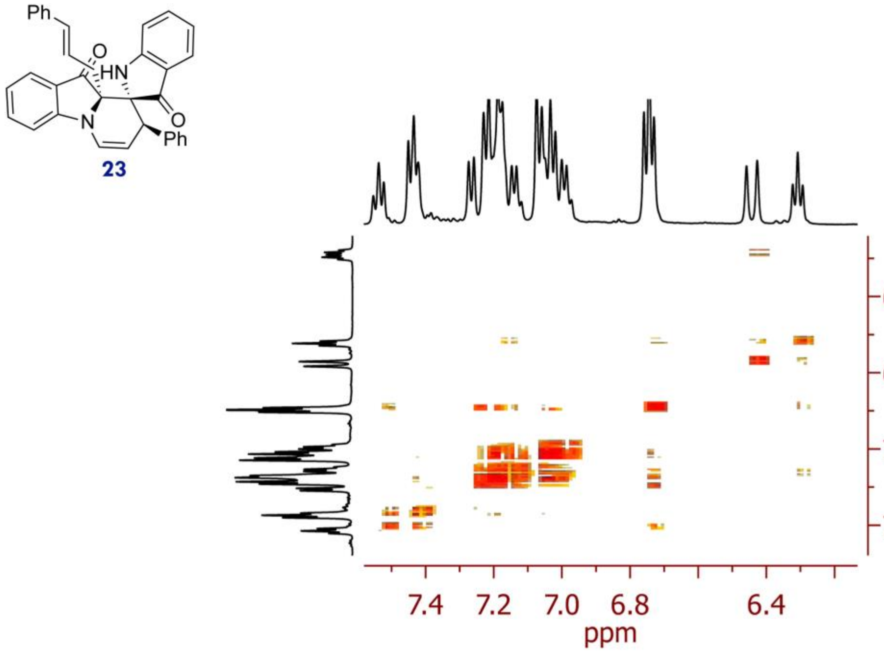

Figure S42: COSY NMR spectrum and the corresponding expansion, recorded in $(CD_3)_2SO$ for (2*R*,8'*R*,9a'*R*)-9a'-cinnamyl-8'-phenyl-8'*H*-spiro[indoline-2,9'-pyrido[1,2-*a*]indole]-3,10'(9a'*H*)-dione **(23)**

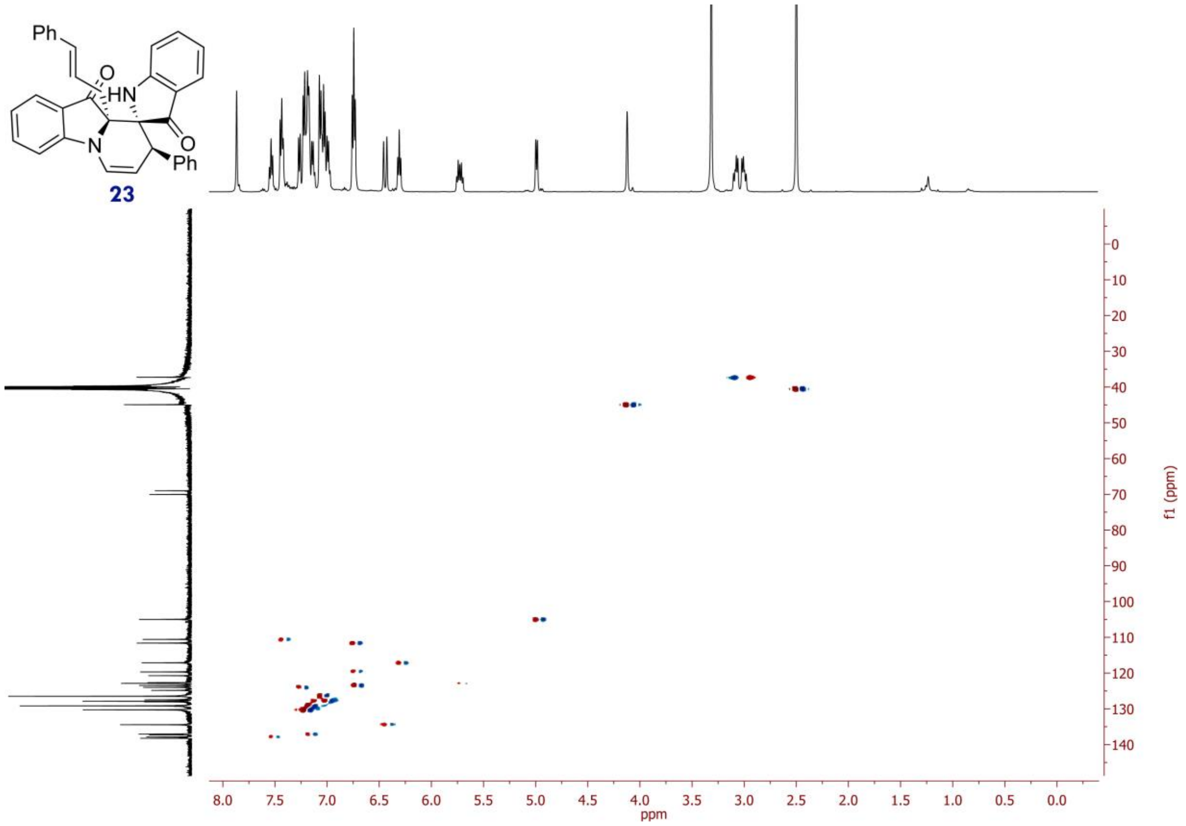

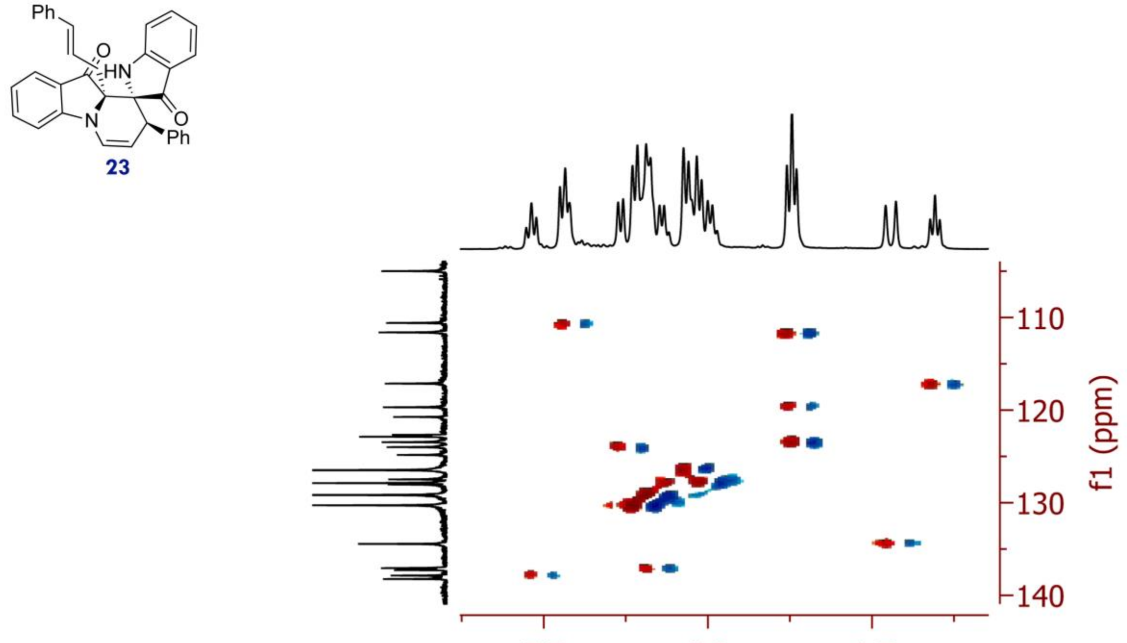

Figure S43: HSQC NMR spectrum and the corresponding expansion, recorded in $(CD_3)_2SO$ for (2*R*,8'*R*,9a'*R*)-9a'-cinnamyl-8'-phenyl-8'*H*-spiro[indoline-2,9'-pyrido[1,2-*a*]indole]-3,10'(9a'*H*)-dione **(23)**

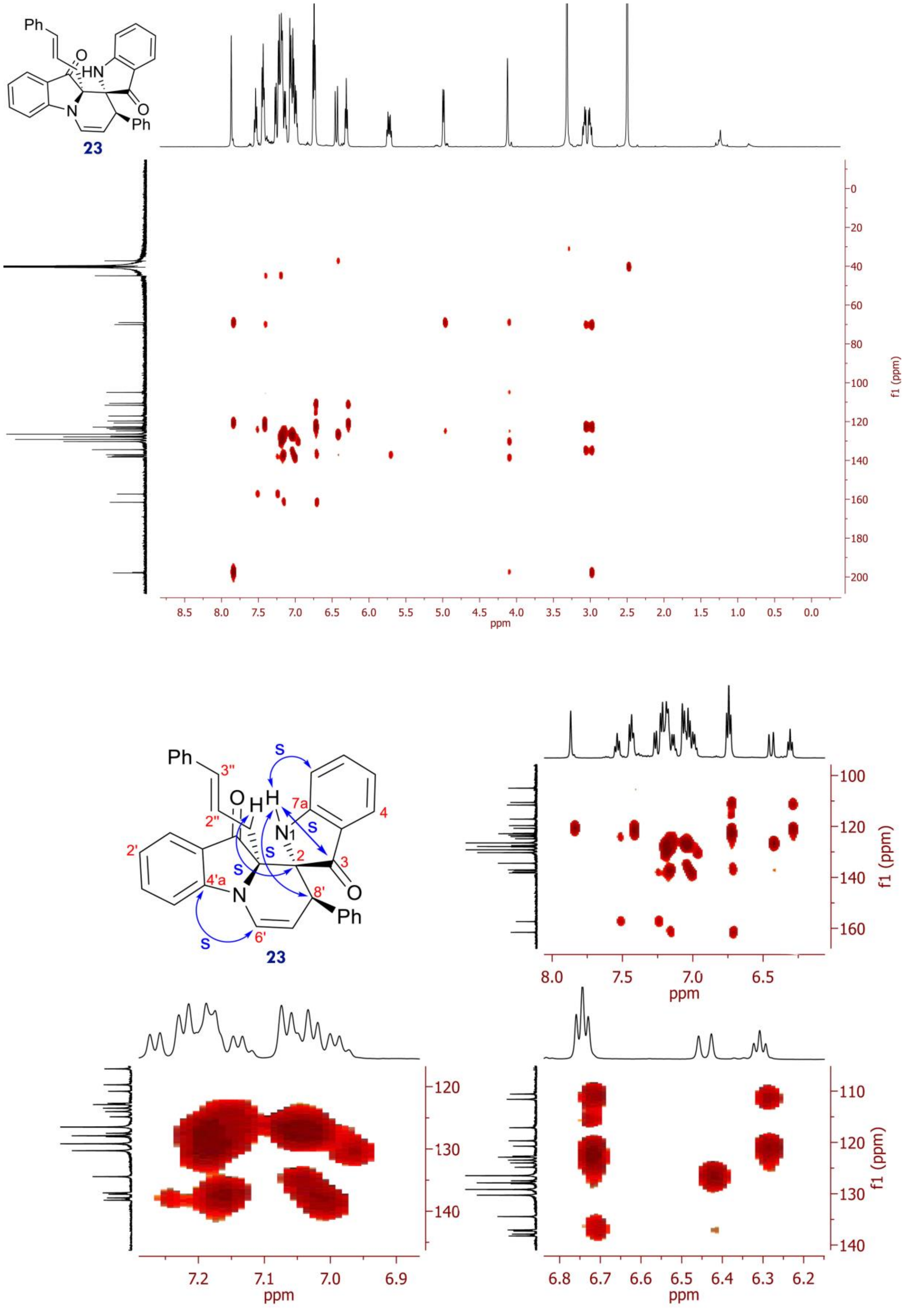

Figure S44: HMBC NMR spectrum and the corresponding expansion, recorded in $(CD_3)_2SO$ for (2*R*,8'*R*,9a'*R*)-9a'-cinnamyl-8'-phenyl-8'*H*-spiro[indoline-2,9'-pyrido[1,2-*a*]indole]-3,10'(9a'*H*)-dione **(23)**

Ring closing metathesis product

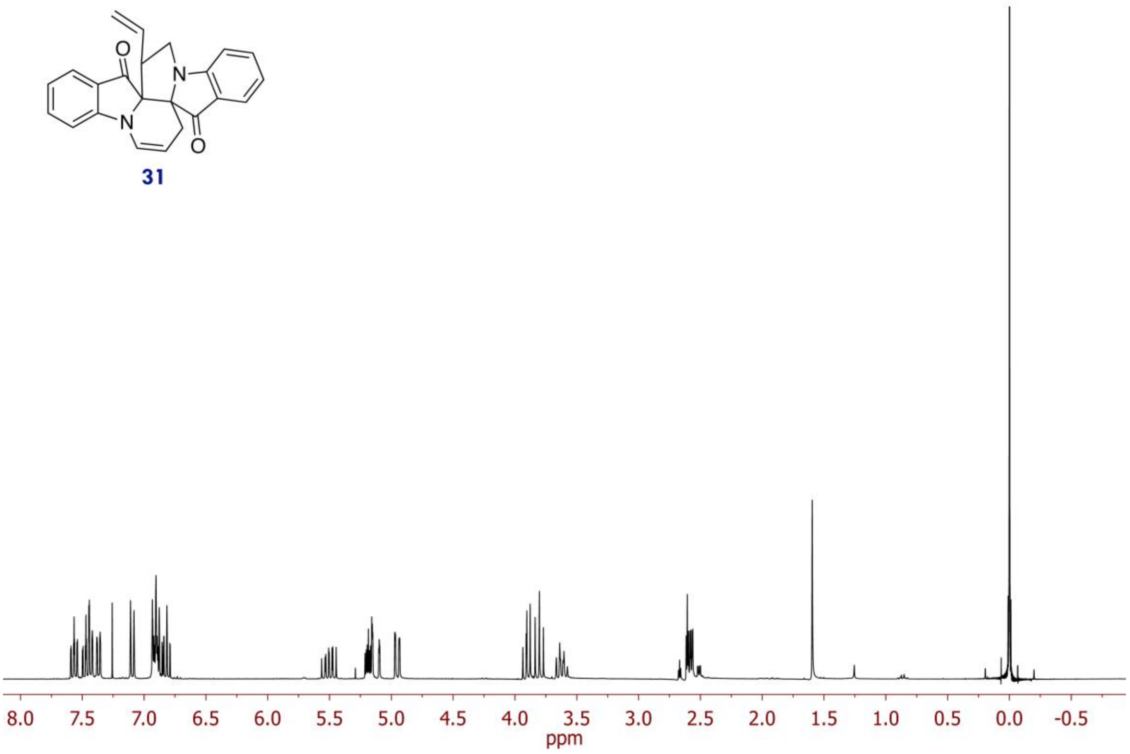

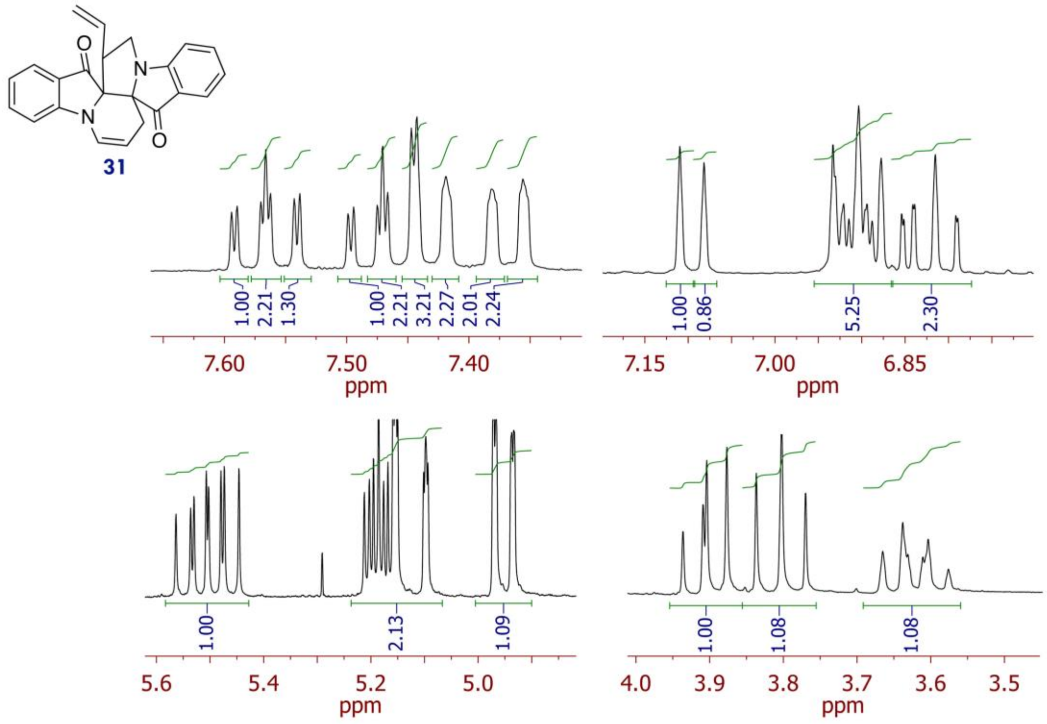

Figure S45: ^{1}H NMR spectrum, recorded in $CDCl_3$ for 1,2-dihydro-1-vinyl-8*H*,9*H*,17*H*-benz[2′,3′]pyrolizino[1′,7′a:2,3]pyrido[1,2-*a*]indole-8.17-dione **(31)**

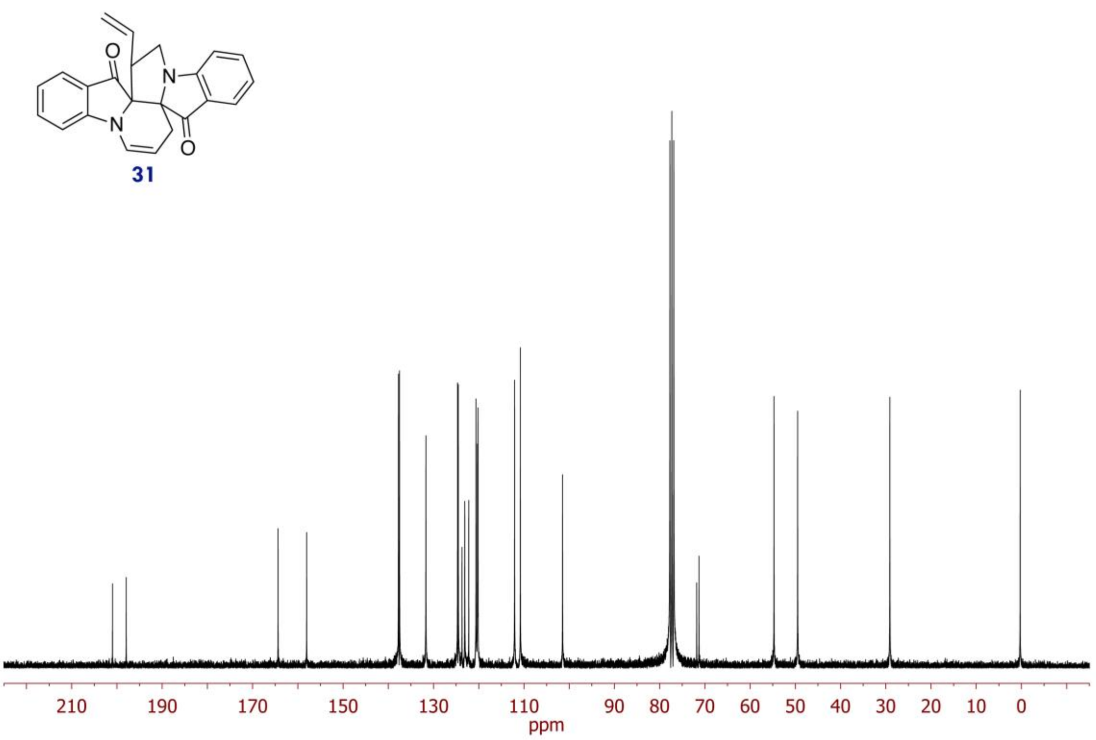

Figure S46: ^{13}C NMR spectrum, recorded in $CDCl_3$ for 1,2-dihydro-1-vinyl-8*H*,9*H*,17*H*-benz[2′,3′]pyrolizino[1′,7′a:2,3]pyrido[1,2-*a*]indole-8.17-dione **(31)**

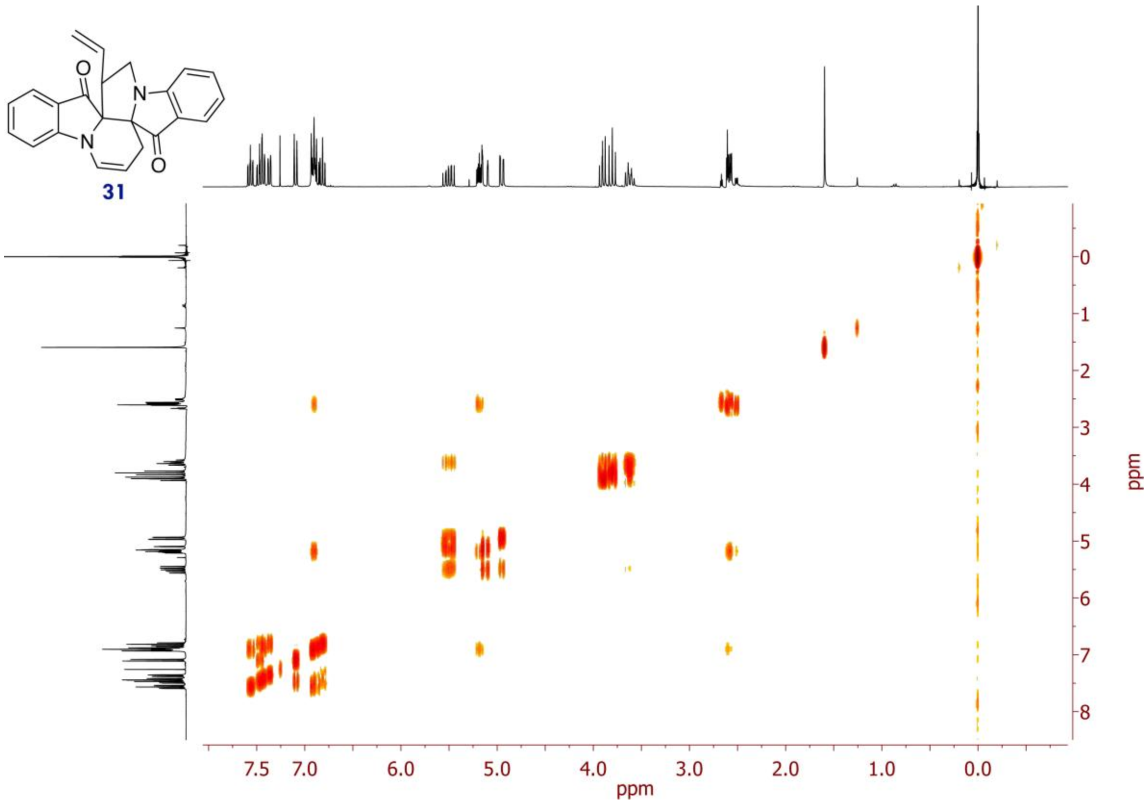

Figure S47: COSY NMR spectrum, recorded in $CDCl_3$ for 1,2-dihydro-1-vinyl-8*H*,9*H*,17*H*-benz[2′,3′]pyrolizino[1′,7′a:2,3]pyrido[1,2-*a*]indole-8.17-dione **(31)**

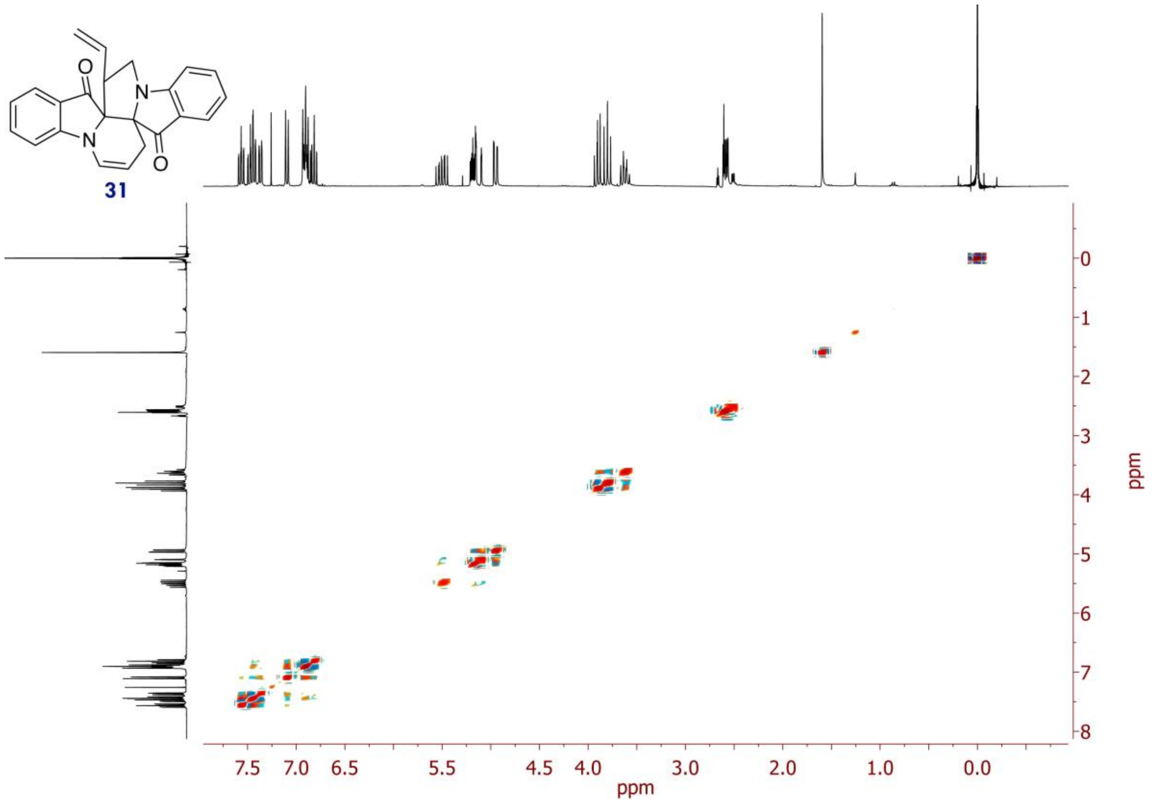

Figure S48: NOSY NMR spectrum, recorded in $CDCl_3$ for 1,2-dihydro-1-vinyl-8*H*,9*H*,17*H*-benz[2′,3′]pyrolizino[1′,7′a:2,3]pyrido[1,2-*a*]indole-8.17-dione **(31)**

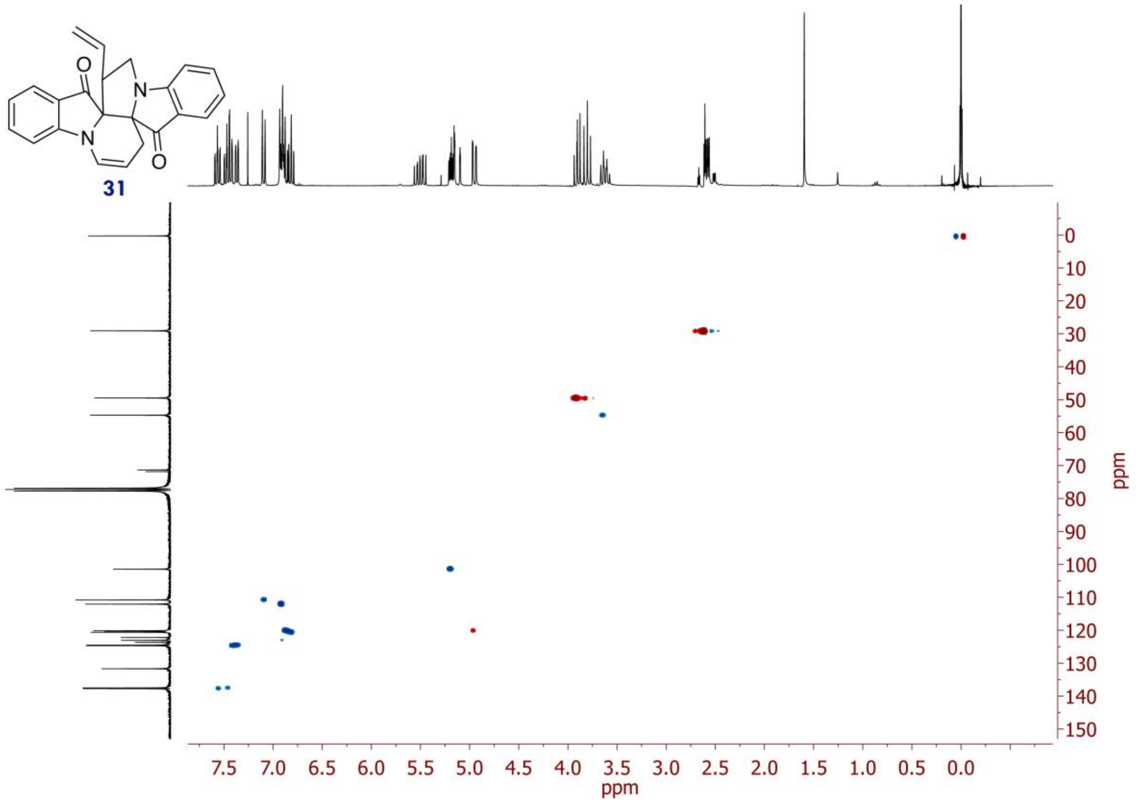

Figure S49: HSQC NMR spectrum, recorded in $CDCl_3$ for 1,2-dihydro-1-vinyl-8*H*,9*H*,17*H*-benz[2′,3′]pyrolizino[1′,7′a:2,3]pyrido[1,2-*a*]indole-8.17-dione **(31)**

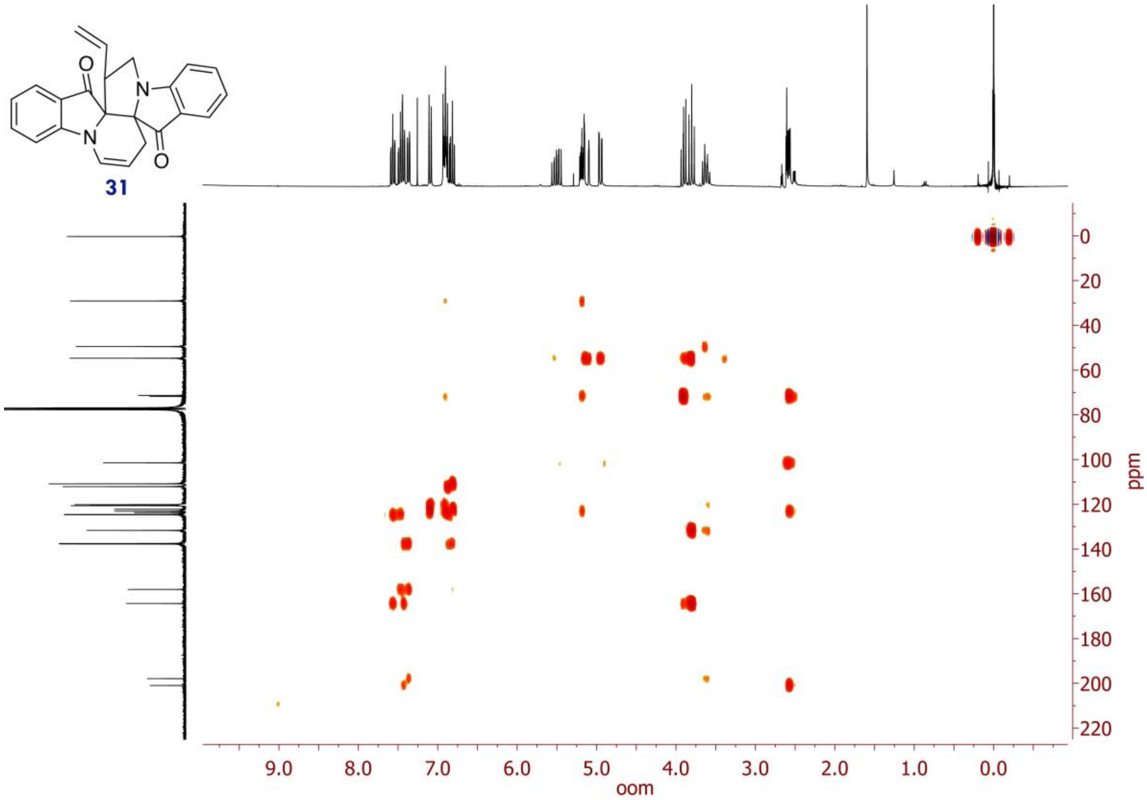

Figure S50: HMBC NMR spectrum, recorded in $CDCl_3$ for 1,2-dihydro-1-vinyl-8*H*,9*H*,17*H*-benz[2′,3′]pyrolizino[1′,7′a:2,3]pyrido[1,2-*a*]indole-8.17-dione **(31)**

UV–vis spectra of the new compounds

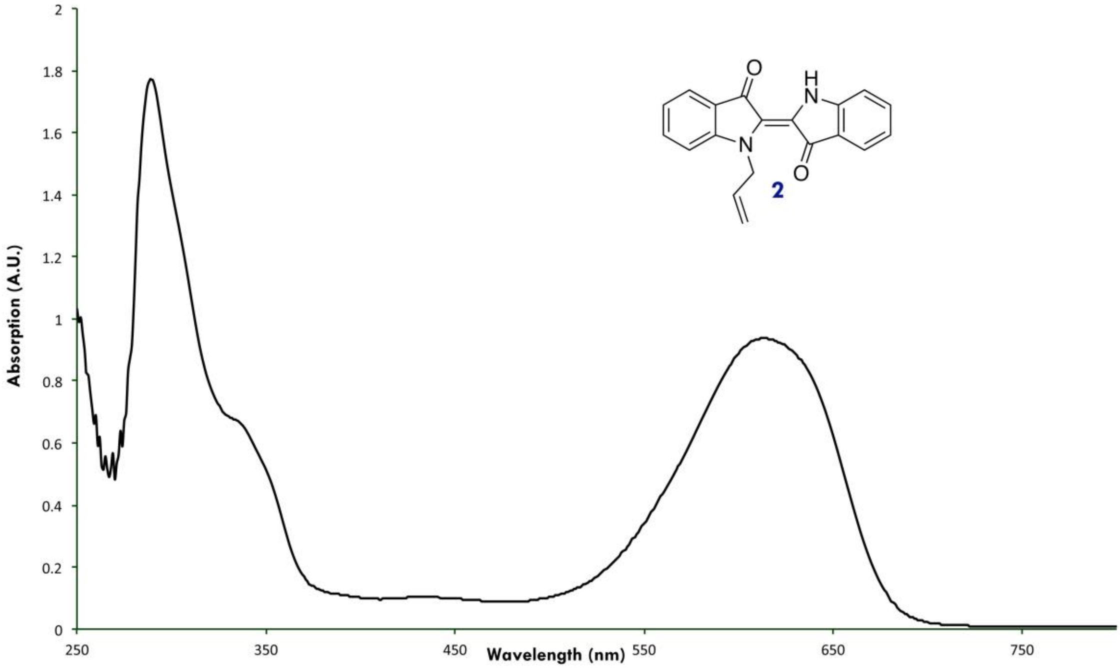

Figure S51: UV-vis spectrum, for (*E*)-1-allyl-[2,2'-biindolinylidene]-3,3'-dione **(2)** (78.6 µM) recorded in CH_2Cl_2

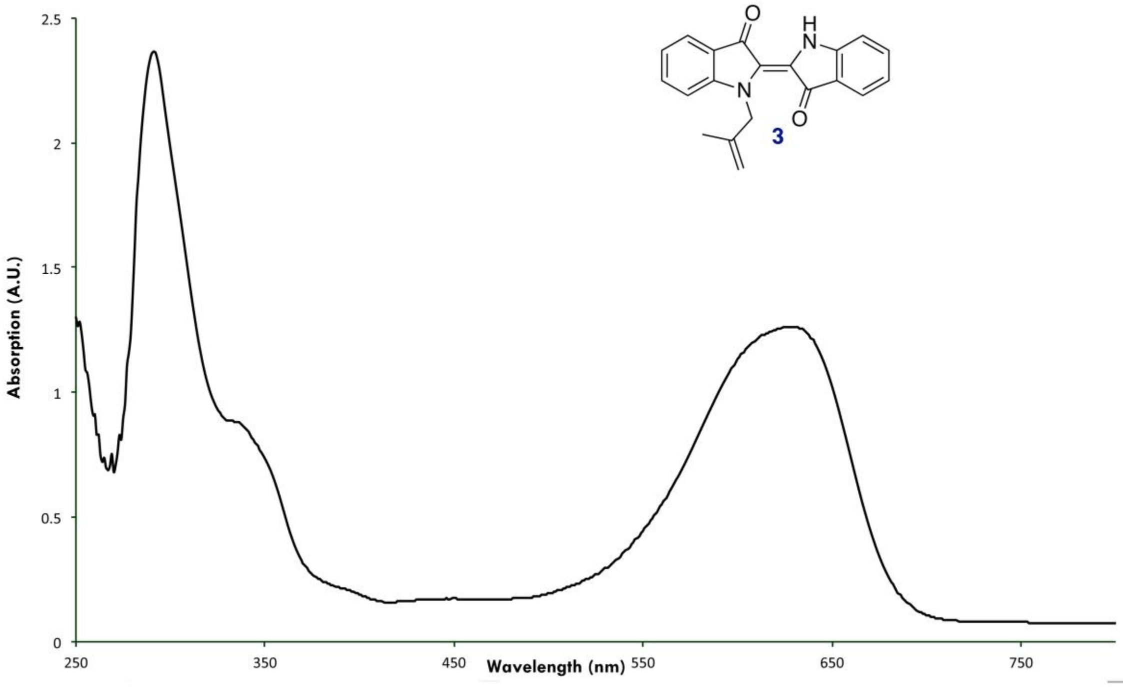

Figure S52: UV-vis spectrum for (*E*)-1-(2-methylallyl)-[2,2'-biindolinylidene]-3,3'-dione **(3)** (108.3 µM) recorded in CH_2Cl_2

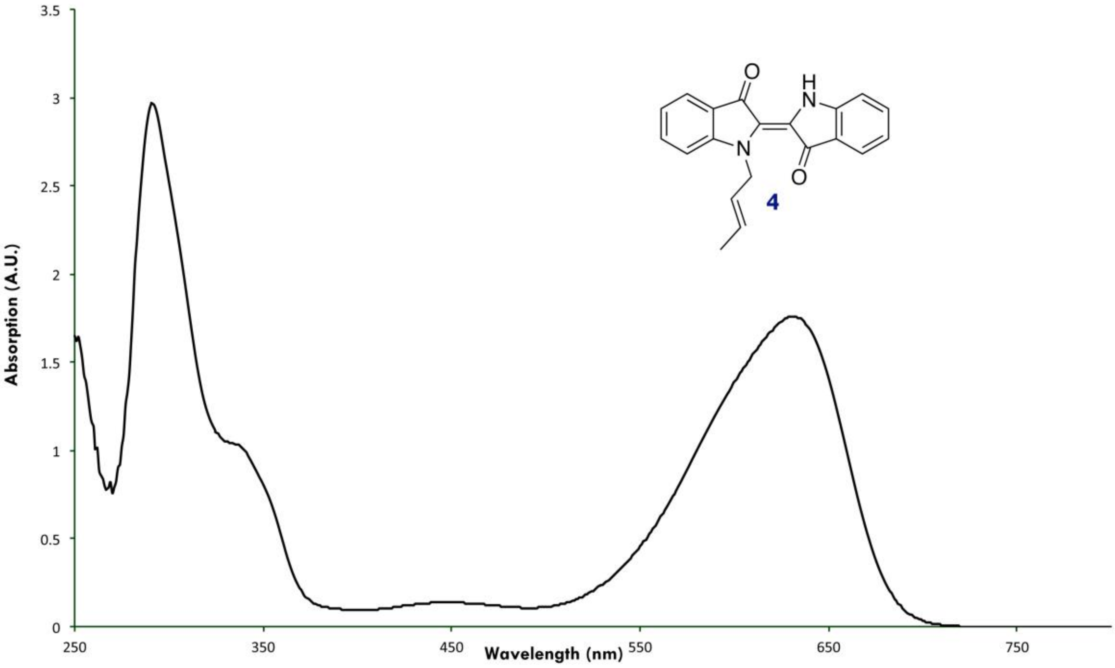

Figure S53: UV-vis spectrum, for (*E*)-1-((*E*)-but-2-en-1-yl)-[2,2'-biindolinylidene]-3,3'-dione **(4)**, (103.8 µM), recorded in CH_2Cl_2

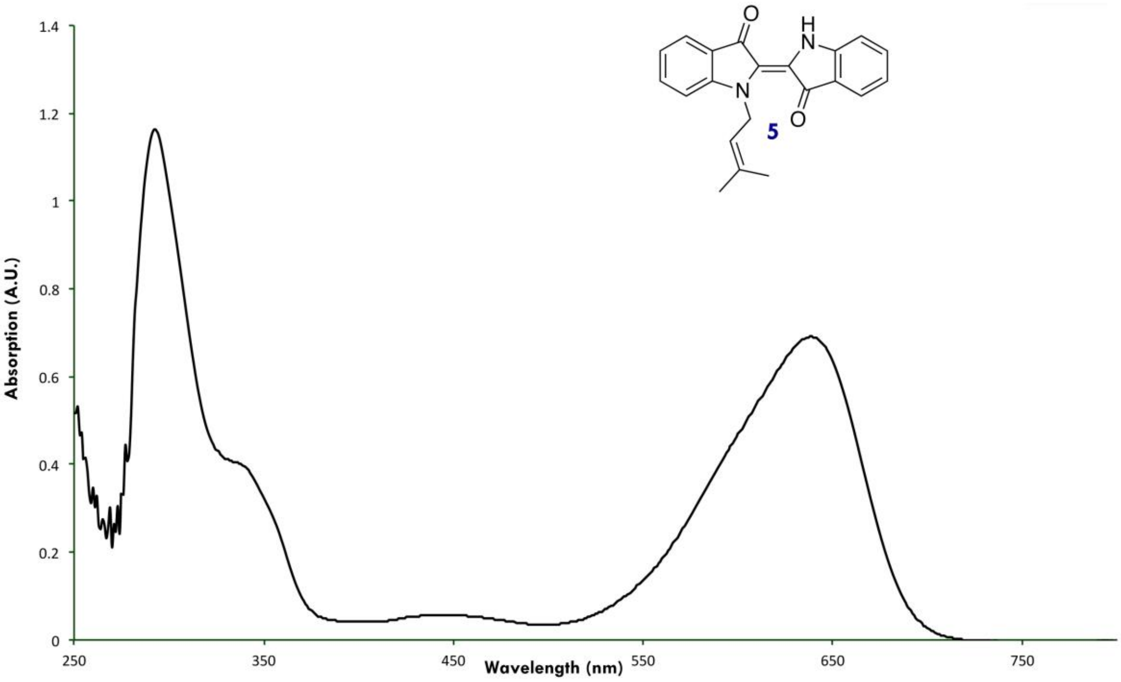

Figure S54: UV-vis spectrum for (*E*)-1-(3-methylbut-2-en-1-yl)-[2,2'-biindolinylidene]-3,3'-dione **(5)** (42.4 µM), recorded in CH_2Cl_2

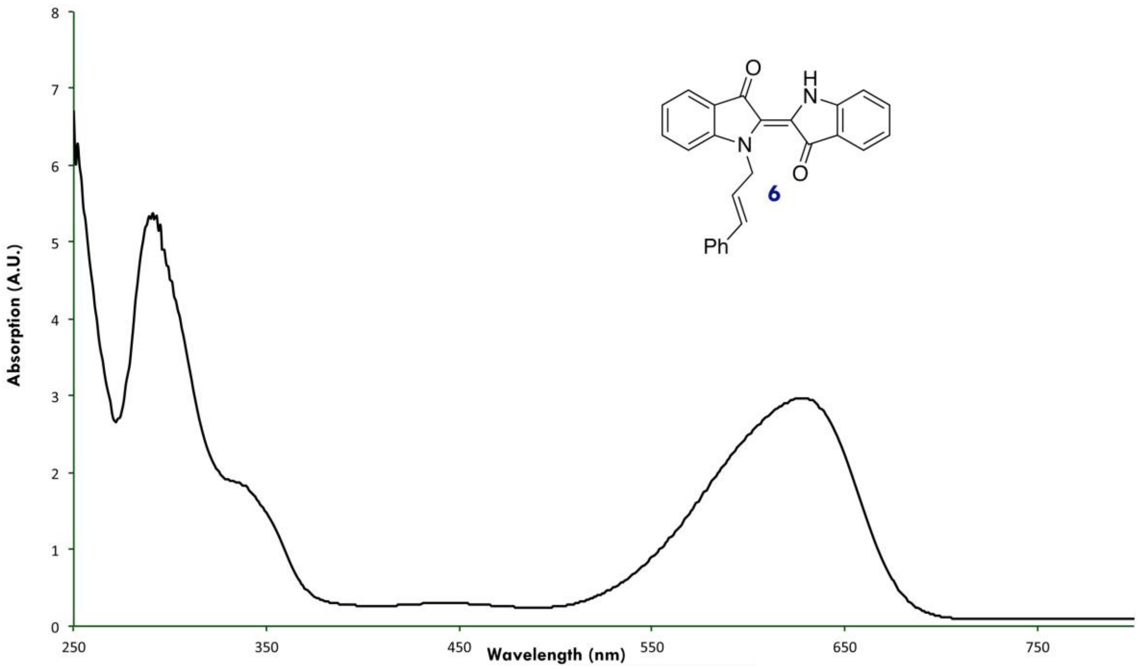

Figure S55: UV-vis spectrum for (*E*)-1-cinnamyl-[2,2'-biindolinylidene]-3,3'-dione **(6)** (73.4 µM), recorded in CH_2Cl_2

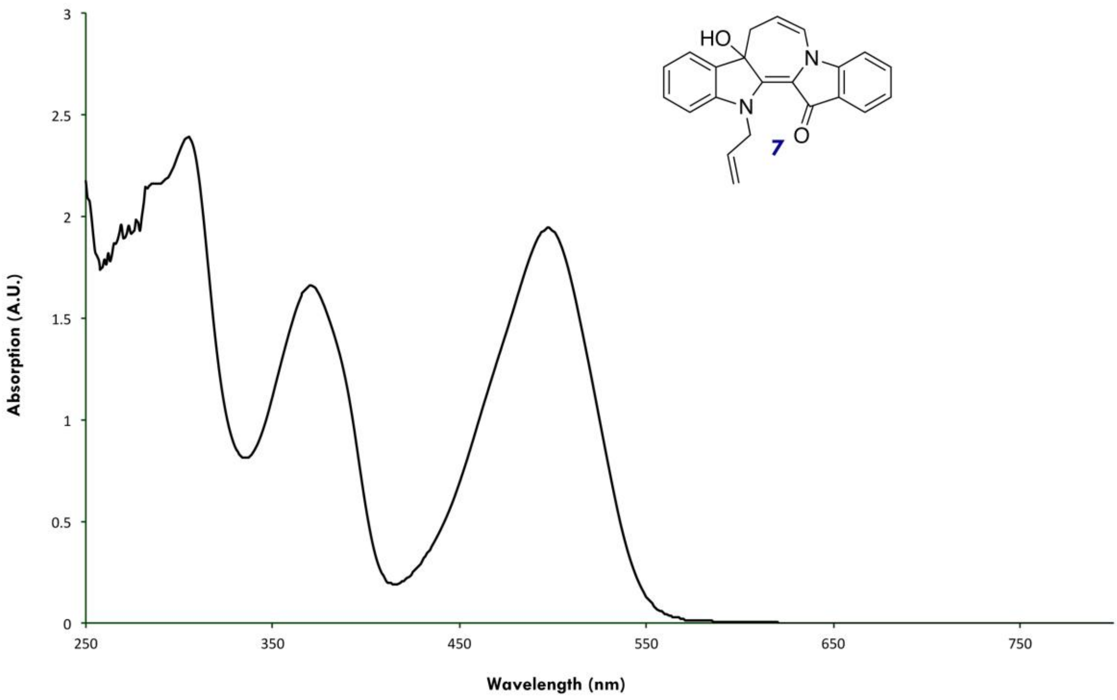

Figure S56: UV-vis spectrum for 13-allyl-8a-hydroxy-8,13a-dihydroazepino[1,2-*a*:3,4-*b'*]diindol-14(8*H*)-one **(7)** (164.3 µM), recorded in CH_2Cl_2

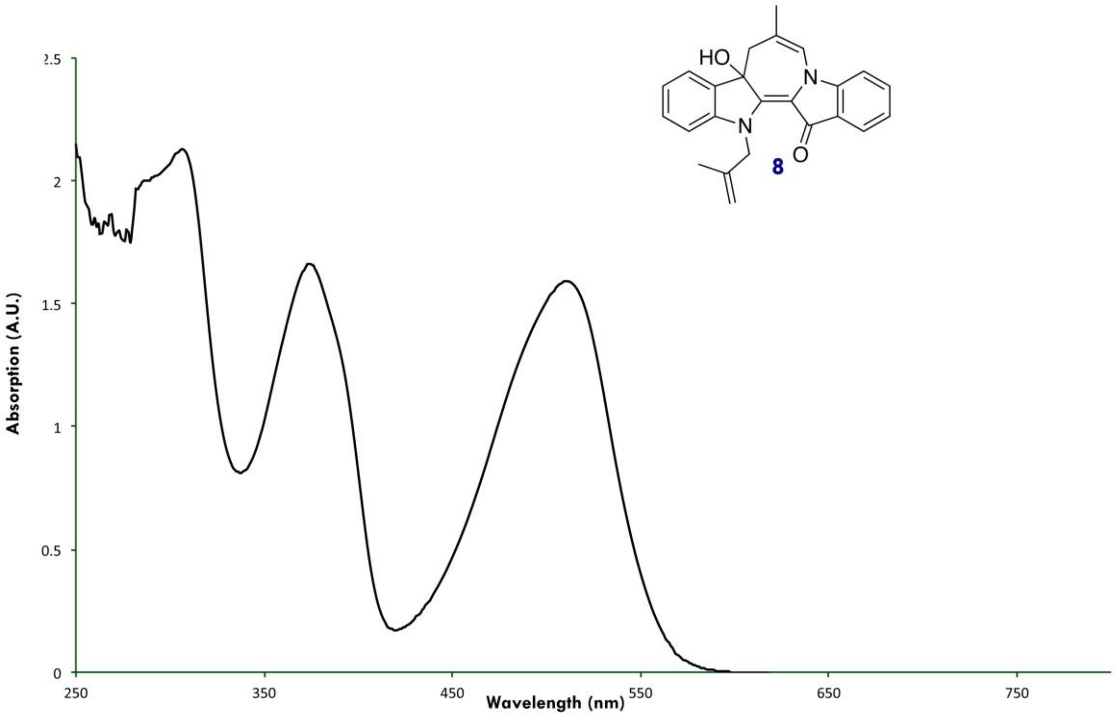

Figure S57: UV-vis spectrum for 8a-hydroxy-7-methyl-13-(2-methylallyl)-8,13a-dihydroazepino[1,2-*a*:3,4-*b*′]diindol-14(8*H*)-one **(8)** (152.0 µM), recorded in CH_2Cl_2

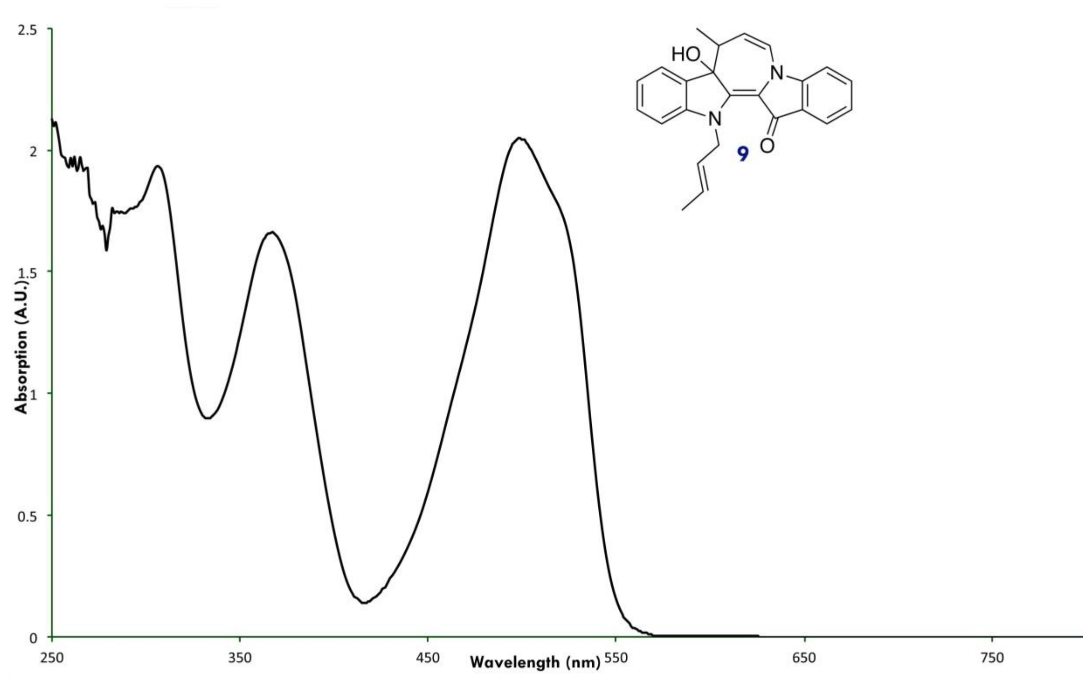

Figure S58: UV-vis spectrum for (*E*)-13-(but-2-en-1-yl)-8a-hydroxy-8-methyl-8,13a-dihydroazepino[1,2-*a*:3,4-*b*′]diindol-14(8*H*)-one **(9)** (142.7 µM), recorded in CH_2Cl_2

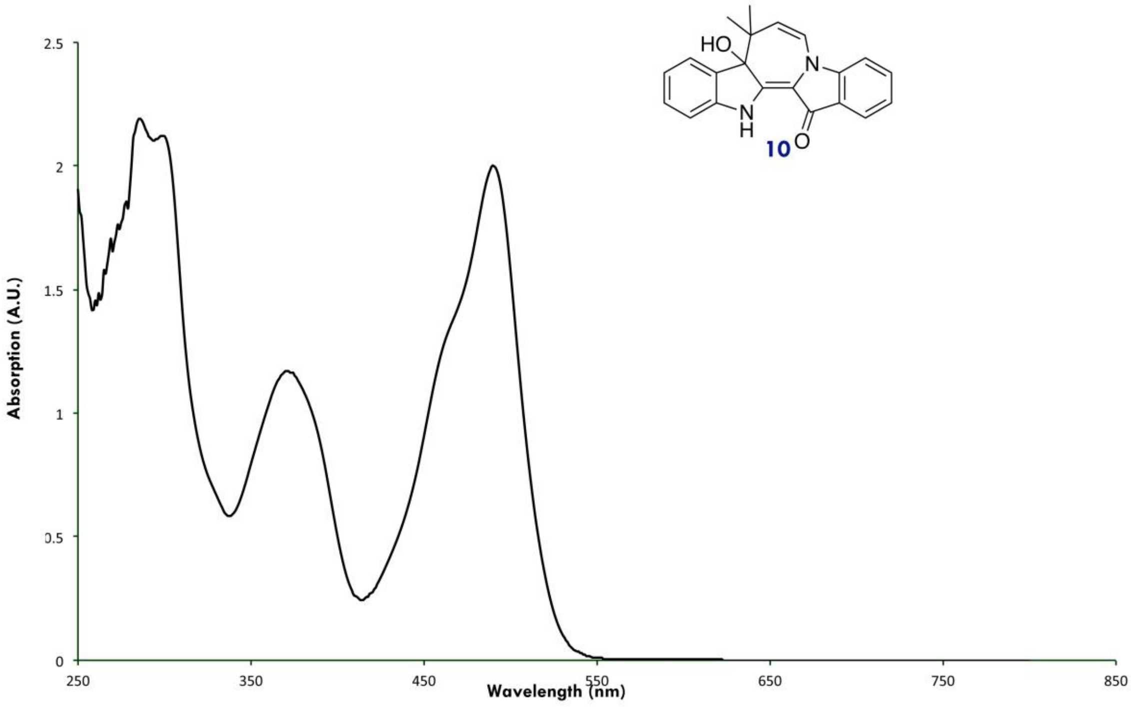

Figure S59: UV-vis spectrum for 8a-hydroxy-8,8-dimethyl-8,13a-dihydroazepino[1,2-*a*:3,4-*b'*]diindol-14(8*H*)-one **(10)** (131.2 µM), recorded in CH_2Cl_2

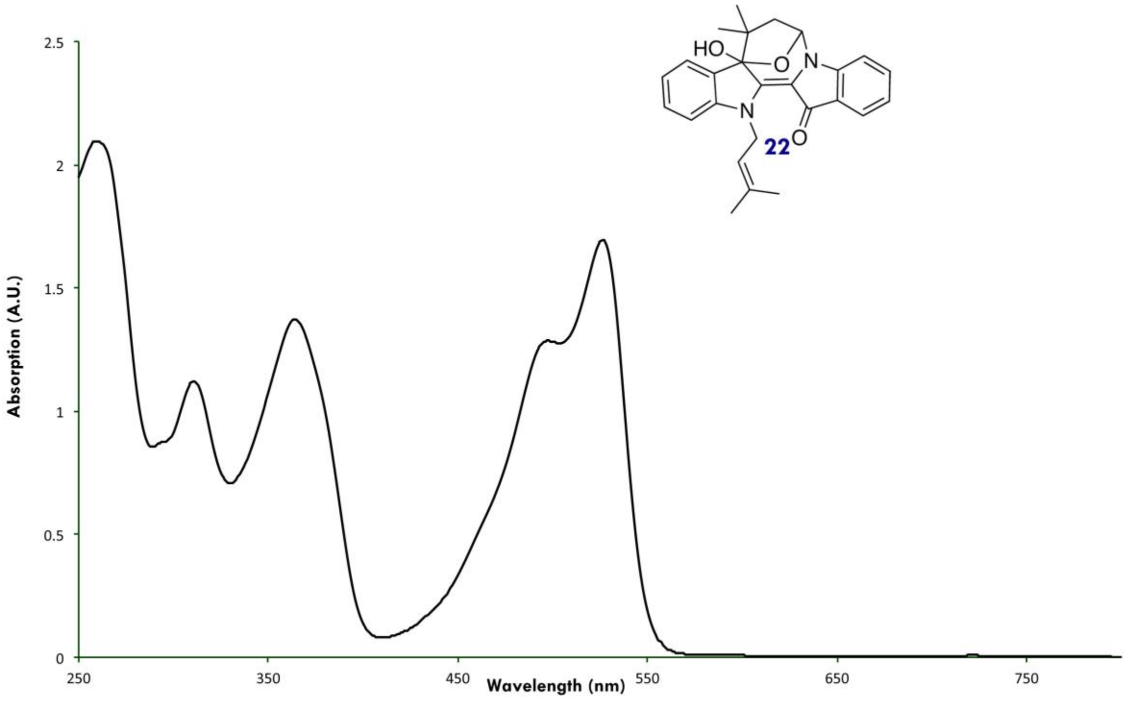

Figure S60: UV-vis spectrum for 8,8-dimethyl-13-(3-methylbut-2-en-1-yl)-7,8-dihydro-6*H*-6,8a-epoxyazepino[1,2-*a*:3,4-*b'*]diindol-14(13*H*)-one **(22)** (141.1 µM), recorded in CH_2Cl_2

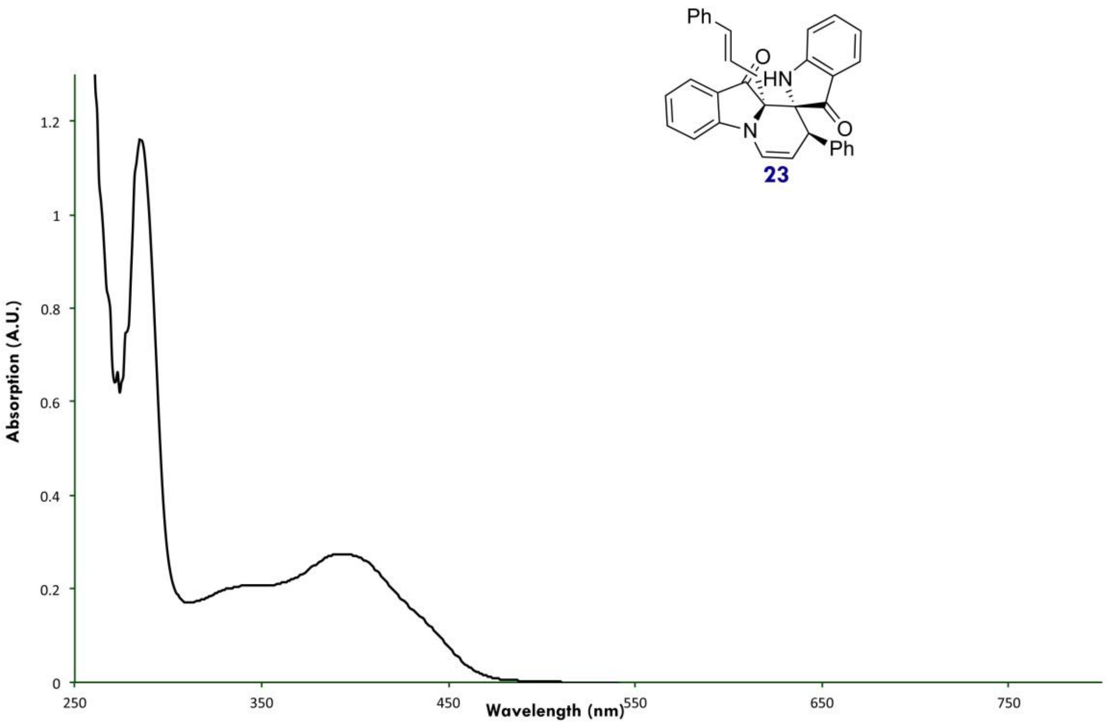

Figure S61: UV-vis spectrum for 9a'-cinnamyl-8'-phenyl-8'*H*-spiro[indoline-2,9'-pyrido[1,2-*a*]indole]-3,10'(9a'*H*)-dione **(23)** (7.54 µM), recorded in CH_2Cl_2

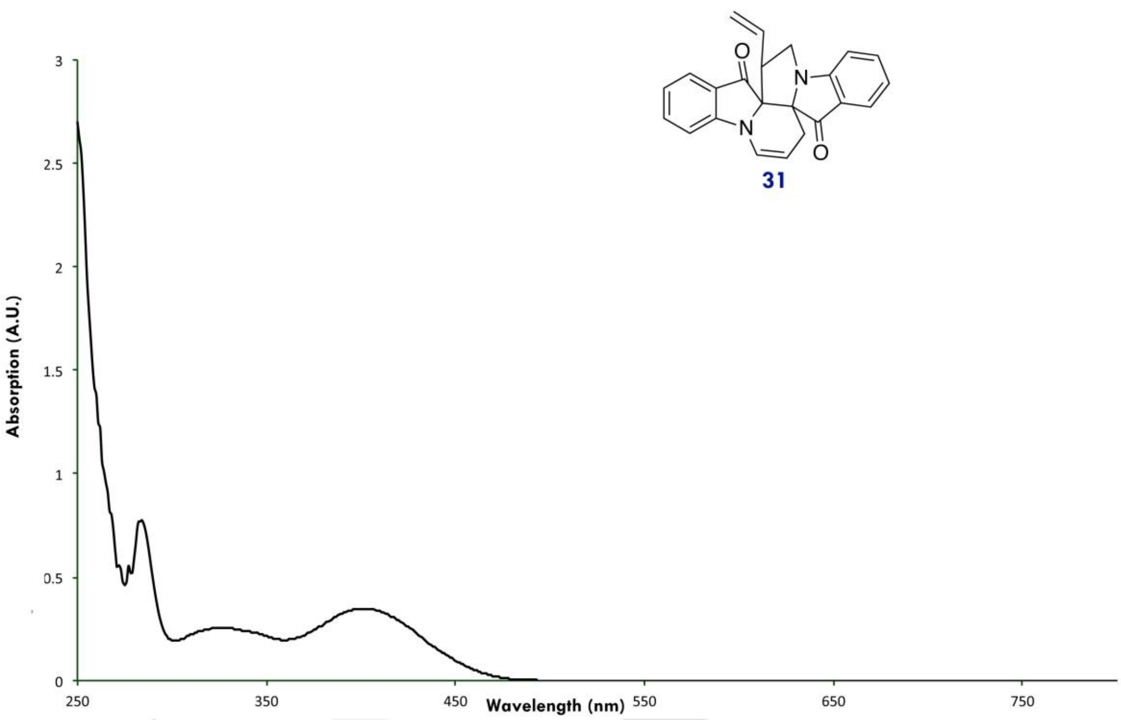

Figure S62: UV-vis spectrum for 6,7-dihydro-7-vinyl-8*H*,16*H*,17*H*-benz[2′,3′]pyrolizino[1′,7′a:2,3]pyrido[1,2-*a*]indole-8.17-dione **(31)** (8.96 µM), recorded in CH_2Cl_2

Crystallographic Studies

Crystallographic Data for Compounds 2, 7, 8, 9, 10, 23 and 31

Compound 2: $C_{22}H_{18}N_2O_2$, M = 342.40, T = 200 K, monoclinic, space group $P2_1/n$, Z = 4, a = 11.888(2), b = 11.667(2), c = 12.5729(17) Å, β = 107.386(11) °; V = 1664.1(5) Å^3, D_x = 1.367 g cm^{-3}, 2915 unique data ($2\theta_{max}$ = 50 °), R = 0.095 [for 1849 reflections with $I > 2.0\sigma(I)$]; Rw = 0.273 (all data), S = 1.03.

Compound 7: $C_{19}H_{14}N_2O_2$, M = 302.33, T = 200 K, orthorhombic, space group $Pna2_1$, Z = 4, a = 10.0417(3), b = 24.1863(5), c = 5.9097(2) Å; V = 1435.30(7) Å^3, D_x = 1.399 g cm^{-3}, 1799 unique data ($2\theta_{max}$ = 55 °), R = 0.040 [for 1359 reflections with $I > 2.0\sigma(I)$]; Rw = 0.088 (all data), S = 1.01.

Compound 8: $C_{24}H_{22}N_2O_2$, M = 370.45, T = 200 K, monoclinic, space group $C2/c$, Z = 8, a = 45.0292(8), b = 11.1720(3), c = 7.4857(1) Å, β = 99.4875(12) °; V = 3714.29(13) Å^3, D_x = 1.325 g cm^{-3}, 4264 unique data ($2\theta_{max}$ = 55 °), R = 0.042 [for 3279 reflections with $I > 2.0\sigma(I)$]; Rw = 0.099 (all data), S = 0.98.

Compound 9: $C_{24}H_{22}N_2O_2$, M = 370.44, T = 200 K, monoclinic, space group $P2_1/n$, Z = 4, a = 12.3025(3), b = 10.3191(3), c = 15.2106(3) Å, β = 104.3577(14) °; V = 1870.68(8) Å^3, D_x = 1.315 g cm^{-3}, 4271 unique data ($2\theta_{max}$ = 55 °), R = 0.054 [for 3245 reflections with $I > 2.0\sigma(I)$]; Rw = 0.141 (all data), S = 0.96.

Compound 10: $C_{21}H_{18}N_2O_2$, M = 330.39, T = 200 K, monoclinic, space group $P2_1/c$, Z = 4, a = 6.2304(2), b = 17.3681(6), c = 14.6545(5) Å, β = 95.602(2) °; V = 1578.19(9) Å^3, D_x = 1.390 g cm^{-3}, 2787 unique data ($2\theta_{max}$ = 50 °), R = 0.043 [for 2321 reflections with $I > 2.0\sigma(I)$]; Rw = 0.114 (all data), S = 1.00.

Compound 23: $2(C_{34}H_{26}N_2O_2)\bullet C_2H_6OS$, M = 1067.32, T = 200 K, triclinic, space group P-1, Z = 2, a = 10.5227(3), b = 12.2715(5), c = 22.4496(9) Å, α = 87.0834(19), β = 88.275(2), ϒ = 75.022(2) °; V = 2796.35(18) Å^3, D_x = 1.268 g cm^{-3}, 7798 unique data ($2\theta_{max}$ = 46 °), R = 0.059 [for 4931 reflections with $I > 2.0\sigma(I)$]; Rw = 0.157 (all data), S = 0.94.

Compound 31: $C_{23}H_{18}N_2O_2$, M = 354.41, T = 200 K, orthorhombic, space group $Pna2_1$, Z = 8, a = 12.3861(4), b = 10.4051(3), c = 27.6772(2) Å; V = 3567.00(19) Å^3, D_x = 1.320 g cm^{-3}, 3221 unique data ($2\theta_{max}$ = 50 °), R = 0.056 [for 2316 reflections with $I > 2.0\sigma(I)$]; Rw = 0.140 (all data), S = 1.02.

ORTEP plots of the reported compounds along with their Cambridge Crystallographic Data Centre code

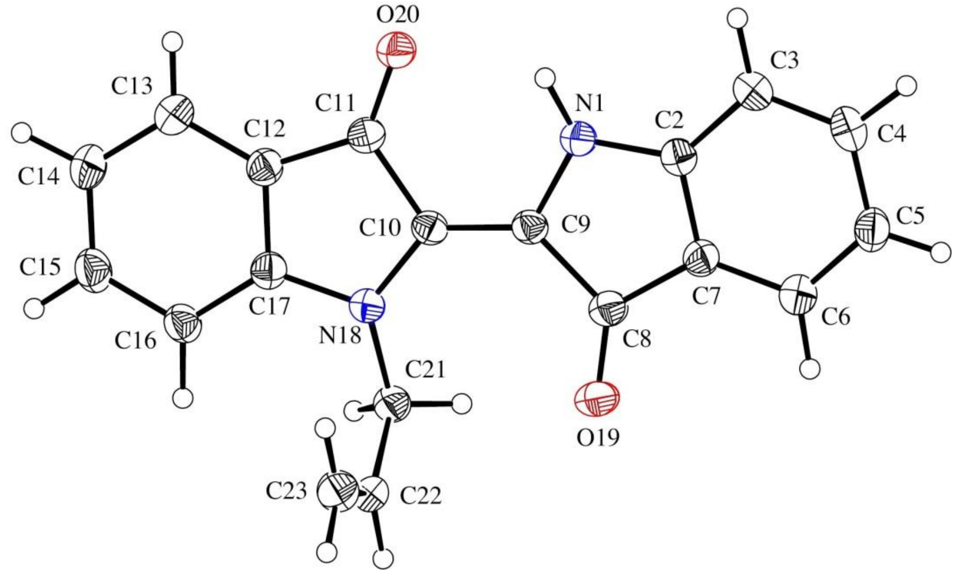

Figure S65: ORTEP plot and crystal data for compound (**2**) CCDC 986248

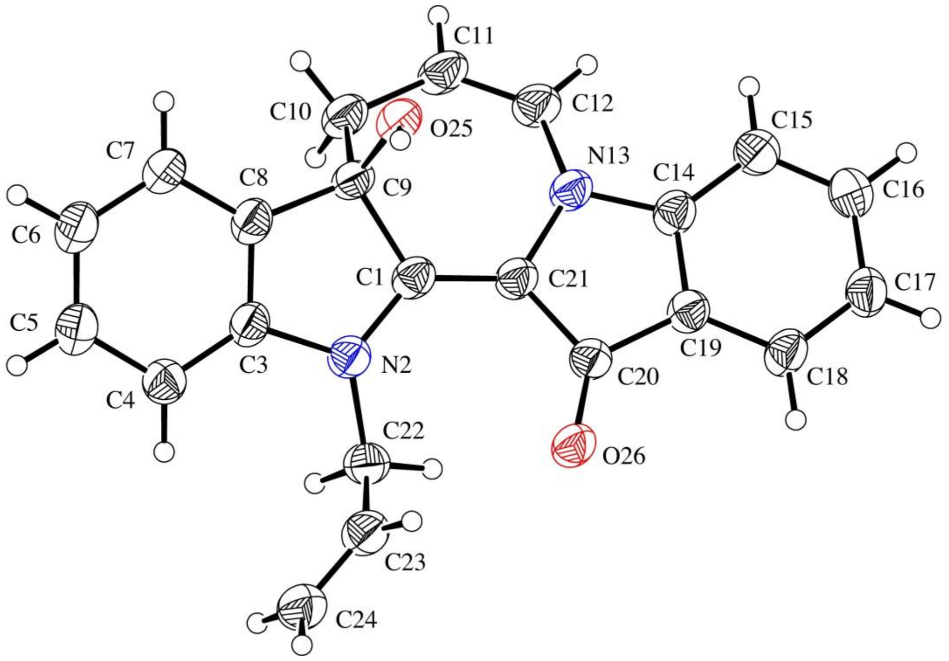

Figure S66: ORTEP plot and crystal data for compound (**7**) CCDC 986247

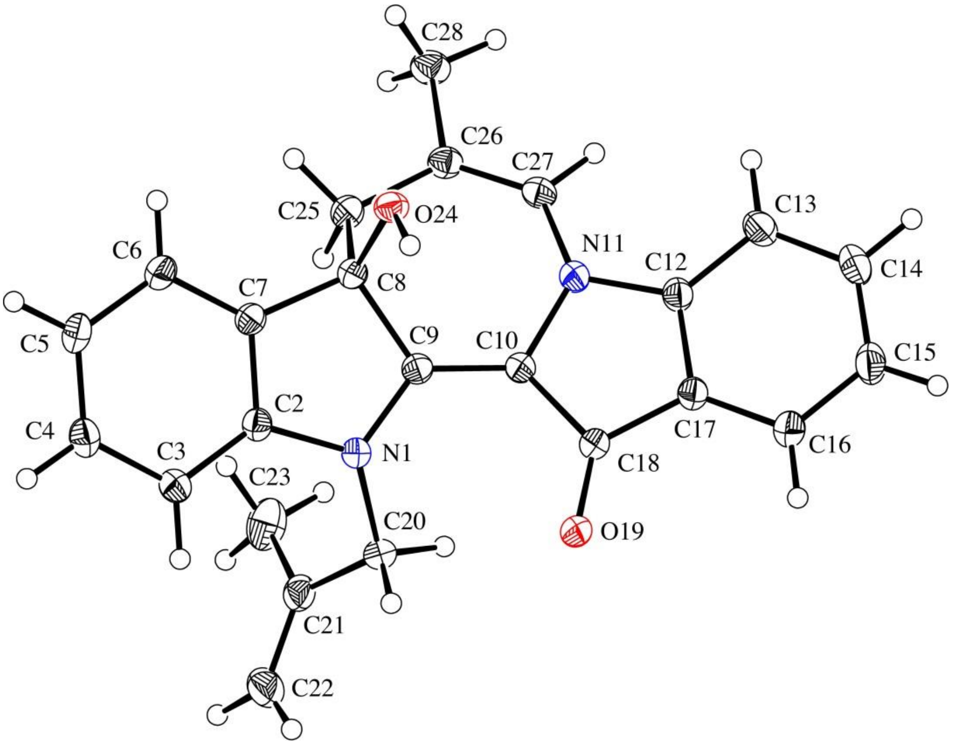

Figure S67: ORTEP plot and crystal data for compound (**8**) CCDC 986249

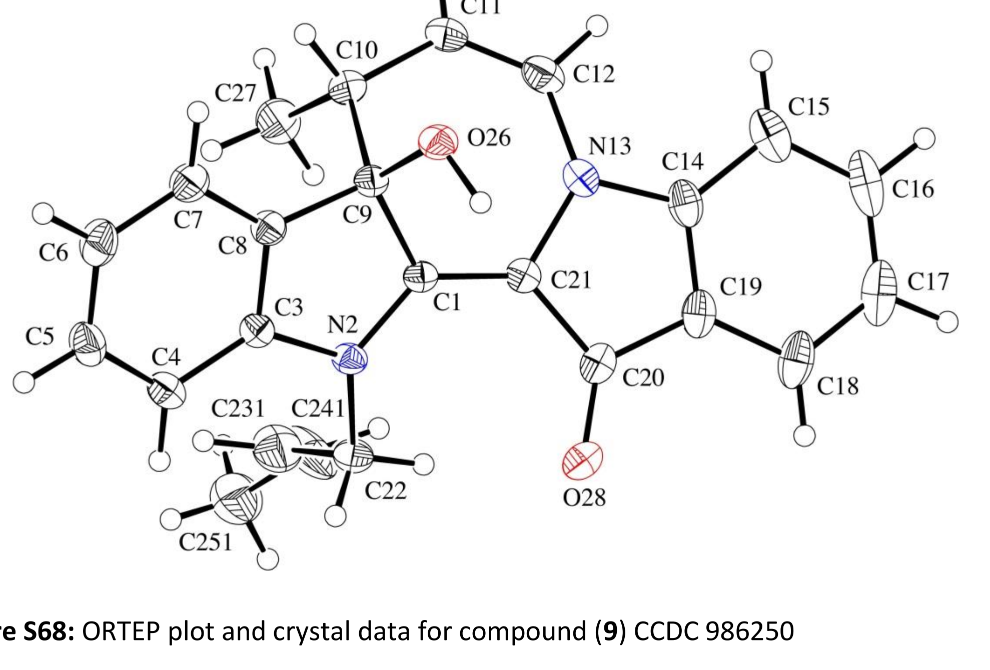

Figure S68: ORTEP plot and crystal data for compound (**9**) CCDC 986250

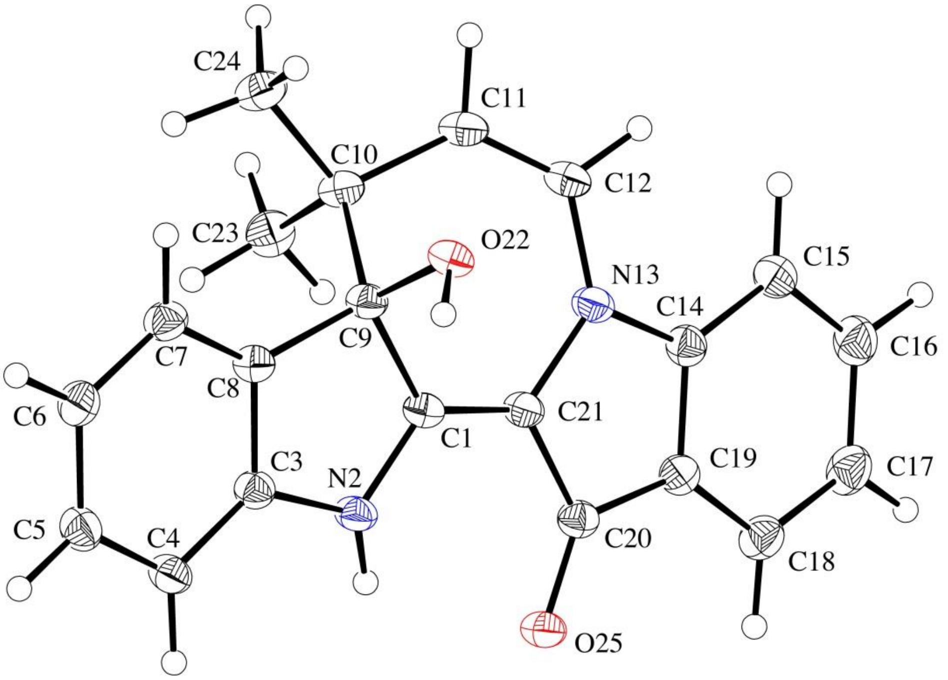

Figure S69: ORTEP plot and crystal data for compound (**10**) CCDC 986251

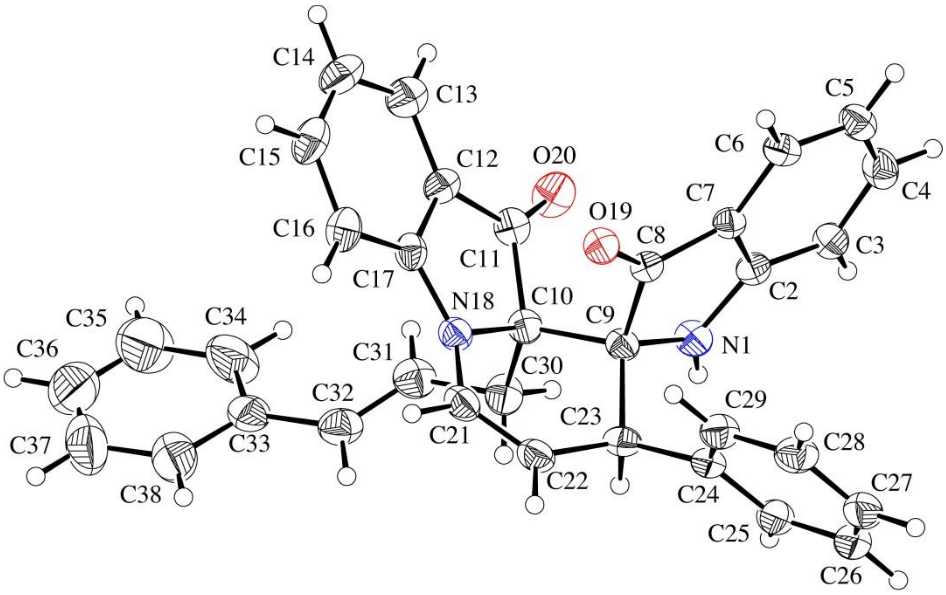

Figure S70: ORTEP plot and crystal data for compound (**23**) CCDC 986252

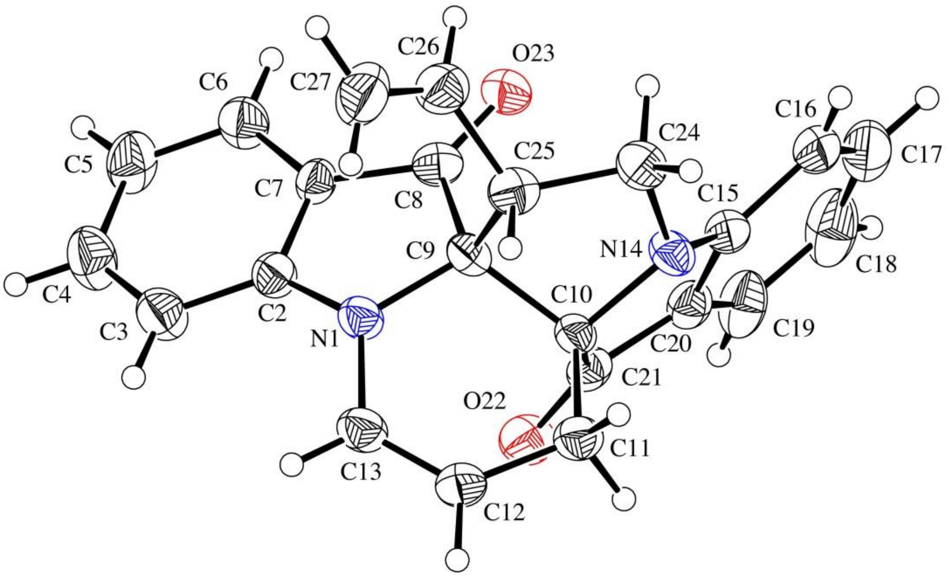

Figure S71: ORTEP plot and crystal data for compound (**31**) CCDC 986253

Supplementary photos

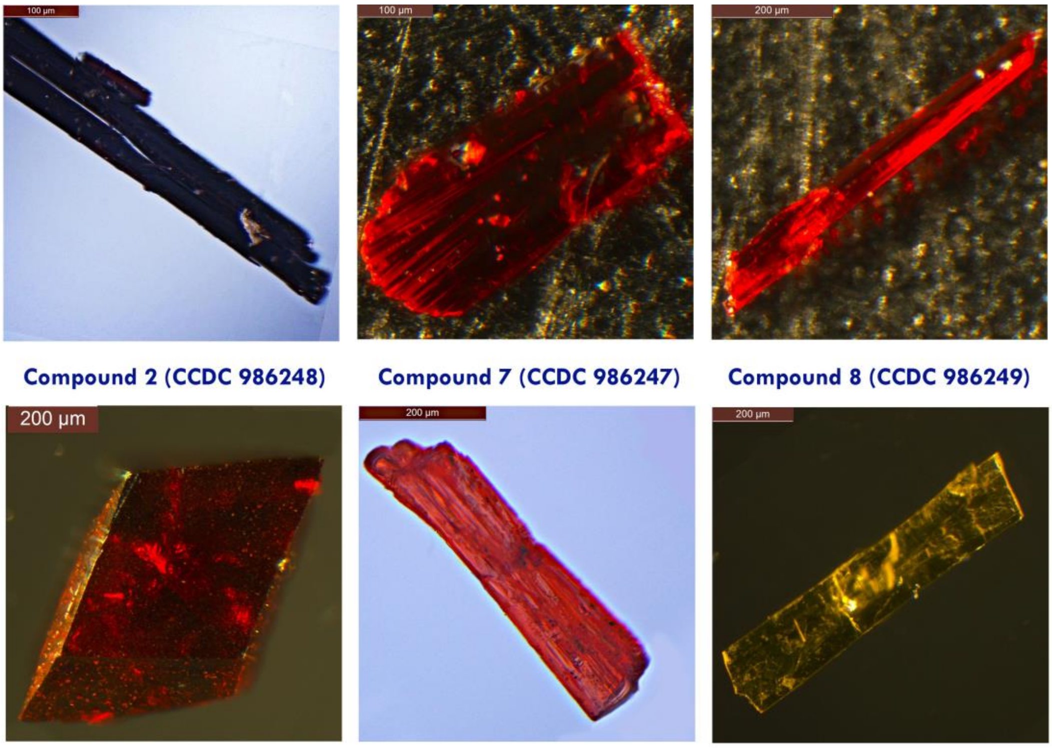

Compound 9 (CCDC 986250) Compound 10 (CCDC 986251) Compound 23 (CCDC 986252)

Figure S72: Microscopic picture of the corresponding crystals

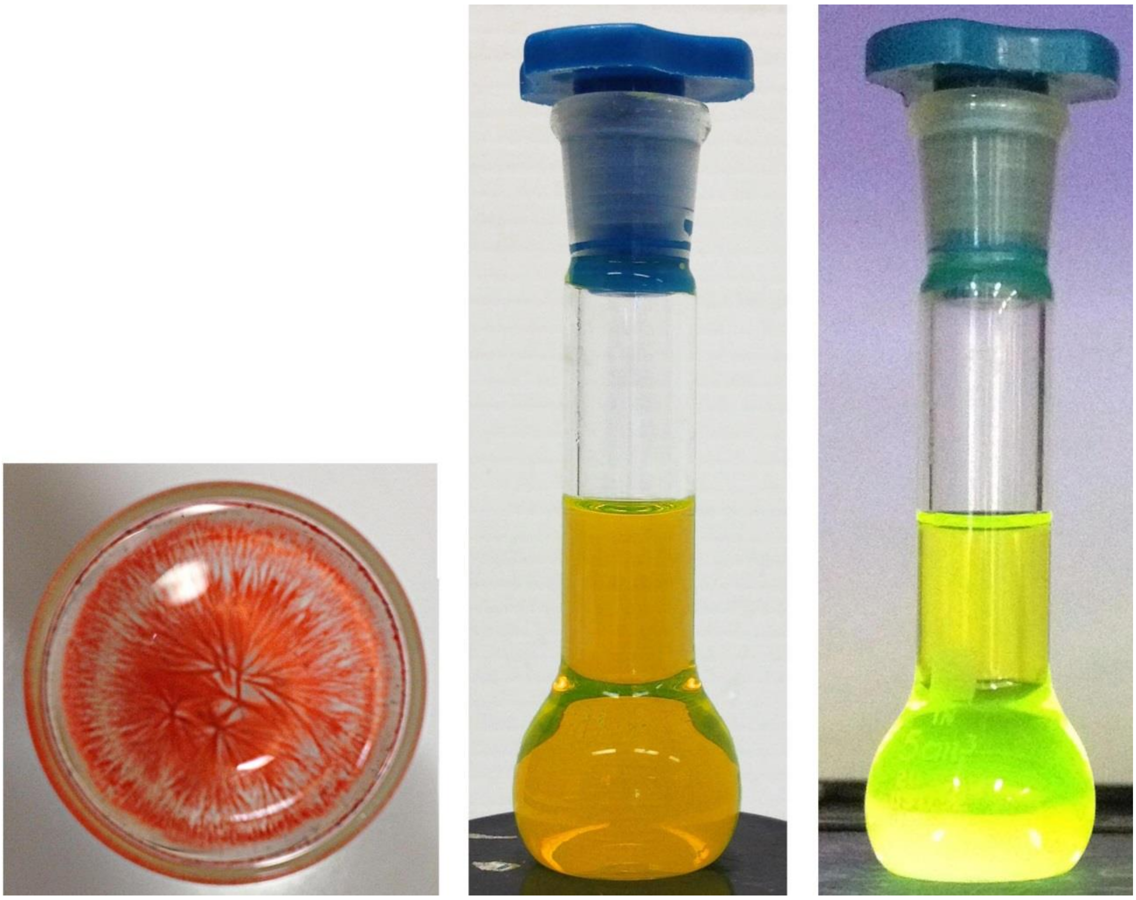

Figure S73: Colour variation of compound (**10**) from orange-red as solid (left) to green orange in solution under visible light (middle) and green fluorescence emission under 365 nm UV light (right)

References

1. DENZO–SMN. Otwinowski, Z.; Minor, W. Processing of X-ray diffraction data collected in oscillation mode. In *Methods in Enzymology*, Vol. 276: Macromolecular Crystallography, Part A; ed. C. W. Carter Jr., R. M. Sweet, Academic Press: New York, **1997**; pp. 307-326.

2. Altomare, A.; Cascarano, G.; Giacovazzo, C.; Guagliardi, A.; Burla, M. C.; Polidori, G.; Camalli, M. J. *Appl. Crystallogr.* **1994**, *27*, 435-436.

3. Betteridge, P. W.; Carruthers, J. R.; Cooper, R. I.; Prout, K.; Watkin, D. J. *J. Appl. Crystallogr.* **2003**, *36*, 1487.

4. Abdel-Hamid, M. K.; Bremner, J. B.; Coates, J.; Miländer, C.; Torkamani, Y. S.; Skelton, B. W.; White, A. H.; Willis A. C.; Keller, P. A. *Tetrahedron Lett.,* **2009**, *50*, 6947-6950.
